# *In silico* mouse study identifies tumour growth kinetics as biomarkers for the outcome of anti-angiogenic treatment

**DOI:** 10.1098/rsif.2018.0243

**Published:** 2018-08-22

**Authors:** Qianhui Wu, Alyssa D. Arnheim, Stacey D. Finley

**Affiliations:** 1 Department of Biomedical Engineering, University of Southern California, Los Angeles, CA, USA; 2 Department of Chemical Engineering and Materials Science, University of Southern California, Los Angeles, CA, USA; 3 Department of Biomedical Engineering, Boston University, Boston, MA, USA

**Keywords:** anti-angiogenic therapy, systems biology, computational modelling, tumour growth kinetics

## Abstract

Angiogenesis is a crucial step in tumour progression, as this process allows tumours to recruit new blood vessels and obtain oxygen and nutrients to sustain growth. Therefore, inhibiting angiogenesis remains a viable strategy for cancer therapy. However, anti-angiogenic therapy has not proved to be effective in reducing tumour growth across a wide range of tumours, and no reliable predictive biomarkers have been found to determine the efficacy of anti-angiogenic treatment. Using our previously established computational model of tumour-bearing mice, we sought to determine whether tumour growth kinetic parameters could be used to predict the outcome of anti-angiogenic treatment. A model trained with datasets from six *in vivo* mice studies was used to generate a randomized *in silico* tumour-bearing mouse population. We analysed tumour growth in untreated mice (control) and mice treated with an anti-angiogenic agent and determined the Kaplan–Meier survival estimates based on simulated tumour volume data. We found that the ratio between two kinetic parameters, *k*_0_ and *k*_1_, which characterize the tumour's exponential and linear growth rates, as well as *k*_1_ alone, can be used as prognostic biomarkers of the population survival outcome. Our work demonstrates a robust, quantitative approach for identifying tumour growth kinetic parameters as prognostic biomarkers and serves as a template that can be used to identify other biomarkers for anti-angiogenic treatment.

## 1. Background

Tumour angiogenesis results in the vascularization of a tumour. This process facilitates tumour growth by allowing tumour cells to obtain oxygen and nutrients through the newly formed blood vessels. As excessive vascularization is often seen in many types of cancer, inhibiting angiogenesis is thought to decrease tumour growth. Therefore, anti-angiogenic treatment is pursued as an attractive therapeutic strategy in oncology [1,2].

Bevacizumab is a humanized monoclonal antibody against vascular endothelial growth factor A (VEGF), a key angiogenic promoter in tumours [1]. This drug has been approved as a monotherapy or in combination with chemotherapy for many cancers, including renal cell carcinoma, metastatic colorectal cancer, non-small cell lung cancer and metastatic cervical cancer [3]. It also gained accelerated approval for treatment of metastatic breast cancer through the US Food and Drug Administration (FDA) in 2008. However, subsequent results showed that bevacizumab failed to improve overall survival and that the drug elicited significant adverse side effects. Consequently, the FDA revoked its approval for use of bevacizumab for first-line metastatic breast cancer in late 2011 [4,5]. Several Phase II and III clinical stage studies have also revealed contradicting results regarding the benefit of add-on bevacizumab in the neoadjuvant treatment setting for breast cancer patients [6–11]. Altogether, these studies illustrate that angiogenic therapy may not be effective across a wide range of patients. Indeed, breast cancer is a genetically and clinically heterogeneous cancer type, which makes identifying optimal therapies a challenge [12].

More broadly, there is a need for biomarkers to predict the response to treatment and identify the tumours for which anti-angiogenic treatment will be effective. A number of mechanistic biomarkers have been investigated for their ability to predict response to anti-angiogenic treatment and to determine an optimal treatment strategy. Promising biomarker candidates include the concentration ranges of circulating angiogenic molecules (such as plasma levels of VEGF) [13,14], tissue markers (tumour microvessel density) [15–18] and imaging parameters (magnetic resonance imaging-measured *K*^trans^) [15,19,20]. However, currently no validated and robust biomarkers are available that can guide selection of patients for whom anti-angiogenic therapy is most beneficial [5,15].

As an alternative, tumour growth kinetics may be used as biomarkers. There is a body of work that investigates how tumour growth kinetics can serve as prognostic biomarkers of the response to anti-angiogenic treatment [21–25]. Recently, a study showed that volume-based tumour growth kinetics may be a reliable indicator of treatment efficacy, and are in good agreement with standardized approaches for assessing response to treatment [21]. Moreover, we developed a computational systems biology model to further investigate the relationship between tumour growth kinetics and the response to anti-angiogenic therapy [26]. The model predicts VEGF distribution and kinetics in tumour-bearing mice, where the dynamic tumour volume is a function of the pro-angiogenic complexes involving VEGF-bound receptors (the ‘angiogenic signal’). By fitting the model to *in vivo* experimental data, we estimated the kinetic parameters that characterize tumour growth. We then used the trained model to predict the effect of anti-VEGF treatment on tumour volume, using only the estimated parameter values. The model predictions of tumour growth in response to anti-VEGF treatment closely matched experimental data. In this study, we concluded that there is a strong correlation between particular intrinsic kinetic parameters and the response to anti-VEGF treatment in terms of the end relative tumour volume (RTV).

Taking advantage of our established model framework and its strong predictive power, we now use this model to further investigate the utility of tumour growth kinetics to serve as a biomarker for anti-angiogenic treatment outcome. We performed an *in silico* randomized mouse study and estimated the survival of tumour-bearing mice in response to anti-VEGF treatment. Here, we introduced variability in the mouse population by allowing the tumour growth kinetic parameter values to vary within defined ranges. A total of 2400 mice with different tumour growth profiles were simulated in this study. By generating this large, heterogeneous *in silico* population of tumour-bearing mice, we can eliminate the likely bias caused by animals dropping out of experimental xenograft studies due to high tumour burden. In general, the average tumour size, particularly in the control group, can be underestimated in an experimental study, thereby underestimating the treatment effect, because large tumours are excluded from the analysis [27]. By contrast, computational modelling avoids these limitations and enables performance metrics (e.g. survival estimates) to be calculated [28]. Furthermore, computational systems biology is a powerful tool for studying how individual components contribute to the function and behaviour of a large system, and has been applied to study cancer at multiple scales [29–31]. Such computational models have been used to identify predictive biomarkers and to enhance the efficacy of anti-angiogenic therapies [13,32,33].

In our previous work, we did not explore how tumour growth parameters affect the response to treatment for individual tumours, nor did we examine time-based tumour growth inhibition. Therefore, in this study, we simulate the tumour volume over time in a heterogeneous population of mice and use more reliable and appropriate read-outs. Specifically, we performed time-to-event analysis [34] by determining the Kaplan–Meier survival curves based on the *in silico* population tumour growth data. We examined tumour growth kinetic parameters as prognostic biomarkers to distinguish the tumour response to anti-angiogenic treatment among the stratified groups.

## 2. Results

### 2.1. *In silico* mouse population tumour growth in the whole-body model

**Figure 1.**
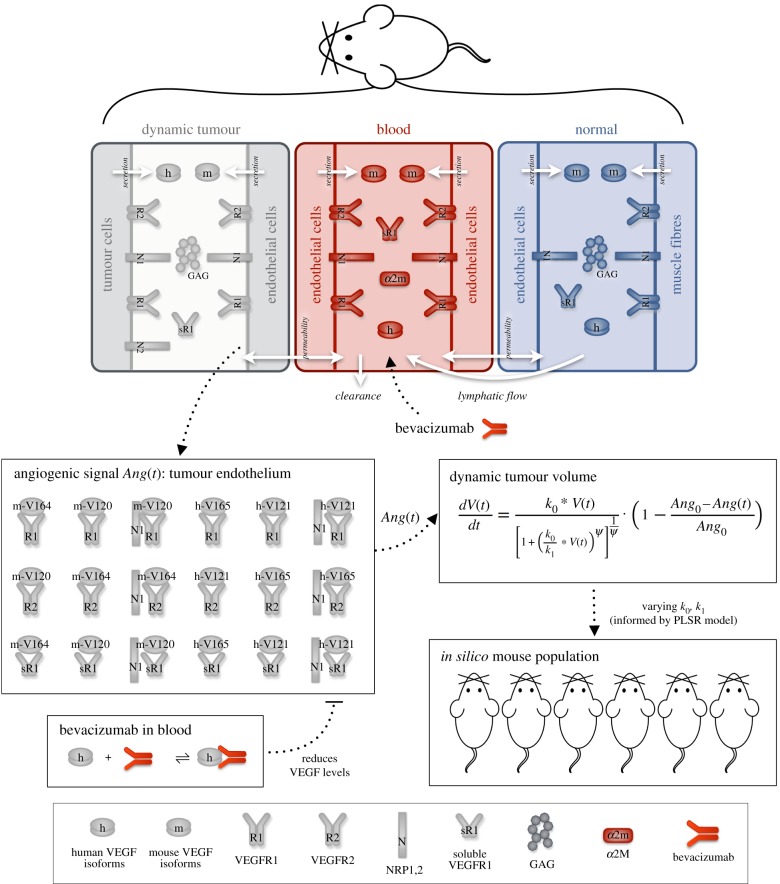
Schematic and overview of computational model of tumour-bearing mice. The three-compartment mouse model predicts VEGF binding kinetics and distribution in normal tissue, blood and tumour tissue. The model includes human (VEGF_121_ and VEGF_165_) and mouse (VEGF_120_ and VEGF_164_) VEGF isoforms, VEGF receptors (VEGFR1, sVEGFR1 and VEGFR2) and the protease inhibitor α-2-macroglobulin. The VEGF isoforms and sVEGFR1 can be transported between compartments via transendothelial macromolecular permeability and lymphatic flow. Species are also removed from the body via clearance. The pro-angiogenic signal (*Ang*(*t*)) is calculated as the summation of the concentrations of VEGF-bound receptor complexes in the tumour endothelium. The dynamic tumour volume is a function of the angiogenic signal, explicitly accounting for VEGF-mediated tumour growth. We previously estimated the tumour growth parameters (*k*_0_, *k*_1_, *ψ* and *Ang*_0_) by fitting the model to experimental data. In this study, we randomly varied tumour growth parameters within specified ranges to simulate tumour growth of several heterogeneous mouse populations. The anti-VEGF agent bevacizumab is used to simulate anti-angiogenic treatment via intravenous injections into the blood compartment. Bevacizumab inhibits the formation of pro-angiogenic complexes.

We performed an *in silico* randomized mouse study using our whole-body mouse model (figure 1). The model was previously fitted to each of six independent experimental datasets of control tumour volume in mice bearing MDA-MB-231 xenograft tumours and validated with a separate dataset [26]. The values of *k*_0_ and *k*_1_ (the rates of exponential and linear growth, respectively), and *Ang*_0_ (the basal angiogenic signal at time, *t* = 0) were estimated. A global sensitivity analysis indicated that *ψ* did not significantly influence tumour volume; thus, it was held constant. Here, we simulated the tumour growth of the six *in silico* populations of mice (henceforth referred to as ‘Roland’, ‘Zibara’, ‘Tan’, ‘Volk2008’, ‘Volk2011a’ and ‘Volk2011b’), with and without anti-VEGF treatment, in mice with different tumour growth kinetic parameters. For each population, the values of parameters *k*_0_ and *k*_1_ are randomly varied simultaneously with a uniform distribution within the ranges of their estimated values from our previous model fitting. Previously, a sensitivity analysis showed that the *Ang*_0_ parameter was an influential parameter to the model output when the model was fitted; however, further analysis using partial least-squares regression (PLSR) indicated that *Ang*_0_ was not a strong predictor of response to treatment [26]. Therefore, in each case, *Ang*_0_ is set as the median of the range of its estimated values. We generated 400 *in silico* mice for each of the six cases.

**Figure 2.**
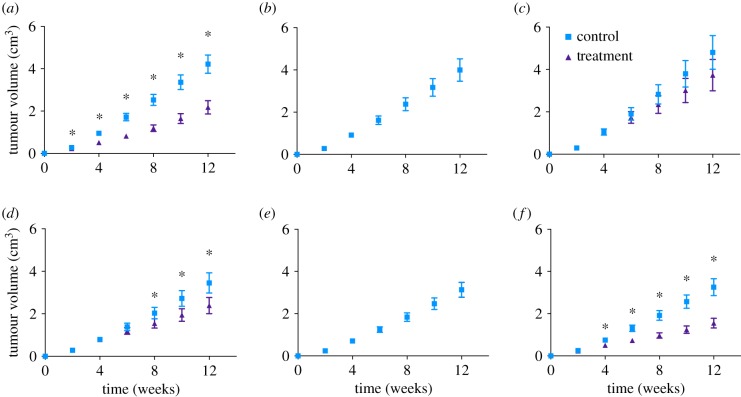
Model-simulated tumour growth data of *in silico* mouse populations. The whole-body mouse model previously fitted to each of the six datasets individually was used to simulate tumour volume over time. To generate the simulated tumours, the tumour growth kinetic parameters *k*_0_ and *k*_1_ were randomly varied within the range of the estimated values. A total of 400 simulations were run for each case. The mean and 95% confidence interval at each time point are shown. (*a*) Roland, (*b*) Zibara, (*c*) Tan, (*d*) Volk2008, (*e*) Volk2011a and (*f*) Volk2011b. Asterisks indicate that the difference between the control and treatment group tumour volumes is statistically significant (*p* < 0.05). (Online version in colour.)

Our simulations show that among the six cases, the anti-VEGF treatment has differential effects in reducing tumour growth, when compared with the control group (figure 2). For all cases, we used a single treatment protocol different from protocols used in each of the six experimental studies, in order to compare the predicted results without bias (termed ‘protocol A’). For Roland, Tan, Volk2008 and Volk2011b (figure 2*a,c,d,f*), the treated tumour volumes are less than the untreated tumours. Meanwhile, for Zibara and Volk2011a (figure 2*b,e*), there is no apparent difference in the tumour volumes for the treated and control groups. Thus, the model simulations reveal distinct differences in the effect of anti-VEGF treatment.

We further studied the effect of anti-VEGF treatment on tumour growth using RTV, the ratio between the mean tumour volumes of the treated and control groups. We calculated the RTV at each time point for all simulated tumours (electronic supplementary material, figure S1). We also determined the RTV at the end of treatment (electronic supplementary material, figure S2). The RTV values in all cases are smaller than one, indicating that the anti-VEGF treatment limits tumour growth, similar to what has been observed experimentally [35–39]. For Zibara and Volk2011a, the endpoint RTV values are just slightly less than one (electronic supplementary material, figure S2B,E), which is an expected result based on the similar tumour growth curves between the control and treated groups (figure 2*b,e*). Comparing the endpoint RTV among all six cases, the effect of anti-VEGF treatment in limiting tumour growth is the strongest for Volk2011b (RTV = 0.459 ± 0.054), followed by Roland (0.454 ± 0.096), Volk2008 (0.615 ± 0.066) and Tan (0.638 ± 0.049). This treatment effect is the least significant in Zibara (0.979 ± 0.009) and Volk2011a (0.987 ± 0.013).

### 2.2. Kinetic parameters as potential predictor for stratified population response

We investigated the relationship between the parameters that characterize tumour growth kinetics and the effect of the anti-VEGF treatment. Previously, our PLSR analysis indicated that for nearly all pairwise comparisons, if the RTV values for two datasets were significantly different, their *k*_0_/*k*_1_ ratios were also significantly different. This implies that *k*_0_/*k*_1_ is a large contributor in predicting the endpoint RTV [26]. Additionally, plotting the RTV versus *k*_0_, *k*_1_, and *k*_0_/*k*_1_ shows some relationship between the endpoint RTV and the tumour growth parameters (electronic supplementary material, figure S2). Therefore, we investigated whether these tumour growth parameters could stratify the simulated mouse populations, and distinguish their tumour growth and survival estimates. To address this question, we used our simulated tumour growth data for each case, noting the number of *in silico* mice at each time point. We record the time at which a mouse is ‘sacrificed’, which happens when the tumour volume reaches 2 cm^3^, as typically done in experimental studies [40]. This approach for modelling population survival allows us to closely mimic the practice in preclinical animal studies, and provides easily interpretable insights for researchers and clinicians.

**Figure 3.**
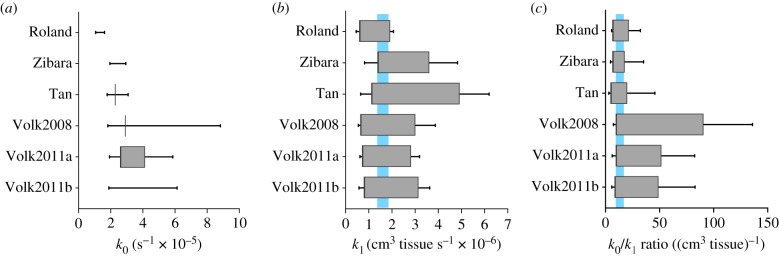
Range of parameter and threshold values. In each of the six cases, values of *k*_1*,*thresh_ and ratio_thresh__­­­_were found among all of the randomly generated values of *k*_1_ or the *k*_0_/*k*_1_ ratio used in the simulations. (*a*) *k*_0_, (*b*) *k*_1_ and (*c*) *k*_0_/*k*_1_ ratio. *Bars*: the ranges of all generated parameter values in each case. *Boxes*: the ranges of possible threshold values in each case. *Shading*: the common range of threshold values among the six cases. (Online version in colour.)

We used the simulated population survival data to determine if *k*_0_, *k*_1_ or *k*_0_/*k*_1_ can be used to discriminate between tumours for which anti-VEGF treatment is effective or not. We found that, in each case, a range of *k*_0_/*k*_1_ ratios, as well as *k*_1_, can be used to distinguish the population response to the anti-VEGF treatment (figure 3*b,c*). We term these ‘ratio_thresh_’ and ‘*k*_1*,*thresh_’ the values of the growth kinetic parameters that separate the simulated mouse population into groups with significantly different survival estimates. By contrast, we did not find any values of *k*_0_ alone that could be used to separate the simulated mouse population into groups whose survival estimates are statistically different for the Roland, Zibara and Volk2011b cases. For Tan and Volk2008, we only found one such *k*_0_ value in each case (figure 3*a*).

Interestingly, although the ranges of generated *k*_0_/*k*_1_ ratios and *k*_1_ were different for each of the six sets of tumour growth data, we found that there is an overlap among the potential ratio_thresh_ or *k*_1*,*thresh_ values found in each of the six cases. The common range of ratio_thresh_ is 9.757 to 17.982, and that of *k*_1*,*thresh_ is 1.391 × 10^−6^ to 1.931 × 10^−6^. This means that separating the treatment group by any *k*_1*,*thresh_ *or* ratio_thresh_ value within its respective range will produce two groups of treated mice that have statistically different survival estimates. Specifically, the treated group with *k*_0_/*k*_1_ ratios larger than ratio_thresh_ has a better survival estimate than the treated group with smaller ratios. The treated group with *k*_1_ smaller than *k*_1*,*thresh_ has a better survival estimate than the treated group with larger *k*_1_.

**Figure 4.**
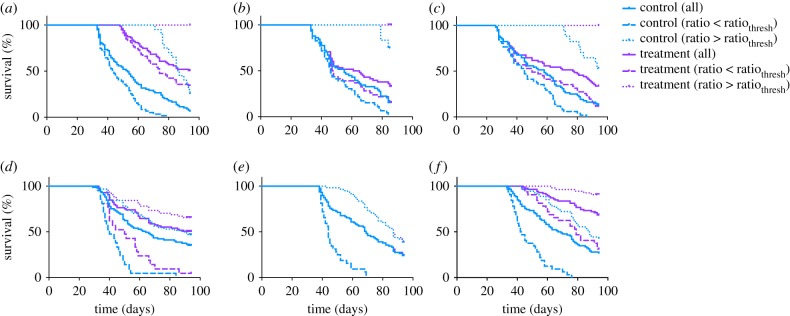
Kaplan–Meier curves for the six simulated groups of tumour-bearing mice. Here, the ratio_thresh_ value is taken as the median from the common range found among the six cases (13.8693). (*a*) Roland, (*b*) Zibara, (*c*) Tan, (*d*) Volk2008, (*e*) Volk2011a and (*f*) Volk2011b. The estimated survival curves of *in silico* mice subgroups within each group are shown in each plot: all mice, mice with ratio above or below the median ratio_thresh_ in the control setting or with treatment.

We used the median ratio_thresh_ value (13.689) to illustrate this distinction. We compare the survival estimates for a total of six groups: (i) all mice in the control group; (ii) all mice in the treatment group; (iii) control group with *k*_0_/*k*_1_ < ratio_thresh_; (iv) control group with *k*_0_/*k*_1_ > ratio_thresh_; (v) treatment group with *k*_0_/*k*_1_ < ratio_thresh_; and (vi) treatment group with *k*_0_/*k*_1_ > ratio_thresh_. We generated the Kaplan–Meier survival curves for these groups for each of the six cases investigated (figure 4). We also estimated the median survival of the six groups in each case (table 1), the Mantel–Haenszel hazard ratio (HR), with 95% confidence interval (CI), and the *p*-values from the Mantel–Cox log rank test for survival curve comparison (table 2). When comparing two groups, if the HR is less than one, the first group has a lower death rate (see Methods). Together these analyses emphasize that mice with larger *k*_0_/*k*_1_ ratios survive for longer, with *p*-value < 0.05. Interestingly, for Zibara and Volk2011a, although the anti-VEGF treatment does not significantly reduce tumour growth and therefore does not yield a better survival estimate for the treated groups compared to their control groups (figures 2*b,e* and 4*b,e*), the stratified groups yield significantly different survival estimates. That is, the control and treated groups with *k*_0_/*k*_1_ ratios larger than ratio_thresh_ have better survival estimates than those with smaller *k*_0_/*k*_1_ ratios.

**Table 1.** Summary of median survival of population separated by median ratio_thresh_.

median survival (days)	Roland^b^	Zibara^b^	Tan^b^	Volk 2008^b^	Volk 2011a^b^	Volk 2011b^b^	Zibara^c^	Volk 2011a^d^	Volk 2011a^e^	Mollard^b^
control (all)	53	55	58	64	69	68	55	69	69	85
control (*k*_0_/*k*_1_ < ratio_thresh_)	44	44.5	45	40	44	42.5	44.5	42.5	44	77
control (*k*_0_/*k*_1_ > ratio_thresh_)	86.5	84	Un^a^	89.5	87	88	84	80	87	96
treatment (all)	Un^a^	63	77.5	Un^a^	69	Un^a^	63	71	Un^a^	Un^a^
treatment (*k*_0_/*k*_1_ < ratio_thresh_)	74	46	50	50	44	79	46	42.5	78.5	Un^a^
treatment (*k*_0_/*k*_1_ > ratio_thresh_)	Un^a^	Un^a^	Un^a^	Un^a^	87	Un^a^	Un^a^	87	Un^a^	Un^a^

^a^Un, undefined. The median survival cannot be estimated if the survival estimation does not reach below 50%.

^b^Protocol A: biweekly treatment at a dosage of 10 mg kg^−1^, starting when the tumour volume reaches 0.1 cm^3^.

^c^Protocol Z: biweekly treatment at a dosage of 10 mg kg^−1^, starting when the tumour volume is 0.004 cm^3^ (upon engraftment of tumour).

^d^Protocol V11a: biweekly treatment at a dosage of 10 mg kg^−1^, starting when the tumour volume reaches 0.5 cm^3^.

^e^Protocol V11a-D: biweekly treatment at a dosage of 20 mg kg^−1^, starting when the tumour volume reaches 0.5 cm^3^.

**Table 2.** Statistics comparing the Kaplan–Meier survival curves of the population separated by median ratio_thresh_: hazard ratio (95%CI) and log rank test *p*-values.

HR (95% CI) *p*-value	Roland^a^	Zibara^a^	Tan^a^	Volk2008^a^	Volk2011a^a^
treatment (*k*_0_/*k*_1_ > ratio_thresh_) versus treatment (*k*_0_/*k*_1_ < ratio_thresh_)	0.2073 (0.1059–0.4057) *p* < 0.0001	0.2005 (0.0991–0.4055) *p* < 0.0001	0.1623 (0.0872–0.3021) *p* < 0.0001	0.0576 (0.0237–0.1399) *p* < 0.0001	0.0216 (0.0098–0.0476) *p* < 0.0001
control (*k*_0_/*k*_1_ > ratio_thresh_) versus control (*k*_0_/*k*_1_ < ratio_thresh_)	0.1214 (0.06974–0.2144) *p* < 0.0001	0.1627 (0.0866–0.3056) *p* < 0.0001	0.1445 (0.0808–0.2582) *p* < 0.0001	0.0422 (0.0173–0.1035) *p* < 0.0001	0.0296 (0.0098–0.0476) *p* < 0.0001
treatment (*k*_0_/*k*_1_ > ratio_thresh_) versus control (*k*_0_/*k*_1_ > ratio_thresh_)	0.0675 (0.0234–0.1949) *p* < 0.0001	0.1191 (0.01194–1.188) *p* = 0.0697	0.101 (0.0249–0.4103) *p* = 0.0013	0.5683 (0.3349–0.9643) *p* = 0.0362	0.9921 (0.6117–1.609) *p* = 0.9742
treatment (*k*_0_/*k*_1_ > ratio_thresh_) versus treatment (all)	0.2562 (0.1239–0.5299) *p* = 0.0002	0.2538 (0.1191–0.5408) *p* = 0.0004	0.239 (0.1236–0.4621) *p* < 0.0001	0.6138 (0.3732–1.01) *p* = 0.0546	0.576 (0.3863–0.8588) *p* = 0.0068
treatment (all) versus control (all)	0.2307 (0.1548–0.3438) *p* < 0.0001	0.6742 (0.4384–1.037) *p* = 0.0726	0.5845 (0.3934–0.8684) *p* = 0.0079	0.6481 (0.4331–0.9699) *p* = 0.0350	0.9959 (0.7038–1.409) *p* = 0.9815
treatment (*k*_0_/*k*_1_ < ratio_thresh_) versus treatment (all)	1.569 (0.9721–2.41) *p* = 0.0549	1.558 (0.9714–2.5) *p* = 0.0658	1.794 (1.158–2.778) *p* = 0.0089	6.405 (2.971–13.81) *p* < 0.0001	7.657 (4.065–14.42) *p* < 0.0001

HR (95% CI) *p*-value	Volk2011b^a^	Zibara^b^	Volk2011a^c^	Volk2011a^d^	Mollard^a^
treatment (*k*_0_/*k*_1_ > ratio_thresh_) versus treatment (*k*_0_/*k*_1_ < ratio_thresh_)	0.0673 (0.0288–0.157) *p* < 0.0001	0.2332 (0.1191–0.4566) *p* < 0.0001	0.01104 (0.0039–0.0315) *p* < 0.0001	0.358 (0.0157–0.0815) *p* < 0.0001	0.1862 (0.1043–0.3327) *p* < 0.0001
control (*k*_0_/*k*_1_ > ratio_thresh_) versus control (*k*_0_/*k*_1_ < ratio_thresh_)	0.0368 (0.01750–0.0778) *p* < 0.0001	0.1655 (0.0925–0.2962) *p* < 0.0001	0.0110 (0.0039–0.0315) *p* < 0.0001	0.0418 (0.0204–0.0857) *p* < 0.0001	0.6573 (0.5219–0.8029) *p* < 0.0001
treatment (*k*_0_/*k*_1_ > ratio_thresh_) versus control (*k*_0_/*k*_1_ > ratio_thresh_)	0.1592 (0.0817–0.3102) *p* < 0.0001	0.1707 (0.0557–0.5226) *p* = 0.002	0.7027 (0.4718–1.047) *p* = 0.0825	0.0842 (0.0452–0.1568) *p* < 0.0001	0.0782 (0.0561–0.1089) *p* < 0.0001
treatment (*k*_0_/*k*_1_ > ratio_thresh_) versus treatment (all)	0.3364 (0.1662–0.6806) *p* = 0.0024	0.2513 (0.1294–0.4881) *p* < 0.0001	0.7306 (0.5008–1.066) *p* = 0.1033	0.2524 (0.126–0.5058) *p* = 0.0001	0.317 (0.176–0.571) *p* = 0.0001
treatment (all) versus control (all)	0.266 (0.1733–0.4083) *p* < 0.0001	0.6742 (0.4384–1.037) *p* = 0.0726	0.7766 (0.5543–1.088) *p* = 0.1416	0.2016 (0.1343–0.3027) *p* < 0.0001	0.0940 (0.0762–0.1159) *p* < 0.0001
treatment (*k*_0_/*k*_1_ < ratio_thresh_) versus treatment (all)	3.938 (1.985–7.811) *p* < 0.0001	2.119 (1.294–3.47) *p* = 0.0005	15.64 (6.536–37.41) *p* < 0.0001	5.074 (2.653–9.705) *p* < 0.0001	0.5152 (0.3481–0.8458) *p* = 0.0035

^a^Protocol A.

^b^Protocol Z.

^c^Protocol V11a.

^d^Protocol V11a-D.

We performed a similar analysis using the median *k*_1*,*thresh_ value (1.661 × 10^−6^) to show the distinction between the survival estimates (electronic supplementary material, figure S3). The control and treated groups with *k*_1_ smaller than *k*_1*,*thresh_ have better survival estimates than those with larger *k*_1_ values. We also estimated the median survival of the six groups separated using the median *k*_1*,*thresh_ (table 3), the Mantel–Haenszel HR and the *p*-values from the Mantel–Cox log rank test for survival curve comparison (table 4). From these analyses, mice with smaller *k*_1_ survive longer than those with larger *k*_1_, and the HR is smaller than one (*p* < 0.05).

**Table 3.** Summary of median survival of the population separated by median *k*_1*,*thresh_.

median survival (days)	Roland^a^	Zibara^a^	Tan^a^	Volk 2008^a^	Volk 2011a^a^	Volk 2011b^a^	Zibara^b^	Volk 2011a^c^	Volk 2011a^d^	Mollard^a^
control (all)	53	55	58	64	69	68	55	69	69	87
control (*k*_1_ < *k*_1_*_,_*_thresh_)	59.5	84	86	Un^e^	87	Un^e^	84	87	87	92
control (*k*_1_ > *k*_1_*_,_*_thresh_)	35	45	43	41	44	45	45	44	44	72
treatment (all)	Un^e^	63	77.5	Un^e^	69	Un^e^	63	71	Un^e^	Un^e^
treatment (*k*_1_ < *k*_1_*_,_*_thresh_)	Un^e^	Un^e^	Un^e^	Un^e^	88	Un^e^	108	108	Un^e^	Un^e^
treatment (*k*_1_ > *k*_1_*_,_*_thresh_)	55	46	46	47.5	44	82	44	44	78.5	Un^e^

^a^Protocol A.

^b^Protocol Z.

^c^Protocol V11a.

^d^Protocol V11a-D.

^e^Un, undefined. The median survival cannot be estimated if the survival estimation does not reach below 50%.

**Table 4.** Statistics comparing the Kaplan–Meier survival curves of the population separated by median *k*_1*,*thresh_: hazard ratio (95%CI) and log rank test *p*-values.

HR (95% CI) *p*-value	Roland^a^	Zibara^a^	Tan^a^	Volk2008^a^	Volk2011a^a^
treatment (*k*_1_ < *k*_1_*_,_*_thresh_) versus treatment (*k*_1_ > *k*_1_*_,_*_thresh_)	0.0012 (0.0004–0.0041) *p* < 0.0001	0.0904 (0.0456–0.1793) *p* < 0.0001	0.0794 (0.0422–0.1491) *p* < 0.0001	0.0241 (0.0118–0.0493) *p* < 0.0001	0.0138 (0.0063–0.0301) *p* < 0.0001
control (*k*_1_ < *k*_1_*_,_*_thresh_) versus control (*k*_1_ > *k*_1_*_,_*_thresh_)	0.5882 (0.3549–0.9751) *p* < 0.0001	0.0832 (0.0421–0.1643) *p* < 0.0001	0.0809 (0.0430–0.152) *p* < 0.0001	0.0241 (0.0118–0.0492) *p* < 0.0001	0.01376 (0.0063–0.0301) *p* < 0.0001
treatment (*k*_1_ < *k*_1_*_,_*_thresh_) versus control (*k*_1_ < *k*_1_*_,_*_thresh_)	0.134 (0.0805–0.2231) *p* < 0.0001	0.0927 (0.0258–0.3324) *p* = 0.0003	0.0843 (0.0287–0.2471) *p* < 0.0001	0.1775 (0.0682–0.462) *p* = 0.0004	0.9909 (0.5909–1.662) *p* = 0.9724
treatment (*k*_1_ < *k*_1_*_,_*_thresh_) versus treatment (all)	0.5033 (0.2978–0.8505) *p* = 0.0103	0.2096 (0.1054–0.4169) *p* < 0.0001	0.2076 (0.1112–0.3874) *p* < 0.0001	0.2094 (0.1143–0.3839) *p* < 0.0001	0.515 (0.3422–0.7749) *p* = 0.0015
treatment (all) versus control (all)	0.2307 (0.1548–0.3438) *p* < 0.0001	0.6742 (0.4384–1.037) *p* = 0.0726	0.5845 (0.3934–0.8684) *p* = 0.0079	0.6481 (0.4331–0.9699) *p* = 0.0350	0.9959 (0.7038–1.409) *p* = 0.9815
treatment (*k*_1_ > *k*_1_*_,_*_thresh_) versus treatment (all)	30.33 (12.08–76.16) *p* < 0.0001	2.553 (1.538–4.239) *p* = 0.0003	2.759 (1.728–4.405) *p* < 0.0001	5.824 (3.356–10.1) *p* < 0.0001	8.024 (4.379–14.7) *p* < 0.0001

HR (95% CI) *p*-value	Volk2011b^a^	Zibara^b^	Volk2011a^c^	Volk2011a^d^	Mollard^a^
treatment (*k*_1_ < *k*_1_*_,_*_thresh_) versus treatment (*k*_1_ > *k*_1_*_,_*_thresh_)	0.0662 (0.0301–0.1456) *p* < 0.0001	0.0904 (0.0456–0.1793) *p* < 0.0001	0.0138 (0.0063–0.0301) *p* < 0.0001	0.0265 (0.0118–0.0595) *p* < 0.0001	0.1265 (0.0619–0.2588) *p* < 0.0001
control (*k*_1_ < *k*_1_*_,_*_thresh_) versus control (*k*_1_ > *k*_1_*_,_*_thresh_)	0.0254 (0.0126–0.0513) *p* < 0.0001	0.0832 (0.0421–0.1643) *p* < 0.0001	0.0138 (0.0063–0.0301) *p* < 0.0001	0.0138 (0.0063–0.0301) *p* < 0.0001	0.6814 (0.5175–0.8971) *p* = 0.0063
treatment (*k*_1_ < *k*_1_*_,_*_thresh_) versus control (*k*_1_ < *k*_1_*_,_*_thresh_)	0.0960 (0.0410–0.2244) *p* < 0.0001	0.0927 (0.0258–0.3324) *p* = 0.0003	0.5917 (0.3641–0.9618) *p* = 0.0342	0.0636 (0.0329–0.1233) *p* < 0.0001	0.0781 (0.0611–0.0999) *p* < 0.0001
treatment (*k*_1_ < *k*_1_*_,_*_thresh_) versus treatment (all)	0.1959 (0.0899–0.4268) *p* < 0.0001	0.2096 (0.1054–0.4169) *p* < 0.0001	0.5129 (0.3404–0.7728) *p* = 0.0014	0.1783 (0.0858–0.3702) *p* < 0.0001	0.5779 (0.3567–0.9361) *p* = 0.0259
treatment (all) versus control (all)	0.266 (0.1733–0.4083) *p* < 0.0001	0.6742 (0.4384–1.037) *p* = 0.0726	0.7766 (0.5543–1.088) *p* = 0.1416	0.2016 (0.1343–0.3027) *p* < 0.0001	0.0940 (0.0762–0.1159) *p* < 0.0001
treatment (*k*_1_ > *k*_1_*_,_*_thresh_) versus treatment (all)	3.248 (1.77–5.959) *p* = 0.0001	2.168 (1.336–3.516) *p* = 0.0003	8.024 (4.379–14.7) *p* < 0.0001	3.518 (1.913–6.472) *p* < 0.0001	3.444 (1.863–6.365) *p* < 0.0001

^a^Protocol A.

^b^Protocol Z.

^c^Protocol V11a.

^d^Protocol V11a-D.

### 2.3. Alternative treatment strategies to improve survival estimates

We next sought to understand whether alternative treatment protocols can effectively reduce tumour volume for the Zibara and Volk2011a cases, because the baseline protocol did not significantly affect tumour volume. For the Zibara case, we simulated the original treatment protocol used in the experimental study (termed ‘protocol Z’). This protocol starts the 10 mg kg^−1^ biweekly treatment upon tumour engraftment (assuming the initial tumour volume to be 0.004 cm^3^) [36]. The predicted tumour volumes are smaller in the treated group (electronic supplementary material, figure S4A), recapitulating the findings from the published experimental study. The predictions may suggest that, in this case, starting the treatment earlier is more effective in limiting the tumour growth. For mice with *k*_0_/*k*_1_ ratios larger than the median ratio_thresh_, or with *k*_1_ smaller than the median *k*_1*,*thresh_, the HR between the treated and control groups is smaller than one, and the survival curves are significantly different (*p* < 0.0001) (tables 2 and 4).

For Volk2011a, we simulated treatment termed ‘protocol V11a’, which starts the 10 mg kg^−1^ biweekly treatment when the tumour volume reaches 0.5 cm^3^, a start time extracted from the published preclinical study [39]. After 12 weeks, the simulated mean tumour volumes in the treated group are significantly smaller than the control tumours (electronic supplementary material, figure S4B). However, the survival estimates were not significantly different (*p* > 0.05). Again, the treated group with *k*_0_/*k*_1_ ratios larger than the median ratio_thresh_, or with *k*_1_ smaller than the median *k*_1*,*thresh_*,* has a significantly better survival estimate than the opposite group (*p* < 0.0001) (tables 2 and 4). This phenomenon is similar to that observed in the Volk2011a case using protocol A, where the two groups separated according to the *k*_0_/*k*_1_ ratio or *k*_1_ have distinct survival estimates, but there is no significant difference between the treated and control groups.

Finally, we explored whether another treatment protocol could significantly improve the survival estimates for the treated group compared to the control. We simulated protocol V11a-D, where biweekly treatment starts when the tumour volume reaches 0.5 cm^3^, and the drug dosage is doubled to 20 mg kg^−1^. This treatment protocol significantly limits the tumour growth (electronic supplementary material, figure S4C), and the survival curves are significantly better for the treated group compared to the control (*p* < 0.0001). Overall, the treated and control groups have an HR of 0.2016 (95% CI: 0.1343–0.3027) (table 2).

### 2.4. Validation of thresholds using an independent dataset

**Figure 5.**
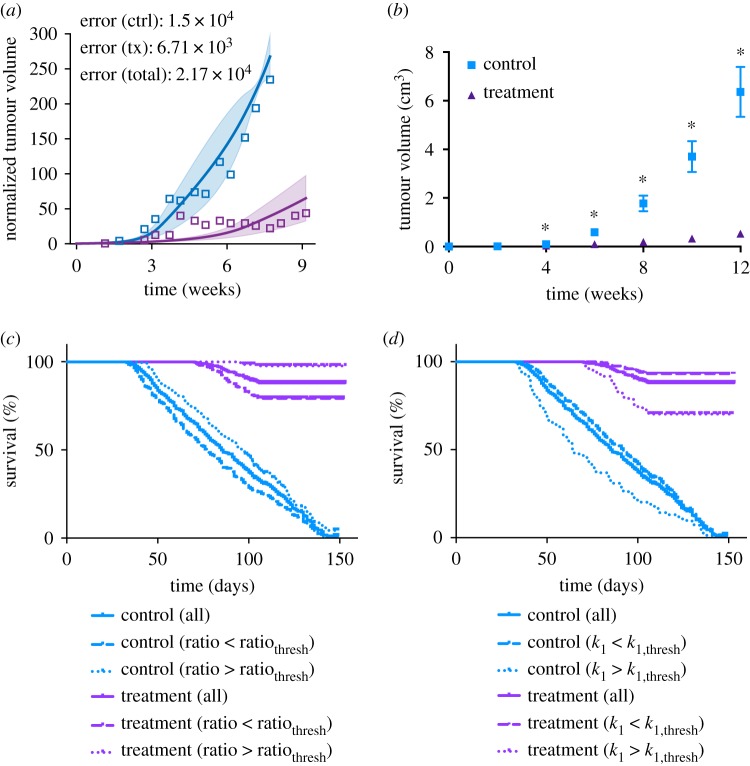
Validation of ratio_thresh_ and *k*_1*,*thresh_ values with an independent set of data from Mollard *et al*. [41]. (*a*) Model fit to control data and validation with treatment data from Mollard *et al*. [41]. The model was fitted to normalized tumour volume, and the tumour growth kinetic parameters were estimated. The model is able to reproduce experimental data in the control group and predict the treatment data. *Line*: mean of best fits*. Shading*: range of standard deviation. *Squares*: experimental data. *Error values*: SSR for mean of the best fits. (*b*) Model-simulated tumour growth of an *in silico* mouse population, with tumour growth kinetic parameters *k*_0_ and *k*_1_ for each simulation randomly varied within the range of their estimated values. The mean and 95% confidence interval at each time point are shown. Asterisks indicate that the difference between the control and treatment group tumour volumes is statistically significant (*p* < 0.05). (*c,d*) Estimated Kaplan–Meier survival curves of the simulated mouse population obtained using the model that was fitted to Mollard data. The population is separated using the median of the range of (*c*) ratio_thresh_ values (13.8693), or (*d*) *k*_1*,*thresh_ values (1.661 × 10^−6^).

To validate the use of the range of ratio_thresh_ and *k*_1*,*thresh_ values that we found, we used a recently published independent set of data that measures tumour growth in mice with MDA-MB-231 xenografts, with or without bevacizumab treatment [41]. First, we fitted the model to the measured tumour volumes without treatment. We obtained 12 sets of estimated parameter values for *k*_0_, *k*_1_ and *Ang*_0_ that allow the model to best fit to the control data. We then validated the fitted model by simulating anti-VEGF treatment and comparing to the experimental measurements. The predicted tumour growth with treatment matches closely to the experimental data (figure 5*a*).

Using the same approach as described above, we generated 400 sets of tumour volumes for an *in silico* mouse population with and without treatment (referred to as ‘Mollard’). To do so, we randomly varied *k*_0_ and *k*_1_ from the ranges of the 12 sets of estimated parameter values from model fitting to the Mollard dataset, with *Ang*_0_ held constant at the median of its estimated values. The simulated tumour volumes for the control and treated groups are shown in figure 5*b*.

We generated the population survival data based on the simulated tumour growth profiles. We tested whether the common range of ratio_thresh_ and *k*_1*,*thresh_ values identified using the six datasets described above are able to separate the population survival data for this validation case (Mollard). For all ratio_thesh_ values within the range, the survival estimate of the treated mice with *k*_0_/*k*_1_ ratios larger than the threshold is better than those with smaller *k*_0_/*k*_1_ ratios. Examples using the median ratio_thresh_ and the median *k*_1*,*thresh_ are shown in figure 5*c,d*. We calculated the HR values, as well as the *p*-value from the Mantel–Cox log rank test among the treated and control groups, separated using the median of the common ratio_thresh_ range (table 2) or the common *k*_1*,*thresh_ range (table 4). Thus, we were able to validate the threshold values.

### 2.5. Tumour growth dynamics among stratified populations

We explored the dynamics of the tumour growth for the groups separated by the threshold values to better understand why the anti-VEGF treatment has differential effects in the simulated mouse populations. As researchers have pointed out, log transformation of tumour growth data provides information on tumour growth rates (given by the slope of the curve) and is more suitable for detecting a transient biological or therapeutic effect [40,42,43]. Therefore, we compared the mean RTV time courses (electronic supplementary material, figure S1) and the mean tumour volume data plotted on the log scale (electronic supplementary material, figure S5) of the groups stratified by the median ratio_thresh_ (13.869) in each case.

**Figure 6.**
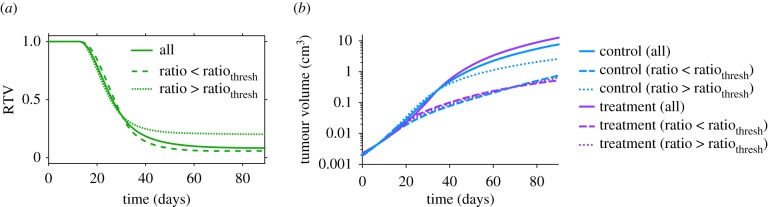
Dynamics of tumour volume. (*a*) Time course of relative tumour volume (RTV) for the Mollard case. The mean RTV for all *in silico* mice and mice with tumours whose *k*_0_*/k*_1_ ratio is smaller or larger than the median ratio_thresh_ (13.8693) are shown. (*b*) Tumour volume data plotted on the log scale for all *in silico* mice and mice separated according to the tumour's *k*_0_*/k*_1_ ratio.

For Roland, Tan and Volk2008, the mean RTV of the group with larger *k*_0_/*k*_1_ ratios (electronic supplementary material, figure S1A,C,D) is initially larger, and then becomes smaller relative to the opposite group. This switch occurs because in the group with larger *k*_0_/*k*_1_ ratios, the difference between the treated and control tumour volumes is smaller at early times, and then becomes larger (electronic supplementary material, figure S5). Meanwhile, the actual tumour volumes for this group are both relatively low. As a result, this group survives longer (figure 4). For the Mollard case, the differences between the treated and control tumour volumes in the group with larger *k*_0_/*k*_1_ ratios are larger (figure 6*b*, dotted curves), giving rise to the larger mean RTV (figure 6*a*). However, the group with larger *k*_0_/*k*_1_ ratios still survives longer because the actual tumour volumes are relatively low (figure 5*c*).

**Figure 7.**
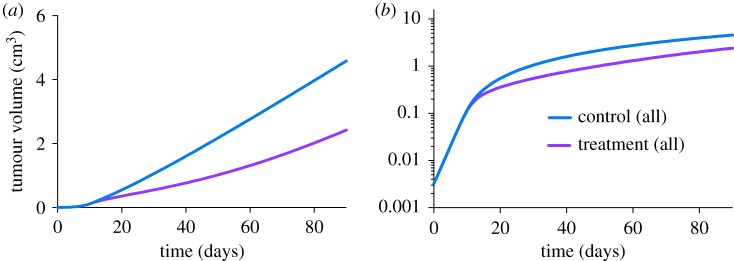
Mean tumour growth. We plot the mean tumour volume for all *in silico* mice in control and treatment groups using the model fitted to Roland data. (*a*) Linear scale and (*b*) log scale.

The tumour volume data plotted on the log scale also reveal that the tumour growth rates of the control and treated groups diverge at different time points. For Roland, the gap between the control and treated groups continually increases when plotted on a linear scale (figure 7*a*). However, the tumour volumes plotted on the log scale show that their growth rates are mostly different during days 14–40. The growth rates become similar during the later stage (after 40 days), as evidenced by the parallel curves on the log scale (figure 7*b*). Therefore, the increasingly large gap between the tumour volumes is a result of early differences in the tumour growth rates. A similar phenomenon is observed for Volk2011b, where the tumour growth rate of the treated group is suppressed transiently at early times but not in the later stage (electronic supplementary material, figure S5). In Zibara, Tan and Volk2008, the growth rates become different between day 30 and day 45, and only gradually become similar towards the end of the simulated time. Overall, analysis of the growth curves plotted on the log scale reveals that the anti-VEGF treatment has differential effects in limiting tumour growth, and the effects occur at different stages for the simulated cases. The treatment effect is predicted to be stronger for the group with *k*_0_/*k*_1_ ratios larger than the median ratio_thresh_.

## 3. Discussion

In this study, we focus on identifying tumour growth kinetic parameters as potential biomarkers for the outcome of anti-VEGF treatment. We developed a computational approach that incorporates model training, simulation of tumour growth within a heterogeneous population, and estimation and analysis of population response.

We simulated anti-VEGF treatment and compared the effect of treatment across tumour-bearing mice generated from our previous fitting to six independent preclinical studies. For most simulated tumours, the anti-VEGF agent significantly reduces tumour volume compared to the control. However, our simulations for Zibara and Volk2011a show that these populations do not respond to the treatment (figure 2*b,e*), which is different from the effect seen experimentally. This difference occurs for two reasons. First, our simulated treatment protocol A is universal across the six cases, and is different from what was used in each of the original six experimental studies. Second, in our simulations, *k*_0_ and *k*_1_ are varied simultaneously and independently of each other, possibly resulting in more variability than what occurs experimentally.

Our study demonstrates that the *k*_0_/*k*_1_ ratio or *k*_1_ alone can be used to stratify the population response with or without anti-VEGF treatment. This finding agrees with our previous finding through PLSR analysis that the ratio is a key predictor of the tumour response to anti-VEGF treatment [26]. Building on that framework, we found that the survival estimate of mice with larger *k*_0_/*k*_1_ ratios or smaller *k*_1_ is better compared to those with smaller ratios or higher *k*_1_. Interestingly, the result for the ratio is the opposite of the conclusion we drew previously (that a larger ratio correlates with a poorer response to treatment). However, in that work, we focused only on whether the final RTV value was low. This highlights the fact that only evaluating the endpoint RTV of the treated and control group and neglecting the actual tumour volume data over time can lead to misinterpretation of the treatment effect. Indeed, researchers have recognized that while most preclinical studies focus on the endpoints of tumour growth, monitoring tumour growth kinetically may be more insightful [42,43].

We found that, in two cases (Volk2011a simulated with protocols A and V11a), no significant difference is observed in the survival estimates between the treated and control groups. However, even for these cases, two populations with significantly different survival estimates can be identified based on their *k*_0_/*k*_1_ ratios (figure 4*b,e*) or *k*_1_ value (electronic supplementary material, figure S3B,E). This indicates that even when the treatment is not effective in reducing tumour volume, there is still a difference in tumour growth dynamics between the two populations stratified based on the tumour's growth kinetic parameters. Thus, we believe that the *k*_0_/*k*_1_ ratio or *k*_1_ may be prognostic biomarkers to stratify populations for their survival estimate without anti-angiogenic treatment. Interestingly, the parameters provide mechanistic insight into tumour growth. In particular, they highlight that slower linear growth (larger ratio or smaller *k*_1_) results in less aggressive overall tumour growth (electronic supplementary material, figure S5) and, therefore, a better survival outcome.

Another interesting aspect is the utility of *k*_1_ to serve as a prognostic biomarker. Although *k*_1_ was not revealed as a strong predictor of the final RTV previously in the PLSR analysis, it is inversely correlated with the *k*_0_/*k*_1_ ratio, and therefore, in our study, it also can be used to stratify the population survival outcome. Here, performing the survival analysis addresses one limitation from our previous PLSR analysis, where we were able to identify which parameters were related to treatment efficacy, but could not identify the specific relationship between the kinetic parameter values and effectiveness of the treatment.

Compared to the mean RTV data, the tumour volume data provide more useful insight into the tumour growth characteristics of the stratified population. In particular, the tumour volume plotted on the log scale more clearly illustrates the source of the differences in the population survival estimates. Specifically, we found that larger *k*_0_/*k*_1_ ratios often yield slower tumour growth in a population, and therefore, lead to a better survival estimate of the population. This conclusion could not be made if we were to only analyse the RTV data. In addition, the tumour volumes plotted on the log scale reveal that the effect of anti-VEGF treatment in tumour growth can be relatively transient or gradual.

Our study uses a predictive computational model of tumour growth. This is a pharmacokinetics–pharmacodynamics model with mechanistic detail that goes beyond what is found in other models. However, in the future, this model can be expanded to address limitations that are not currently accounted for. For example, we do not account for changes in tumour vascularity relative to tumour volume. We assume the vascular volume relative to total tumour volume remains constant, given the lack of robust quantitative data needed to develop a mathematical function describing how tumour vascularization changes over time. In addition, vascular normalization is an important process that has been shown to affect tumour growth and can be regulated by anti-VEGF agents [32]; however, this process is not included in our model. These aspects can be implemented into the model as more quantitative data become available and enable us to characterize the dynamics of vessel normalization. The model can then be further extended to account for other characteristics of tumour progression, including tumour perfusion and metastatic potential. The model can also be adapted to simulate the effect of cytotoxic drugs that target tumour cells, which in turn will affect the tumour volume. Furthermore, the range of threshold values for tumour stratification is constrained by the estimated parameter values from model training to each experimental dataset. It is possible that artefacts from experimental data quantification led to bias in the range of the fitted parameter values. This can be improved when more quantitative experimental data become available for additional model training. We note that the biomarker candidates identified in this study are best used to stratify populations for their survival outcome, whether the mice receive treatment or not, rather than to predict treatment efficacy. This is primarily because the datasets used for model training were tumour volumes measured over several weeks. Our results would be of broader applicability if only pretreatment data were adequate to train the model. We attempted such an approach in previous work [26]; however, the simulated volumes varied widely, preventing us from making conclusive predictions. Despite this perceived limitation, our modelling approach generates hypotheses about potential biomarkers, and spurs on experimental validation to ensure the utility of the biomarkers identified.

Our study demonstrates a time- and cost-effective way to generate large *in silico* mouse populations, predict anti-VEGF treatment outcome and stratify the populations. This approach provides useful information that could facilitate efficient experimental design, such as predicting the effect of different treatment protocols (varying the dosage and the timing of the injections). Additionally, our modelling approach can be adapted for analysis of the patient treatment outcome in clinical studies. With data from a small patient population, we can develop a patient-specific model and generate a larger *in silico* population. Analysis of the simulated tumour growth and survival data can be used to identify biomarkers that predict responders versus non-responders to anti-VEGF treatment, stratify the predicted population survival and test the response to various treatment schedules.

## 4. Conclusion

We examined tumour growth kinetic parameters as potential biomarkers of anti-angiogenic treatment outcome. Using a computational model that simulates VEGF-dependent tumour growth in tumour-bearing mice, we generated an *in silico* mouse population and related the kinetic parameters that characterize tumour growth to the response to anti-VEGF treatment. We found that the ratio between two tumour growth kinetic parameters, *k*_0_ and *k*_1_, as well as *k*_1_ alone, can be prognostic biomarkers and that the simulated treatment protocol may have a better outcome for mice whose tumours have smaller linear growth rates. In fact, we found ranges of threshold values for the *k*_0_/*k*_1_ ratio and *k*_1_ that distinguish tumours' response to the anti-VEGF treatment. This study demonstrates an approach for identifying tumour growth kinetic parameters as potential biomarkers, and this model framework can be adapted to predict the efficacy of other anti-angiogenic strategies.

## 5. Methods

### 5.1. Computational model

We use our previously calibrated and validated model of VEGF binding and distribution in a tumour-bearing mouse [26]. Electronic supplementary material, file S1, contains the full model description. Briefly, the model comprises three compartments representing the whole mouse (figure 1): normal tissue, blood and tumour tissue. We include human VEGF isoforms (VEGF_121_ and VEGF_165_) secreted by tumour cells, as well as mouse isoforms (VEGF_120_ and VEGF_164_) secreted by endothelial cells and muscle fibres. The model includes cell surface VEGF receptors, VEGFR1 and VEGFR2 and soluble VEGFR1 (sVEGFR1). We include neuropillin co-receptors (NRP1 and NRP2) that bind VEGF directly and also form tertiary complexes with the VEGFRs. The protease inhibitor α-2-macroglobulin binds VEGF in blood plasma. We consider the luminal and abluminal endothelial surfaces at the interface between the blood and each tissue compartment. The VEGF isoforms and sVEGFR1 are transported between compartments via transendothelial macromolecular permeability and lymphatic flow. Additionally, species are removed via clearance.

VEGF binding to its receptors on endothelial cells promotes intracellular signalling that mediates angiogenesis. Thus, we explicitly account for VEGF-mediated tumour growth by incorporating the concentration of ligated receptors localized on tumour endothelial cells into the tumour volume equation (figure 1). We simulate anti-VEGF treatment as intravenous injections lasting for 1 min by adding a net rate of secretion of the drug (bevacizumab) directly into the blood compartment.

### 5.2. Numerical implementation

Model equations were implemented in Matlab using the SimBiology toolbox. The model is provided as the SimBiology project file, SBML, Matlab m-file and full list of equations (electronic supplementary material, file S2). Parameter fitting was performed using the *lsqnonlin* function in Matlab. Kaplan–Meier survival estimation was performed using the *kmplot* function in Matlab, and GraphPad Prism was used for statistical survival analyses.

### 5.3. Simulation of *in silico* mouse population

We previously fitted the model to six independent control datasets to estimate the growth kinetic parameters (*k*_0_, *k*_1_ and *Ang*_0_). The parameter *ψ* was held constant, as it was not shown to significantly influence tumour growth, compared to the other parameters. The model-predicted tumour growth curves match closely to the experimental data (fitting error range: 0.0405–0.1833).

Here, we generated 400 sets of values for *k*_0_ and *k*_1_, randomly selected from a uniform distribution within the range of the best fit parameter sets from our previous study (electronic supplementary material, table S1). The *Ang*_0_ value is set to be the median of the best fits in each case. These sets were used to calculate tumour growth with or without anti-VEGF treatment, simulating a population of mice for each dataset. To keep tumour growth profiles realistic, tumours that do not reach 0.1 cm^3^ within 10 days upon tumour engraftment (assuming an initial tumour volume of 0.004 cm^3^) were excluded from the analyses.

We simulated anti-VEGF treatment for each dataset. Treatment protocol A is simulated universally across the six cases. In this protocol, weekly treatment starts when the tumour volume reached 0.1 cm^3^, as the switch where angiogenesis is more strongly promoted occurs when the tumour reaches 1–2 mm in diameter. The treatment dosage is 10 mg kg^−1^. The model was simulated for 12 weeks after treatment started. We also simulated alternative treatment protocols: Z denotes biweekly treatment at a dosage of 10 mg kg^−1^ starting when the tumour volume is 0.004 cm^3^; V11a denotes biweekly treatment (twice a week) at a dosage of 10 mg kg^−1^, starting when the tumour volume is 0.5 cm^3^; and V11a-D denotes biweekly treatment at a dosage of 20 mg kg^−1^, starting when the tumour volume is 0.5 cm^3^. Information for all treatment protocols is given in electronic supplementary material, table S3.

### 5.4. Relative tumour volume

Based on the model-generated tumour growth data, the RTV is calculated at any simulated time point as follows:
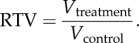
An RTV value less than one indicates that the treated tumour volume is smaller than the control.

### 5.5. Kaplan–Meier survival estimation

We applied time-to-event analysis to determine the survival of each mouse population [34]. An *in silico* mouse is recorded as ‘sacrificed’ when its tumour reaches 2 cm^3^ within the simulated time. Alternatively, a mouse is recorded as ‘censored’ at a particular time point, *t*, if its tumour volume simulation remains below 2 cm^3^ but ends before time *t*. All other mice are retained in the study and recorded as ‘alive’. Survival curves were estimated by the Kaplan–Meier method using the *kmplot* function in Matlab [44], and compared using the Mantel–Cox log rank test and the Mantel–Haenszel HR in GraphPad Prism.

The HR compares the rate of death in two groups, with the assumption that the population HR is consistent over time. It is calculated using the Mantel–Haneszel approach, which is more accurate than the log rank approach [45]. As an example, an HR of 0.5 between two groups means that the death rate of the first group is half of that of the second group.

### 5.6. Determination of threshold values

To determine threshold values for the *k*_0_/*k*_1_ ratio, we ordered the simulated mouse tumour volume data for each of the six populations according to the *k*_0_/*k*_1_ ratio. Then, we systematically tested each *k*_0_/*k*_1_ ratio (called ‘ratio_thresh_’) value to see if there is a significant difference between the survival estimates for the mice with the *k*_0_/*k*_1_ ratio above and below ‘ratio_thresh_’ in the log rank test (*p* < 0.05). We performed a similar analysis for *k*_0_ and *k*_1_ individually to determine any *k*_0*,*thresh_ and *k*_1*,*thresh_ values.

### 5.7. Validation of the predicted biomarker

Upon identifying a potential predictive biomarker for the efficacy of anti-VEGF treatment, we validated our findings using an independent set of data that was not used to determine the range of the threshold values. To do so, we fitted the control tumour growth for the independent dataset and generated an *in silico* mouse population based on the fitted parameters.

#### 5.7.1. Data extraction

For threshold validation, data from the published *in vivo* experimental study of MDA-MB-231 xenograft tumour growth in mice by Mollard *et al*. were used for parameter estimation and validation [41]. Experimental data were extracted using the WebPlotDigitizer program [46] and are shown in electronic supplementary material, table S1.

#### 5.7.2. Parameter estimation

We trained the model to fit the control tumour growth dataset from [41] using the same approach as described in our previous work [26]. The values of tumour growth parameters *k*_0_, *k*_1_ and *Ang*_0_ were estimated. In their study, Mollard and co-workers only reported the tumour volumes relative to day 8. However, the absolute tumour volumes are needed to determine how the tumour interstitial volume varies as a function of the total tumour volume. Therefore, we compared the RTV at each time point in the work by Mollard and co-workers to that of all the available control datasets (electronic supplementary material, figure S6). We then chose to use the interstitial volume equation from the Zibara data, given that the RTV closely matches that of the data in Mollard. Finally, we fitted our tumour growth model to the Mollard control dataset.

Fitting was performed using the *lsqnonlin* function in Matlab to minimize the sum of squared residuals (SSR):
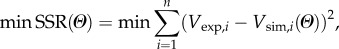
where *V*_exp,*I*_ is the *i*th experimental data point of tumour volume, *V*_sim*,I*_ is the *i*th simulated volume at the corresponding time point and *n* is the total number of experimental data points. The minimization is subject to *Θ*, the set of upper and lower bounds on each of the free parameters.

The bounds for each parameter spanned at least two orders of magnitude: 10^−8^ to 10^−2^ for *k*_0_ and *k*_1_ and 10^−16^ to 10^−14^ for *Ang*_0_. After fitting to the control data, we validated the estimated parameters with data not used in the fitting for model validation. Specifically, we applied the fitted model to simulate anti-angiogenic treatment (bevacizumab) and compared to the experimental measurements for the treatment case. We simulated the dosing regimen used by Mollard *et al*.: three cycles of weekly intravenous injections lasting for 1 min starting from day 5. We used the combined SSR for the RTV between model prediction and the experimental data (both control and treatment) to identify the optimal parameters. Twelve parameter sets with the smallest errors were taken to be the ‘best’ sets (electronic supplementary material, table S1) and the ranges of the estimated parameter values were used for subsequent model simulations (electronic supplementary material, table S2).

We extracted the absolute tumour volume at day 8 from previously reported data from Mollard and co-workers [47] to determine the survival estimates for a mouse population simulated based on the fitted growth kinetics parameter values.

## Supplementary Material

File S1. Description of three-compartment model

## Supplementary Material

File S2. Compressed file containing the computational model

## Supplementary Material

File S3. Supplemental tables

## Supplementary Material

File S4. Supplemental figures

## Data accessibility

All data analysed during this study, including the model used for data generation, are included in this published article and its electronic supplementary material information files.

## References

[1] Carmeliet P, Jain RK 2011 Molecular mechanisms and clinical applications of angiogenesis. Nature 473, 298–307. (10.1038/nature10144) 21593862 PMC4049445

[2] Belal A, Maha A, Morgan T, DeRemer DL, Somanath PR 2012 Antiangiogenic therapy for cancer: an update. Pharmacotherapy 32, 1095–1111. (10.1002/phar.1147) 23208836 PMC3555403

[3] FDA Approval for Bevacizumab. 2018 National Cancer Institute https://www.cancer.gov/about-cancer/treatment/drugs/fda-bevacizumab (accessed 2 Apr 2018).

[4] Montero AJ, Escobar M, Lopes G, Glück S, Vogel C 2012 Bevacizumab in the treatment of metastatic breast cancer: friend or foe? Curr. Oncol. Rep. 14, 1–11. (10.1007/s11912-011-0202-z) 22012632 PMC3266439

[5] Lambrechts D, Lenz H-J, de Haas S, Carmeliet P, Scherer SJ 2013 Markers of response for the antiangiogenic agent bevacizumab. J. Clin. Oncol. 31, 1219–1230. (10.1200/JCO.2012.46.2762) 23401453

[6] Rubovszky G, Horváth Z 2017 Recent advances in the neoadjuvant treatment of breast cancer. J. Breast Cancer 20, 119–131. (10.4048/jbc.2017.20.2.119) 28690648 PMC5500395

[7] Carey LA et al. 2016 Molecular heterogeneity and response to neoadjuvant human epidermal growth factor receptor 2 targeting in calgb 40601, a randomized phase iii trial of paclitaxel plus trastuzumab with or without lapatinib. J. Clin. Oncol. 34, 542–549. (10.1200/JCO.2015.62.1268) 26527775 PMC4980567

[8] Untch M et al. 2012 Lapatinib versus trastuzumab in combination with neoadjuvant anthracycline-taxane-based chemotherapy (GeparQuinto, GBG 44): a randomised phase 3 trial. Lancet Oncol. 13, 135–144. (10.1016/S1470-2045(11)70397-7) 22257523

[9] Bear HD et al. 2015 Neoadjuvant plus adjuvant bevacizumab in early breast cancer (NSABP B-40 [NRG Oncology]): secondary outcomes of a phase 3, randomised controlled trial. Lancet Oncol. 16, 1037–1048. (10.1016/S1470-2045(15)00041-8) 26272770 PMC4624323

[10] Earl HM et al. 2015 Efficacy of neoadjuvant bevacizumab added to docetaxel followed by fluorouracil, epirubicin, and cyclophosphamide, for women with HER2-negative early breast cancer (ARTemis): an open-label, randomised, phase 3 trial. Lancet Oncol. 16, 656–666. (10.1016/S1470-2045(15)70137-3) 25975632

[11] Nahleh ZA et al. 2016 SWOG S0800 (NCI CDR0000636131): addition of bevacizumab to neoadjuvant nab-paclitaxel with dose-dense doxorubicin and cyclophosphamide improves pathologic complete response (pCR) rates in inflammatory or locally advanced breast cancer. Breast Cancer Res. Treat. 158, 485–495. (10.1007/s10549-016-3889-6) 27393622 PMC4963434

[12] Rugo HS et al. 2016 Adaptive randomization of veliparib–carboplatin treatment in breast cancer. New Engl. J. Med. 375, 23–34. (10.1056/NEJMoa1513749) 27406347 PMC5259561

[13] Wehland M, Bauer J, Magnusson NE, Infanger M, Grimm D 2013 Biomarkers for anti-angiogenic therapy in cancer. Int. J. Mol. Sci. 14, 9338–9364. (10.3390/ijms14059338) 23629668 PMC3676786

[14] Burstein HJ et al. 2008 Vegf as a marker for outcome among advanced breast cancer patients receiving anti-vegf therapy with bevacizumab and vinorelbine chemotherapy. Clin. Cancer Res. 14, 7871–7877. (10.1158/1078-0432.CCR-08-0593) 19047116

[15] Jain RK, Duda DG, Willett CG, Sahani DV, Zhu AX, Loeffler JS, Batchelor TT, Sorensen AG 2009 Biomarkers of response and resistance to antiangiogenic therapy. Nat. Rev. Clin. Oncol. 6, 327–338. (10.1038/nrclinonc.2009.63) 19483739 PMC3057433

[16] Willett CG et al. 2004 Direct evidence that the VEGF-specific antibody bevacizumab has antivascular effects in human rectal cancer. Nat. Med. 10, 145–147. (10.1038/nm988) 14745444 PMC2693485

[17] Willett CG et al. 2005 Surrogate markers for antiangiogenic therapy and dose-limiting toxicities for bevacizumab with radiation and chemotherapy: continued experience of a phase I trial in rectal cancer patients. J. Clin. Oncol. 23, 8136–8139. (10.1200/JCO.2005.02.5635) 16258121

[18] Willett CG et al. 2009 Efficacy, safety, and biomarkers of neoadjuvant bevacizumab, radiation therapy, and fluorouracil in rectal cancer: a multidisciplinary phase II study. J. Clin. Oncol. 27, 3020–3026. (10.1200/JCO.2008.21.1771) 19470921 PMC2702234

[19] Zhu AX et al. 2009 Efficacy, safety, and potential biomarkers of sunitinib monotherapy in advanced hepatocellular carcinoma: a phase ii study. J. Clin. Oncol. 27, 3027–3035. (10.1200/JCO.2008.20.9908) 19470923 PMC2702235

[20] Batchelor TT et al. 2010 Phase II study of cediranib, an oral pan-vascular endothelial growth factor receptor tyrosine kinase inhibitor, in patients with recurrent glioblastoma. J. Clin. Oncol. 28, 2817–2823. (10.1200/JCO.2009.26.3988) 20458050 PMC2903316

[21] Lee JH, Lee HY, Ahn M-J, Park K, Ahn JS, Sun J-M, Lee KS 2016 Volume-based growth tumor kinetics as a prognostic biomarker for patients with EGFR mutant lung adenocarcinoma undergoing EGFR tyrosine kinase inhibitor therapy: a case control study. Cancer Imaging 16, 5. (10.1186/s40644-016-0063-7) PMC4794857 26984681

[22] Seyal AR et al. 2015 Performance of tumor growth kinetics as an imaging biomarker for response assessment in colorectal liver metastases: correlation with FDG PET. Abdom. Imaging 40, 3043–3051. (10.1007/s00261-015-0546-1) 26353898

[23] Sharouni S E, Kal H, Battermann J 2003 Accelerated regrowth of non-small-cell lung tumours after induction chemotherapy. Br. J. Cancer 89, 2184–2189. (10.1038/sj.bjc.6601418) 14676792 PMC2395273

[24] Stein WD, Yang J, Bates SE, Fojo T 2008 Bevacizumab reduces the growth rate constants of renal carcinomas: a novel algorithm suggests early discontinuation of bevacizumab resulted in a lack of survival advantage. Oncologist 13, 1055–1062. (10.1634/theoncologist.2008-0016) 18827177 PMC3306833

[25] Rezai P, Yaghmai V, Tochetto S, Galizia M, Miller F, Mulcahy MF, Small W 2011 Change in the growth rate of localized pancreatic adenocarcinoma in response to gemcitabine, bevacizumab, and radiation therapy on MDCT. Int. J. Radiat. Oncol. Biol. Phys. 81, 452–459. (10.1016/j.ijrobp.2010.05.060) 21570199

[26] Gaddy TD, Wu Q, Arnheim AD, Finley SD 2017 Mechanistic modeling quantifies the influence of tumor growth kinetics on the response to anti-angiogenic treatment. PLoS Comput. Biol. 13, e1005874 (10.1371/journal.pcbi.1005874) 29267273 PMC5739350

[27] Martin EC, Aarons L, Yates JWT 2016 Accounting for dropout in xenografted tumour efficacy studies: integrated endpoint analysis, reduced bias and better use of animals. Cancer Chemother. Pharmacol. 78, 131–141. (10.1007/s00280-016-3059-x) 27220867 PMC4921113

[28] Claret L, Bruno R 2014 Assessment of tumor growth inhibition metrics to predict overall survival. Clin. Pharmacol. Ther. 96, 135–137. (10.1038/clpt.2014.112) 25056390

[29] Altrock PM, Liu LL, Michor F 2015 The mathematics of cancer: integrating quantitative models. Nat. Rev. Cancer 15, 730–745. (10.1038/nrc4029) 26597528

[30] Masoudi-Nejad A, Wang E 2015 Cancer modeling and network biology: accelerating toward personalized medicine. Semin. Cancer Biol. 30, 1–3. (10.1016/j.semcancer.2014.06.005) 24969134

[31] Yankeelov TE et al. 2016 Multi-scale modeling in clinical oncology: opportunities and barriers to success. Ann. Biomed. Eng. 44, 2626–2641. (10.1007/s10439-016-1691-6) 27384942 PMC4983505

[32] Jain RK 2013 Normalizing tumor microenvironment to treat cancer: bench to bedside to biomarkers. J. Clin. Oncol. 31, 2205–2218. (10.1200/JCO.2012.46.3653) 23669226 PMC3731977

[33] Jubb AM et al. 2011 Impact of exploratory biomarkers on the treatment effect of bevacizumab in metastatic breast cancer. Clin. Cancer Res. 17, 372–381. (10.1158/1078-0432.CCR-10-1791) 21224365 PMC3023787

[34] Bender BC, Schindler E, Friberg LE 2015 Population pharmacokinetic–pharmacodynamic modelling in oncology: a tool for predicting clinical response. Br. J. Clin. Pharmacol. 79, 56–71. (10.1111/bcp.12258) 24134068 PMC4294077

[35] Roland CL, Dineen SP, Lynn KD, Sullivan LA, Dellinger MT, Sadegh L, Sullivan JP, Shames DS, Brekken RA 2009 Inhibition of vascular endothelial growth factor reduces angiogenesis and modulates immune cell infiltration of orthotopic breast cancer xenografts. Mol. Cancer Ther. 8, 1761–1771. (10.1158/1535-7163.MCT-09-0280) 19567820

[36] Zibara K et al. 2015 Anti-angiogenesis therapy and gap junction inhibition reduce MDA-MB-231 breast cancer cell invasion and metastasis *in vitro* and *in vivo*. Sci. Rep. 5, 12598 (10.1038/srep12598.10.1038/srep12598) 26218768 PMC4517444

[37] Tan G et al. 2014 Combination therapy of oncolytic herpes simplex virus HF10 and bevacizumab against experimental model of human breast carcinoma xenograft. Int. J. Cancer 136, 1718–1730. (10.1002/ijc.29163) 25156870

[38] Volk LD et al. 2008 Nab-paclitaxel efficacy in the orthotopic model of human breast cancer is significantly enhanced by concurrent anti-vascular endothelial growth factor A therapy. Neoplasia 10, 613–623. (10.1593/neo.08302) 18516298 PMC2386546

[39] Volk LD, Flister MJ, Chihade D, Desai N, Trieu V, Ran S 2011 Synergy of nab-paclitaxel and bevacizumab in eradicating large orthotopic breast tumors and preexisting metastases. Neoplasia 13, 327–338. (10.1593/neo.101490) 21472137 PMC3071081

[40] Hather G et al. 2014 Growth rate analysis and efficient experimental design for tumor xenograft studies. Cancer Inform. 13(Suppl. 4), 65–72. (10.4137/CIN.S13974) 25574127 PMC4264612

[41] Mollard S, Ciccolini J, Imbs D-C, Cheikh RE, Barbolosi D, Benzekry S 2017 Model driven optimization of antiangiogenics + cytotoxics combination: application to breast cancer mice treated with bevacizumab + paclitaxel doublet leads to reduced tumor growth and fewer metastasis. Oncotarget 8, 23 087–23 098. (doi:10.18632/oncotarget.15484) 10.18632/oncotarget.15484 PMC5410287 28416742

[42] Gengenbacher N, Singhal M, Augustin HG 2017 Preclinical mouse solid tumour models: status quo, challenges and perspectives. Nat. Rev. Cancer 17, 751–765. (10.1038/nrc.2017.92) 29077691

[43] Nasarre P et al. 2009 Host-derived angiopoietin-2 affects early stages of tumor development and vessel maturation but is dispensable for later stages of tumor growth. Cancer Res. 69, 1324–1333. (10.1158/0008-5472.CAN-08-3030) 19208839 PMC3514474

[44] KMPlot - File Exchange - MATLAB Central. 2018 http://www.mathworks.com/matlabcentral/fileexchange/22293-kmplot (accessed 2 Apr 2018).

[45] GraphPad Statistics Guide. 2018 See https://www.graphpad.com/guides/prism/7/statistics/index.htm?stat_howto_survival.htm (accessed 2 Apr 2018).

[46] WebPlotDigitizer. Extract data from plots, images, and maps. See https://automeris.io/WebPlotDigitizer/ (accessed 2 Apr 2018).

[47] Mollard S, Fanciullino R, Giacometti S, Serdjebi C, Benzekry S, Ciccolini J 2016 *In vivo* bioiluminescence tomography for monitoring breast tumor growth and metastatic spreading: comparative study and mathematical modeling. Sci. Rep. 6, 36173 (10.1038/srep36173) 27812027 PMC5095884

