# Supplementary material for: In silico mouse study identifies tumour growth kinetics as biomarkers for the outcome of anti-angiogenic treatment
Source: J R Soc Interface. 2018 Aug 22;15(145):20180243. doi: 10.1098/rsif.2018.0243 (PMC6127173; doi:10.1098/rsif.2018.0243)
Supplement: File S1. Description of three-compartment model [file rsif20180243supp1.pdf]

Article Title: ***In silico* mouse study identifies tumor growth kinetics as biomarkers for the outcome of anti-angiogenic treatment**

Authors: Qianhui Wu Alyssa D. Arnheim, and Stacey D. Finley

Supplemental File S1 – Detailed description of computational model

Summarized from [1].

Overview

The model is comprised of three compartments representing the whole mouse (**Figure 1**): normal tissue, blood, and tumor tissue. We include human VEGF isoforms ($VEGF_{121}$ and $VEGF_{165}$) secreted by tumor cells and mouse isoforms ($VEGF_{120}$ and $VEGF_{164}$) secreted by endothelial cells and muscle fibers. The model includes the cell surface VEGF receptors, VEGFR1 and VEGFR2, and the soluble form of VEGFR1 (sVEGFR1). We also include co-receptors called neuropilins (NRP1 and NRP2) that bind VEGF directly and also form tertiary complexes with the VEGFRs. The protease inhibitor α-2-macroglobulin binds VEGF in blood plasma. The VEGF isoforms and sVEGFR1 can be transported between compartments via transendothelial macromolecular permeability and lymphatic flow. Additionally, species are removed from the body via clearance. We consider both the luminal and abluminal endothelial surfaces at the interface between the blood and each tissue compartment. The luminal endothelial cell surface faces blood plasma, and the abluminal surface (outside of blood plasma) faces the tissue interstitium.

Parameters

Geometry (23 parameters). The geometric parameters for the tumor compartment are summarized in **Table S1.1** The tumor cell diameter is assumed to be that of MCF-7 breast tumor cells, 12 μm [2]. Assuming tumor cells are dodecahedral, rather than exactly spherical, we set the tumor cell volume and surface area to be 497 μm^3 and 452 μm^2, respectively. Based on the average luminal diameter of capillaries in growing MCF-7 xenografts, 13.94 μm [3–5], an endothelial cell thickness of 0.5 μm, and the relationship between total perimeter and total cross-sectional area in breast cancer capillaries [6, 7], we estimate the capillary perimeter to be 57.7 μm.

We take the extracellular fluid volume fraction in breast tumor xenografts to be 45%, based on a range of measurements, 33% - 76% [2, 8]. This volume fraction is divided into interstitial space

and intravascular space. Using the capillary dimensions described above and an intravascular volume of 10% [9–11], the capillary density is calculated to be 655 capillaries/mm^2. Based on a cell thickness of 0.5 μm, the volume occupied by the endothelial cells of the microvessels is 1.5%. Cancer cells occupy the remaining tissue volume of 53.5%. The volume fractions of microvessels and tumor cells are then used to calculate the total surface area of all vessels and tumor cells per unit volume of tissue: 378 cm^2 endothelial cell surface / cm^3 tissue and 2939 cm^2 tumor cell surface / cm^3 tissue.

The interstitial space is composed of extracellular matrix (ECM), and basement membranes associated with the microvessels (endothelial basement membrane, EBM) and tumor cells (parenchymal basement membrane, PBM). The thickness of the basement membranes is assumed to be 50 nm and 30 nm, for the EBM and PBM, respectively, yielding volume fractions of 0.0081 and 0.0015 cm^3 / cm^3 tissue. The remaining volume of the interstitial space is the ECM volume (34.04%).

Each region of the interstitial space is represented as a porous medium that contains a solid fraction composed primarily of collagen that is unavailable to VEGF, and a fluid fraction that is accessible to VEGF. The size of the pores further limits the volume available for VEGF to diffuse. Therefore, the available volume in the ECM and basement membranes is calculated as the product of the volume, fluid fraction, and partition coefficient. The fluid fraction is the non-collagen fraction and is calculated by using the total collagen content in interstitial space. Given limited data for this measurement, we used 5%, the same value as in our previous models [12–15]. The ratio of basement membrane collagen to total body collagen is assumed to be 0.3, which yields 0.0482 for the ratio of ECM collagen to total body collagen. The fluid fractions are then 0.7 for the basement membranes and 0.9318 for the ECM. The partition coefficient is the ratio of available fluid volume to interstitial fluid volume. We take 0.9 for the partition coefficient for the EBM [16], and the same value is used for the ECM and PBM, as it is difficult to distinguish basement membranes and the ECM [17]. The available fluid volume for the ECM, EBM, and PBM are therefore 0.2916, 9.720×10^{-4}, and 5.082×10^{-3} cm^3 / cm^3 tissue, respectively.

Initial concentrations (32 parameters). Receptor densities and ECM binding site densities are listed in **Table S1.2**. VEGFR1, VEGFR2, and NRP1 on the luminal and abluminal surfaces of diseased endothelial cell surfaces and on tumor cells are based on quantitative flow cytometry

measurements in endothelial cells isolated from tumor tissue, as described in [14]. We assume NRP2 surface concentration on tumor cells at the same level as NRP1. The initial concentrations of all other species are zero.

Kinetic parameters (40 parameters). The kinetic rates for VEGF binding to and dissociating from receptors, co-receptors, and glycosaminoglycan (GAG) chains in the ECM and basement membranes are the same as in our previous papers, based on experimental data [12–14, 18] are given in **Table S1.3**. We use experimental data from [19] for the on and off rates of VEGF binding to the anti-VEGF agent, bevacizumab.

Intercompartmental transport (8 parameters). Transport parameters for VEGF, anti-VEGF and the VEGF/anti-VEGF complex are listed in **Table S1.4**. Parameters that govern transport between the normal and blood compartments are the same as in our previous models [15, 18].

Secretion and clearance rates of soluble species (26 parameters). Tumor cells secrete VEGF into the tumor interstitium at a ratio of 50:50 for $VEGF_{121}$:$VEGF_{165}$, based on experimental quantification of mRNA isoform expression levels [20–24]. Here, we also consider VEGF secretion by EC. We set the secretion ratio of $VEGF_{120}$:$VEGF_{164}$ by EC to be 10:90, similar to the isoform ratio in muscle tissue, since to our knowledge, this ratio has not been determined experimentally. Additionally, we assume normal and tumor EC secrete the same amount of VEGF; tumor EC are a small fraction of the total EC in the body, thus this assumption should not affect VEGF distribution. In our previous work [15], we fit the rates of VEGF secretion by muscle fibers, EC, and tumor cells by parameter optimization, fitting to experimental data from Rudge and coworkers [25]. These fitted values are used in the current model.

The model also includes soluble factors sVEGFR1 and a2M. Endothelial cells are a source of sVEGFR1; therefore, sVEGFR1 is secreted in all three compartments. Endothelial cells also secrete a2M; however, due to its large size, a2M is not transported via transendothelial macromolecular permeability and is confined to the blood compartment. The rates of secretion of sVEGFR1 and a2M are given in **Table S1.4** (below).

Molecular species are removed from the system via two mechanisms: plasma clearance and proteolytic degradation. The values of these parameters are in **Table S1.4**. For the normal endothelium, the permeability to sVEGFR1 and VEGF/sVEGFR1 is calculated using an

empirical relation between the Stokes-Einstein radius, a_E, and molecular weight ($a_E = 0.483\times (MW)^{0.386}$) , the corresponding theoretical macromolecular permeability-surface area product, *PS* [26], and the capillary surface area, *S*. Taking microvascular permeability as *PS/S*, and the calculated value is on the order of 10^{-8} cm/s, between the normal and blood compartments. Since tumor vasculature is more permeable than normal microvessels [27], we assume that the microvascular permeability between the tumor and blood is an order of magnitude higher than permeability between normal and blood for both VEGF and the anti-VEGF or complex. Therefore, the permeability to VEGF is 4×10^{-7} cm/s and 3×10^{-7} cm/s for the anti-VEGF and VEGF/anti-VEGF complex. The permeability to sVEGFR1 and VEGF-bound to sVEGFR1 is 1.5×10^{-7} cm/s.

Change in relative volume of the interstitial space

The relative interstitial space decreases as the tumor volume increases and the blood compartment remains constant. A recent study by Christensen and colleagues published data of concentration of cancer cells in MDA-MB-231 E2-Crimson expressing tumors (cancer cells/mm^3 tumor) [28]. Informed by this data, we calculated the total tumor cell (tumor compartment) volume at each time point for the six experimental datasets, assuming an average tumor cell volume of 905 μm^3. The remaining volume (total volume – (blood compartment + tumor compartment)) is calculated as the interstitial space volume. We then individually fit to the relative interstitial space volume of each dataset with an exponential decay function. The equation for how the relative volume of the interstitial space changes as a function of the total tumor volume is unique for each of the datasets (**Table S1.5**).

Dynamic volume of the tumor compartment

Tumor growth is given by an adapted Gompertz model focusing on the exponential and linear phases of the tumor growth, as previously described [29, 30]. We implemented the model such that the volume of the tumor compartment is also dependent on the “angiogenic signal” (*Ang*) produced when VEGF binds to its receptors on endothelial cells in the tumor. This introduces an explicit relationship between the pro-angiogenic VEGF and tumor growth.

The differential equation for the tumor volume is:

$$\frac{dV(t)}{dt} = \frac{k_0 * V(t)}{\left[1+\left(\frac{k_0}{k_1} * V(t)\right)^{\psi}\right]^{\frac{1}{\psi}}} \cdot \left(1 - \frac{Ang_0 - Ang(t)}{Ang_0}\right) \tag{1a}$$

We note that equation (1a) simplifies to:

$$\frac{dV(t)}{dt} = \frac{k_0 * V(t)}{\left[1+\left(\frac{k_0}{k_1} * V(t)\right)^{\psi}\right]^{\frac{1}{\psi}}} \cdot \left(\frac{Ang(t)}{Ang_0}\right) \tag{1b}$$

Here, $V(t)$ is the tumor volume in cm^3 at time t, k_0 and k_1 are parameters describing the rate of exponential and linear growth, respectively. The units of k_0 and k_1 are s^{-1} and cm^3 tissue/s, respectively. The ψ parameter represents the transition from exponential to linear tumor growth and is unitless. The Ang_0 parameter represents the basal angiogenic signal (at time $t = 0$), and $Ang(t)$ is the angiogenic signal at time t. The value of Ang at any time is calculated as the total concentration of pro-angiogenic VEGF-receptor complexes on tumor endothelial cells. This includes VEGFR1 and VEGFR2 bound to either mouse or human VEGF isoforms, with or without the NRP1 co-receptor. Thus, $Ang(t)$ and Ang_0 have units of concentration (mol/cm^3 tissue).

Supplementary Tables

Table S1.1. Geometric parameters

	**Value**	**Units**	**Reference**
**Cancer cells**			
Tumor cell external diameter	12	μm	[2]
Volume of one cell	905	μm^3	Calculated, see text
Surface area of one cell	497	μm^2	Calculated, see text
**Microvessels**			
Average luminal diameter	13.9	μm	[3]
Endothelial cell thickness	0.5	μm	Based on normal microvessels [31]
Average external diameter	14.9	μm	Calculated, see text
Cross sectional area of one vessel	175.3	μm^2	Calculated, see text
Perimeter of one vessel	57.7	μm	Calculated, see text
Capillary density	655	capillaries/mm^2	Calculated, see text
**Volume fractions**			
Interstitial space	35.0%	cm^2/cm^3 tissue	Based on [2, 8]
Cancer cells	53.5%	cm^2/cm^3 tissue	Calculated, see text
Microvessels	11.5%	cm^2/cm^3 tissue	Calculated, see text
of which intravascular space	10.0%	cm^2/cm^3 tissue	Based on [9–11]
**Surface areas**			
Tumor cells	2939	cm^2/cm^3 tissue	Calculated, see text
Microvessels	378	cm^2/cm^3 tissue	Calculated, see text
**Basement membranes (BM)**			
Thickness of tumor cell BM	30	nm	Based on [32]
Basement membrane volume (tumor cells)	0.00807	cm^3/cm^3	Calculated, see text
of which available to VEGF	0.00508	cm^3/cm^3 tissue	Calculated, see text
Thickness of microvessel BM	50	nm	Based on [32]
Basement membrane volume (microvessels)	0.00154	cm^3/cm^3 tissue	Calculated, see text
of which available to VEGF	0.000972	cm^3/cm^3 tissue	Calculated, see text
Extracellular matrix volume	0.3375	cm^3/cm^3 tissue	Calculated, see text
of which available to VEGF	0.2892	cm^3/cm^3 tissue	Calculated, see text

Table S1.2. Initial concentrations

**Normal compartment**		
	**Value**	**Units**
**VEGFR-1**		
Abluminal EC	0	dimers/EC
Muscle fibers	0	dimers/fiber
**VEGFR-2**		
Abluminal EC	0	dimers/EC
Muscle fibers	0	dimers/ fiber
**NRP-1**		
Abluminal EC	39748	dimers/EC
Muscle fibers	39500	dimers/fiber
**NRP-2**		
Abluminal EC	0	dimers/EC
Muscle fibers	0	dimers/fiber
**ECM binding density**	0.75	μM
**EBM binding density**	13	μM
**PBM binding density**	13	μM

**Blood compartment**		
	**Value**	**Units**
**VEGFR-1**		
Luminal EC (normal)	3750	dimers/EC
Luminal EC (diseased)	3750	dimers/EC
**VEGFR-2**		
Luminal EC (normal)	3750	dimers/EC
Luminal EC (diseased)	3750	dimers/EC
**NRP-1**		
Luminal EC (normal)	3750	dimers/EC
Luminal EC (diseased)	3750	dimers/EC
**NRP-2**		
Luminal EC (normal)	0	dimers/EC
Luminal EC (diseased)	0	dimers/EC
**ECM binding density**	0	μM
**EBM binding density**	0	μM
**PBM binding density**	0	μM

**Tumor compartment**	**Value**	**Units**
**VEGFR-1**		
Abluminal EC	3750	dimers/EC
Tumor cells	1100	dimers/TC
**VEGFR-2**		
Abluminal EC	300	dimers/EC
Tumor cells	550	dimers/ TC
**NRP-1**		
Abluminal EC	39748	dimers/EC
Tumor cells	39500	dimers/TC
**NRP-2**		
Tumor cells	39500	dimers/TC
**ECM binding density**	0.75	μM
**EBM binding density**	13	μM
**PBM binding density**	13	μM

EC = endothelial cell; TC = tumor cell

Table S1.3. Kinetic parameters

	Value	Unit	Reference
**VEGF binding to VEGFR-1**			
k_{on}	3×10^7	$M^{-1}s^{-1}$	[12, 13]
k_{off}	10^{-3}	s^{-1}	[12, 13]
K_d	33	pM	[12, 13]
**VEGF binding to VEGFR-2**			
k_{on}	10^7	$M^{-1}s^{-1}$	[12, 13]
k_{off}	10^{-3}	s^{-1}	[12, 13]
K_d	100	pM	[12, 13]
**VEGF binding to NRP-1**			
k_{on}	3.2×10^6	$M^{-1}s^{-1}$	[12, 13]
k_{off}	10^{-3}	s^{-1}	[12, 13]
K_d	312.5	pM	[12, 13]
**VEGF binding to GAGs**			
k_{on}	4.20×10^5	$M^{-1}s^{-1}$	[12, 13]
k_{off}	10^{-2}	s^{-1}	[12, 13]
K_d	24	pM	[12, 13]
**Coupling of NRP-1 and VEGFR-1**			
k_c	10^{14}	$(mol/cm^2)^{-1}\,s^{-1}$	[12, 13]
k_{off}	10^{-2}	s^{-1}	[12, 13]
**Coupling of NRP-1 and VEGFR-2**			
$k_{cV165R2,N1}$	3.1×10^{13}	$(mol/cm^2)^{-1}\,s^{-1}$	[12, 13]
$k_{offV165R2,N1}$	10^{-3}	s^{-1}	[12, 13]
$k_{cV165N1,R2}$	10^{14}	$(mol/cm^2)^{-1}\,s^{-1}$	[12, 13]
$k_{offV165N1,R2}$	10^{-3}	s^{-1}	[12, 13]
**VEGFR Internalization**			
k_{int}	2.8×10^{-4}	s^{-1}	[12, 13]

**VEGF$_{121}$ binding to anti-VEGF**			
k_{on}	5.4×10^4	$M^{-1}s^{-1}$	[19]
k_{off}	2.19×10^{-5}	s^{-1}	[19]
K_d	4456	pM	[19]
**VEGF$_{165}$ binding to anti-VEGF**			
k_{on}	5.4×10^4	$M^{-1}s^{-1}$	[19]
k_{off}	2.19×10^{-5}	s^{-1}	[19]
K_d	4456	pM	[19]
**VEGF binding to α2M**			
k_{on}	25	$M^{-1}s^{-1}$	Calculated
k_{off}	10^{-4}	s^{-1}	Assumed
K_d	4.0	μM	[33]
**VEGF binding to α2M$_{fast}$**			
k_{on}	2.4×10^2	$M^{-1}s^{-1}$	Calculated
k_{off}	10^{-4}	s^{-1}	Assumed
K_d	0.42	μM	[33]
**sVEGFR1 binding to VEGF**			
k_{on}	3×10^7	$M^{-1}s^{-1}$	Assumed, based on VEGF binding to VEGFR1
k_{off}	10^{-3}	s^{-1}	Assumed
K_d	33	pM	Assumed
**sVEGFR1 binding to NRP-1**			
k_{on}	5.6×10^6	$M^{-1}s^{-1}$	Calculated
k_{off}	10^{-2}	s^{-1}	Assumed, based on VEGFR1 coupling to NRP1
K_d	1.8	nM	[34]
**sVEGFR1 binding to GAGs**			
k_{on}	4.20×10^5	$M^{-1}s^{-1}$	Assumed, based on VEGF$_{165}$ binding to GAG
k_{off}	10^{-2}	s^{-1}	Assumed
K_d	24	pM	Assumed

Table S1.4. Transport, secretion and clearance rates

	Value	Unit	Reference
**Permeability between normal and blood**			
VEGF	4.0×10^{-8}	cm/s	[13]
Anti-VEGF & VEGF/anti-VEGF complex	3.0×10^{-8}	cm/s	[13]
Soluble VEGFR1	1.5×10^{-8}	cm/s	Calculated, see text
Soluble VEGFR1/VEGF complex	1.5×10^{-8}	cm/s	Calculated, see text
**Permeability between tumor and blood**			
VEGF	4.0×10^{-7}	cm/s	Assumed, see text
Anti-VEGF & VEGF/anti-VEGF complex	3.0×10^{-7}	cm/s	Assumed, see text
Soluble VEGFR1	1.5×10^{-7}	cm/s	Assumed, see text
Soluble VEGFR1/VEGF complex	1.5×10^{-7}	cm/s	Assumed, see text
**Clearance**			
VEGF	2.3×10^{-1}	min^{-1}	[35]
Anti-VEGF	8.9×10^{-4}	min^{-1}	[18]
VEGF/anti-VEGF complex	2.8×10^{-4}	min^{-1}	[18]
Soluble VEGFR1	3.0×10^{-4}	min^{-1}	[36]
Soluble VEGFR1/VEGF complex	3.0×10^{-4}	min^{-1}	[36]
Alpha-2-macroglobulin (α2M)	2.6×10^{-3}	min^{-1}	[37]
a2M /VEGF complex	2.6×10^{-3}	min^{-1}	Assumed, based on a2M
a2M /VEGF/anti-VEGF complex	2.6×10^{-3}	min^{-1}	Assumed, based on a2M
Activated alpha-2-macroglobulin ($\alpha 2M_{fast}$)	2.4×10^{-1}	min^{-1}	[38]
a2M /VEGF complex	2.6×10^{-3}	min^{-1}	Assumed, based on $a2M_{fast}$
**Degradation**			
Soluble VEGFR1	1.2×10^{-2}	min^{-1}	Assumed based on VEGF
Soluble VEGFR1/VEGF complex	1.2×10^{-2}	min^{-1}	Assumed based on VEGF
**Synthesis**			
$VEGF_{164}$ – Normal cells	9.72×10^{-3}	molecules/cell/s	Estimated in [15]
$VEGF_{164}$ – Endothelial cells	6.72×10^{-3}	molecules/cell/s	Estimated in [15]
$VEGF_{164}$ – Tumor cells	0	molecules/cell/s	Tumor cells only secrete human VEGF isoforms

$VEGF_{120}$ – Normal cells	8.45×10^{-4}	molecules/cell/s	Estimated in [15]
$VEGF_{120}$ – Endothelial cells	7.47×10^{-4}	molecules/cell/s	Estimated in [15]
$VEGF_{120}$ – Tumor cells	0	molecules/cell/s	Tumor cells only secrete human VEGF isoforms
$VEGF_{165}$ – Normal cells	0	molecules/cell/s	Only tumor cells secrete human VEGF isoforms
$VEGF_{165}$ – Endothelial cells	0	molecules/cell/s	Only tumor cells secrete human VEGF isoforms
$VEGF_{165}$ – Tumor cells	4.65×10^{-3}	molecules/cell/s	Estimated in [15]
$VEGF_{121}$ – Normal cells	0	molecules/cell/s	Only tumor cells secrete human VEGF isoforms
$VEGF_{121}$ – Endothelial cells	0	molecules/cell/s	Only tumor cells secrete human VEGF isoforms
$VEGF_{121}$ – Tumor cells	4.65×10^{-3}	molecules/cell/s	Estimated in [15]
Alpha-2-macroglobulin (α2M)	1.8×10^{10}	molecules/cm^3 tissue/s	Calculated, see text
Activated alpha-2-macroglobulin ($\alpha 2M_{fast}$)	1.6×10^{10}	molecules/cm^3 tissue/s	Calculated, see text

Table S1.5. Equations describing change in relative volume of the interstitial space

Dataset	Relative volume of interstitial space (cm^3/cm^3 tissue)
Roland	$Vol_{IS} = 0.8323 \cdot e^{(-0.239 \cdot V(t))}$
Zibara	$Vol_{IS} = 0.8247 \cdot e^{(-0.069 \cdot V(t))}$
Tan	$Vol_{IS} = 0.8343 \cdot e^{(-0.062 \cdot V(t))}$
Volk (2008)	$Vol_{IS} = 0.8628 \cdot e^{(-0.068 \cdot V(t))}$
Volk (2011a)	$Vol_{IS} = 0.8557 \cdot e^{(-0.081 \cdot V(t))}$
Volk (2011b)	$Vol_{IS} = 0.8536 \cdot e^{(-0.068 \cdot V(t))}$

References

1. Gaddy TD, Wu Q, Arnheim AD, Finley SD. 2017 Mechanistic modeling quantifies the influence of tumor growth kinetics on the response to anti-angiogenic treatment. *PLOS Comput Biol* 13:e1005874 (doi:10.1371/journal.pcbi.1005874).

2. Paran Y, Bendel P, Margalit R, Degani H. 2004 Water diffusion in the different microenvironments of breast cancer. *NMR Biomed* 17:170–80 (doi:10.1002/nbm.882).

3. Schaefer C, Schroeder M, Fuhrhop I, Viezens L, Otten J, Fiedler W, et al. 2011 Primary tumor dependent inhibition of tumor growth, angiogenesis, and perfusion of secondary breast cancer in bone. *J Orthop Res* 29:1251–8 (doi:10.1002/jor.21402).

4. Kim E, Stamatelos S, Cebulla J, Bhujwalla ZM, Popel AS, Pathak AP. 2012 Multiscale imaging and computational modeling of blood flow in the tumor vasculature. *Ann Biomed Eng* 40 (doi:10.1007/s10439-012-0585-5).

5. Stamatelos SK, Kim E, Pathak AP, Popel AS. 2014 A bioimage informatics based reconstruction of breast tumor microvasculature with computational blood flow predictions. *Microvasc Res* 91:8–21 (doi:10.1016/j.mvr.2013.12.003).

6. Olewniczak S, Chosia M, Kołodziej B, Kwas A, Kram A, Domagała W. 2003 Angiogenesis as determined by computerised image analysis and the risk of early relapse in women with invasive ductal breast carcinoma. *Pol J Pathol Off J Pol Soc Pathol* 54:53–9.

7. Olewniczak S, Chosia M, Kwas A, Kram A, Domagała W. 2002 Angiogenesis and some prognostic parameters of invasive ductal breast carcinoma in women. *Pol J Pathol Off J Pol Soc Pathol* 53:183–8.

8. Hassid Y, Furman-Haran E, Margalit R, Eilam R, Degani H. 2006 Noninvasive magnetic resonance imaging of transport and interstitial fluid pressure in ectopic human lung tumors. *Cancer Res* 66:4159–66 (doi:10.1158/0008-5472.CAN-05-3289).

9. Cao M, Liang Y, Shen C, Miller KD, Stantz KM. 2009 Developing DCE-CT to quantify intra-tumor heterogeneity in breast tumors with differing angiogenic phenotype. *IEEE Trans Med Imaging* 28:861–71.

10. Bogin Liora, Margalit Raanan, Mispelter Joel, Degani Hadassa. 2002 Parametric imaging of tumor perfusion using flow- and permeability-limited tracers. *J Magn Reson Imaging* 16:289–99 (doi:10.1002/jmri.10159).

11. Lewin Maïté, Bredow Sebastian, Sergeyev Nikolai, Marecos Edgardo, Bogdanov Alexei, Weissleder Ralph. 1999 In vivo assessment of vascular endothelial growth factor-induced angiogenesis. *Int J Cancer* 83:798–802 (doi:10.1002/(SICI)1097-0215(19991210)83:6<798::AID-IJC16>3.0.CO;2-W).

12. Gabhann FM, Popel AS. 2006 Targeting neuropilin-1 to inhibit vegf signaling in cancer: comparison of therapeutic approaches. *PLOS Comput Biol* 2:e180 (doi:10.1371/journal.pcbi.0020180).

13. Stefanini MO, Wu FT, Mac Gabhann F, Popel AS. 2008 A compartment model of VEGF distribution in blood, healthy and diseased tissues. *BMC Syst Biol* 2:77 (doi:10.1186/1752-0509-2-77).

14. Finley SD, Engel-Stefanini MO, Imoukhuede P, Popel AS. 2011 Pharmacokinetics and pharmacodynamics of VEGF-neutralizing antibodies. *BMC Syst Biol* 5:193 (doi:10.1186/1752-0509-5-193).

15. Finley SD, Dhar M, Popel AS. 2013 Compartment model predicts VEGF secretion and investigates the effects of VEGF trap in tumor-bearing mice. *Front Oncol* 3 (doi:10.3389/fonc.2013.00196).

16. Yuan F, Krol A, Tong S. 2001 Available space and extracellular transport of macromolecules: effects of pore size and connectedness. *Ann Biomed Eng* 29:1150–8.

17. Hashizume H, Baluk P, Morikawa S, McLean JW, Thurston G, Roberge S, et al. 2000 Openings between defective endothelial cells explain tumor vessel leakiness. *Am J Pathol* 156:1363–80 https://www.ncbi.nlm.nih.gov/pmc/articles/PMC1876882/. Accessed 6 Apr 2018.

18. Yen P, Finley SD, Engel-Stefanini MO, Popel AS. 2011 A two-compartment model of VEGF distribution in the mouse. *PLOS ONE* 6:e27514 (doi:10.1371/journal.pone.0027514).

19. Yang J, Wang X, Fuh G, Yu L, Wakshull E, Khosraviani M, et al. 2014 Comparison of binding characteristics and in vitro activities of three inhibitors of vascular endothelial growth factor a. *Mol Pharm* 10:3421–30 (doi:10.1021/mp500160v).

20. Stimpfl M, Tong D, Fasching B, Schuster E, Obermair A, Leodolter S, et al. 2002 Vascular endothelial growth factor splice variants and their prognostic value in breast and ovarian cancer. *Clin Cancer Res* 8:2253–9 http://clincancerres.aacrjournals.org/content/8/7/2253. Accessed 6 Apr 2018.

21. Yuan A, Yu CJ, Luh KT, Chen WJ, Lin FY, Kuo SH, et al. 2000 Quantification of VEGF mRNA expression in non-small cell lung cancer using a real-time quantitative reverse transcription-PCR assay and a comparison with quantitative competitive reverse transcription-PCR. *Lab Investig J Tech Methods Pathol* 80:1671–80.

22. Cheung N, Wong MP, Yuen ST, Leung SY, Chung LP. 1998 Tissue-specific expression pattern of vascular endothelial growth factor isoforms in the malignant transformation of lung and colon. *Hum Pathol* 29:910–4.

23. Ljungberg B, Jacobsen J, Häggström-Rudolfssson S, Rasmuson T, Lindh G, Grankvist K. 2003 Tumour vascular endothelial growth factor (VEGF) mRNA in relation to serum VEGF protein levels and tumour progression in human renal cell carcinoma. *Urol Res* 31:335–40.

24. Zygalaki E, Tsaroucha EG, Kaklamanis L, Lianidou ES. 2007 Quantitative real-time reverse transcription–pcr study of the expression of vascular endothelial growth factor (VEGF) splice variants and vegf receptors (VEGFR-1 and VEGFR-2) in non–small cell lung cancer. *Clin Chem* 53:1433–9 (doi:10.1373/clinchem.2007.086819).

25. Rudge JS, Holash J, Hylton D, Russell M, Jiang S, Leidich R, et al. 2007 VEGF Trap complex formation measures production rates of VEGF, providing a biomarker for predicting

efficacious angiogenic blockade. *Proc Natl Acad Sci U S A* 104:18363–70 (doi:10.1073/pnas.0708865104).

26. Garlick D, Renkin E. 1970 Transport of large molecules from plasma to interstitial fluid and lymph in dogs. *Am J Physiol-Leg Content* 219:1595–605 (doi:10.1152/ajplegacy.1970.219.6.1595).

27. Goel S, Duda DG, Xu L, Munn LL, Boucher Y, Fukumura D, et al. 2011 Normalization of the vasculature for treatment of cancer and other diseases. *Physiol Rev* 91:1071–121 (doi:10.1152/physrev.00038.2010).

28. Christensen J, Vonwil D, Shastri VP. 2015 Non-Invasive In Vivo Imaging and Quantification of Tumor Growth and Metastasis in Rats Using Cells Expressing Far-Red Fluorescence Protein. *PLOS ONE* 10:e0132725 (doi:10.1371/journal.pone.0132725).

29. Simeoni M, Magni P, Cammia C, Nicolao GD, Croci V, Pesenti E, et al. 2004 Predictive Pharmacokinetic-Pharmacodynamic Modeling of Tumor Growth Kinetics in Xenograft Models after Administration of Anticancer Agents. *Cancer Res* 64:1094–101 (doi:10.1158/0008-5472.CAN-03-2524).

30. Sharan S, Woo S. 2014 Quantitative Insight in Utilizing Circulating Angiogenic Factors as Biomarkers for Antiangiogenic Therapy: Systems Pharmacology Approach. *CPT Pharmacomet Syst Pharmacol* 3:e139 (doi:10.1038/psp.2014.36).

31. Hall J. 2011 The circulation. In: Guyton and Hall Textbook of Medical Physiology - 12th Edition. W.B. Saunders Co; 2011 https://www.elsevier.com/books/guyton-and-hall-textbook-of-medical-physiology/hall/978-0-8089-2400-5. Accessed 6 Apr 2018.

32. Baluk P, Morikawa S, Haskell A, Mancuso M, McDonald DM. 2003 Abnormalities of basement membrane on blood vessels and endothelial sprouts in tumors. *Am J Pathol* 163:1801–15 https://www.ncbi.nlm.nih.gov/pmc/articles/PMC1892429/. Accessed 6 Apr 2018.

33. Bhattacharjee G, Asplin IR, Wu SM, Gawdi G, Pizzo SV. 2000 The conformation-dependent interaction of alpha2-macroglobulin with vascular endothelial growth factor: a novel mechanism of alpha2-macroglobulin/growth factor binding. *J Biol Chem* (doi:10.1074/jbc.M000156200).

34. Fuh G, Garcia KC, Vos AM de. 2000 The interaction of neuropilin-1 with vascular endothelial growth factor and its receptor flt-1. *J Biol Chem* 275:26690–5 http://www.jbc.org/content/275/35/26690. Accessed 6 Apr 2018.

35. Folkman J. 1995 Angiogenesis in cancer, vascular, rheumatoid and other disease. *Nat Med* 1:27–31.

36. Wu FTH, Stefanini MO, Gabhann FM, Popel AS. 2009 A compartment model of VEGF distribution in humans in the presence of soluble VEGF receptor-1 acting as a ligand trap. *PLOS ONE* 4:e5108 (doi:10.1371/journal.pone.0005108).

37. Hudson NW, Kehoe JM, Koo PH. 1987 Mouse alpha-macroglobulin. Structure, function and a molecular model. *Biochem J* 248:837–45 https://www.ncbi.nlm.nih.gov/pmc/articles/PMC1148625/. Accessed 6 Apr 2018.

38. Imber MJ, Pizzo SV. 1981 Clearance and binding of two electrophoretic “fast” forms of human alpha 2-macroglobulin. *J Biol Chem* 256:8134–9 http://www.jbc.org/content/256/15/8134. Accessed 6 Apr 2018.
