# Supplementary material for: In silico mouse study identifies tumour growth kinetics as biomarkers for the outcome of anti-angiogenic treatment
Source: J R Soc Interface. 2018 Aug 22;15(145):20180243. doi: 10.1098/rsif.2018.0243 (PMC6127173; doi:10.1098/rsif.2018.0243)
Supplement: File S2. Compressed file containing the computational model [file rsif20180243supp2.gz › Supplemental_File_S2/List of Files.pdf]

Article Title: ***In silico* mouse study identifies tumor growth kinetics as biomarkers for the outcome of anti-angiogenic treatment**

Authors: Qianhui Wu Alyssa D. Arnheim, and Stacey D. Finley

Supplemental File S2. Zipped file containing the computational model in various formats:

1. MATLAB SimBiology file ("Model_SimBiology.sbproj")
2. SBML file ("Model_SBML.xml")
3. MATLAB .m file ("Model_equations.m"), with script needed to run the model ("Model_driver.m")
4. File containing the full list of model reactions and equations ("Model_reactions_and_equations.pdf")
