# Supplementary material for: In silico mouse study identifies tumour growth kinetics as biomarkers for the outcome of anti-angiogenic treatment
Source: J R Soc Interface. 2018 Aug 22;15(145):20180243. doi: 10.1098/rsif.2018.0243 (PMC6127173; doi:10.1098/rsif.2018.0243)
Supplement: File S2. Compressed file containing the computational model [file rsif20180243supp2.gz › Supplemental_File_S2/Model_reactions_and_equations.pdf]

I. Chemical reactions

The relevant chemical reactions are presented here (molecular species and parameters are defined in the glossary):

Mouse isoforms

VEGF$_{164}$ binding GAG chains in ECM and basement membranes (EBM and PBM)

$$V_{164} + M_{ECM} \underset{k_{off,V164MECM}}{\overset{k_{on,V164,MECM}}{\rightleftarrows}} V_{164}M_{ECM}$$

$$V_{164} + M_{EBM} \underset{k_{off,V164MEBM}}{\overset{k_{on,V164,MEBM}}{\rightleftarrows}} V_{164}M_{EBM}$$

$$V_{164} + M_{PBM} \underset{k_{off,V164MPBM}}{\overset{k_{on,V164,MPBM}}{\rightleftarrows}} V_{164}M_{PBM}$$

VEGF$_{164}$ binding receptors

$$V_{164} + R_1 \underset{k_{off,V164,R1}}{\overset{k_{on,V164,R1}}{\rightleftarrows}} V_{164}R_1$$

$$V_{164} + R_2 \underset{k_{off,V164R2}}{\overset{k_{on,V164,R2}}{\rightleftarrows}} V_{164}R_2$$

$$V_{164} + N_1 \underset{k_{off,V164N1}}{\overset{k_{on,V164,N1}}{\rightleftarrows}} V_{164}N_1$$

$$V_{164}N_1 + R_2 \underset{k_{off,V164N1R2}}{\overset{k_{c,V164N1,R2}}{\rightleftarrows}} R_2V_{164}N_1$$

$$V_{164}R_2 + N_1 \underset{k_{off,V164R2N1}}{\overset{k_{c,V164R2,N1}}{\rightleftarrows}} R_2V_{164}N_1$$

$$V_{164} + N_2 \underset{k_{off,V164,N2}}{\overset{k_{on,V164,N2}}{\rightleftarrows}} V_{164}N_2$$

$$V_{164}N_2 + R_2 \underset{k_{off,V164N2R2}}{\overset{k_{c,V164N2,R2}}{\rightleftarrows}} R_2V_{164}N_2$$

$$V_{164}N_2 + R_2 \underset{k_{off,V164R2N2}}{\overset{k_{c,V164R2,N2}}{\rightleftarrows}} R_2V_{164}N_2$$

$$V_{164} + sR_1 \underset{k_{off,V164sR1}}{\overset{k_{on,V164,sR1}}{\rightleftarrows}} V_{164}sR_1$$

VEGF$_{120}$ binding receptors

$$V_{120} + R_1 \underset{k_{off,V120R1}}{\overset{k_{on,V120,R1}}{\rightleftarrows}} V_{120}R_1$$

$$V_{120} + R_2 \underset{k_{off,V120R2}}{\overset{k_{on,V120,R2}}{\rightleftarrows}} V_{120}R_2$$

$$V_{120} + R_1N_1 \underset{k_{off,V120R1}}{\overset{k_{on,V120,R1}}{\rightleftarrows}} V_{120}R_1N_1$$

$$V_{120}R_1 + N_1 \underset{k_{dissoc,R1N1}}{\overset{k_{c,R1,N1}}{\rightleftarrows}} V_{120}R_1N_1$$

$$V_{120} + R_1N_2 \underset{k_{off,V120R1}}{\overset{k_{on,V120,R1}}{\rightleftarrows}} V_{120}R_1N_2$$

$$V_{120}R_1 + N_2 \underset{k_{dissoc,R1N2}}{\overset{k_{c,R1,N2}}{\rightleftarrows}} V_{120}R_1N_2$$

$$V_{120} + sR_1 \underset{k_{off,V120sR1}}{\overset{k_{on,V120,sR1}}{\rightleftarrows}} V_{120}sR_1$$

$$V_{120} + sR_1N_1 \underset{k_{off,V120sR1}}{\overset{k_{on,V120,sR1}}{\rightleftarrows}} V_{120}sR_1N_1$$

$$V_{120}sR_1 + N_1 \underset{k_{off,sR1N1}}{\overset{k_{on,sR1,N1}}{\rightleftarrows}} V_{120}sR_1N_1$$

$$V_{120} + sR_1N_2 \underset{k_{off,V120sR1}}{\overset{k_{on,V120,sR1}}{\rightleftarrows}} V_{120}sR_1N_2$$

$$V_{120}sR_1 + N_2 \underset{k_{off,sR1N2}}{\overset{k_{on,sR1,N2}}{\rightleftarrows}} V_{120}sR_1N_2$$

VEGF binding anti-VEGF (A)

$$V_{164} + A \underset{k_{off,V164A}}{\overset{k_{on,V164,A}}{\rightleftarrows}} V_{164}A$$

$$V_{120} + A \underset{k_{off,V120A}}{\overset{k_{on,V120,A}}{\rightleftarrows}} V_{120}A$$

VEGF binding alpha-2-macroglobulin

$$V_{164} + \alpha 2\text{M} \underset{k_{off,V164,\alpha 2\text{M}}}{\overset{k_{on,V164,\alpha 2\text{M}}}{\rightleftarrows}} \alpha 2\text{M} \cdot V_{164}$$

$$V_{120} + \alpha 2\text{M} \underset{k_{off,V120,\alpha 2\text{M}}}{\overset{k_{on,V120,\alpha 2\text{M}}}{\rightleftarrows}} \alpha 2\text{M} \cdot V_{120}$$

$$\alpha 2\text{M} \cdot V_{164} + A \underset{k_{off,V164A}}{\overset{k_{on,V164,A}}{\rightleftarrows}} \alpha 2\text{M} \cdot V_{164}A$$

$$\alpha 2\text{M} \cdot V_{120} + A \underset{k_{off,V120A}}{\overset{k_{on,V120,A}}{\rightleftarrows}} \alpha 2\text{M} \cdot V_{120}A$$

$$\alpha 2\text{M} \cdot V_{164} + R_1 \underset{k_{off,V164R1}}{\overset{k_{on,V164,R1}}{\rightleftarrows}} \alpha 2\text{M} \cdot V_{164}R_1$$

$$\alpha 2\text{M} \cdot V_{164} + R_2 \underset{k_{off,V164R2}}{\overset{k_{on,V164,R2}}{\rightleftarrows}} \alpha 2\text{M} \cdot V_{164}R_2$$

$$\alpha 2\text{M} \cdot V_{164} + N_1 \underset{k_{off,V164N1}}{\overset{k_{on,V164,N1}}{\rightleftarrows}} \alpha 2\text{M} \cdot V_{164}N_1$$

$$\alpha 2\mathrm{M}\cdot V_{164}N_1 + R_2 \underset{k_{off,V164N1R2}}{\overset{k_{c,V164N1,R2}}{\rightleftarrows}} \alpha 2\mathrm{M}\cdot R_2V_{164}N_1$$

$$\alpha 2\mathrm{M}\cdot V_{164}R_2 + N_1 \underset{k_{off,V164R2N1}}{\overset{k_{c,V164R2,N1}}{\rightleftarrows}} \alpha 2\mathrm{M}\cdot R_2V_{164}N_1$$

$$\alpha 2\mathrm{M}\cdot V_{120} + R_1 \underset{k_{off,V120R1}}{\overset{k_{on,V120,R1}}{\rightleftarrows}} \alpha 2\mathrm{M}\cdot V_{120}R_1$$

$$\alpha 2\mathrm{M}\cdot V_{120} + R_2 \underset{k_{off,V120R2}}{\overset{k_{on,V120,R2}}{\rightleftarrows}} \alpha 2\mathrm{M}\cdot V_{120}R_2$$

$$\alpha 2\mathrm{M}\cdot V_{120} + R_1N_1 \underset{k_{off,V120R1}}{\overset{k_{on,V120,R1}}{\rightleftarrows}} \alpha 2\mathrm{M}\cdot V_{120}R_1N_1$$

$$\alpha 2\mathrm{M}\cdot V_{120}R_1 + N_1 \underset{k_{dissoc,R1N1}}{\overset{k_{c,R1,N1}}{\rightleftarrows}} \alpha 2\mathrm{M}\cdot V_{120}R_1N_1$$

$$V_{164} + \alpha 2\mathrm{M}_{fast} \underset{k_{off,V164,\alpha 2\mathrm{M}}}{\overset{k_{on,V164,\alpha 2\mathrm{M}}}{\rightleftarrows}} \alpha 2\mathrm{M}_{fast}\cdot V_{164}$$

$$V_{120} + \alpha 2\mathrm{M}_{fast} \underset{k_{off,V120,\alpha 2\mathrm{M}}}{\overset{k_{on,V120,\alpha 2\mathrm{M}}}{\rightleftarrows}} \alpha 2\mathrm{M}_{fast}\cdot V_{120}$$

$$\alpha 2\mathrm{M}_{fast}\cdot V_{164} + R_1 \underset{k_{off,V164R1}}{\overset{k_{on,V164,R1}}{\rightleftarrows}} \alpha 2\mathrm{M}_{fast}\cdot V_{164}R_1$$

$$\alpha 2\mathrm{M}_{fast}\cdot V_{164} + R_2 \underset{k_{off,V164R2}}{\overset{k_{on,V164,R2}}{\rightleftarrows}} \alpha 2\mathrm{M}_{fast}\cdot V_{164}R_2$$

$$\alpha 2\mathrm{M}_{fast}\cdot V_{164} + N_1 \underset{k_{off,V164N1}}{\overset{k_{on,V164,N1}}{\rightleftarrows}} \alpha 2\mathrm{M}_{fast}\cdot V_{164}N_1$$

$$\alpha 2\mathrm{M}_{fast}\cdot V_{164}N_1 + R_2 \underset{k_{off,V164N1R2}}{\overset{k_{c,V164N1,R2}}{\rightleftarrows}} \alpha 2\mathrm{M}_{fast}\cdot R_2V_{164}N_1$$

$$\alpha 2\mathrm{M}_{fast}\cdot V_{164}R_2 + N_1 \underset{k_{off,V164R2N1}}{\overset{k_{c,V164R2,N1}}{\rightleftarrows}} \alpha 2\mathrm{M}_{fast}\cdot R_2V_{164}N_1$$

$$\alpha 2\mathrm{M}_{fast}\cdot V_{120} + R_1 \underset{k_{off,V120R1}}{\overset{k_{on,V120,R1}}{\rightleftarrows}} \alpha 2\mathrm{M}_{fast}\cdot V_{120}R_1$$

$$\alpha 2\mathrm{M}_{fast}\cdot V_{120} + R_2 \underset{k_{off,V120R2}}{\overset{k_{on,V120,R2}}{\rightleftarrows}} \alpha 2\mathrm{M}_{fast}\cdot V_{120}R_2$$

$$\alpha 2\mathrm{M}_{fast}\cdot V_{120} + R_1N_1 \underset{k_{off,V120R1}}{\overset{k_{on,V120,R1}}{\rightleftarrows}} \alpha 2\mathrm{M}_{fast}\cdot V_{120}R_1N_1$$

$$\alpha 2\mathrm{M}_{fast}\cdot V_{120}R_1 + N_1 \underset{k_{dissoc,R1N1}}{\overset{k_{c,R1,N1}}{\rightleftarrows}} \alpha 2\mathrm{M}_{fast}\cdot V_{120}R_1N_1$$

Human isoforms

Binding GAG chains in ECM and basement membranes (EBM and PBM)

$$V_{165} + M_{ECM} \underset{k_{off,V165MECM}}{\overset{k_{on,V165,MECM}}{\rightleftarrows}} V_{165}M_{ECM}$$

$$V_{165}+M_{EBM} \underset{k_{off,V165MEBM}}{\overset{k_{on,V165,MEBM}}{\rightleftarrows}} V_{165}M_{EBM}$$

$$V_{165}+M_{PBM} \underset{k_{off,V165MPBM}}{\overset{k_{on,V165,MPBM}}{\rightleftarrows}} V_{165}M_{PBM}$$

VEGF$_{165}$ binding receptors

$$V_{165}+R_1 \underset{k_{off,V165,R1}}{\overset{k_{on,V165,R1}}{\rightleftarrows}} V_{165}R_1$$

$$V_{165}+R_2 \underset{k_{off,V165R2}}{\overset{k_{on,V165,R2}}{\rightleftarrows}} V_{165}R_2$$

$$V_{165}+N_1 \underset{k_{off,V165N1}}{\overset{k_{on,V165,N1}}{\rightleftarrows}} V_{165}N_1$$

$$V_{165}N_1+R_2 \underset{k_{off,V165N1R2}}{\overset{k_{c,V165N1,R2}}{\rightleftarrows}} R_2V_{165}N_1$$

$$V_{165}R_2+N_1 \underset{k_{off,V165R2N1}}{\overset{k_{c,V165R2,N1}}{\rightleftarrows}} R_2V_{165}N_1$$

$$V_{165}+N_2 \underset{k_{on,V165,N2}}{\overset{k_{on,V165,N2}}{\rightleftarrows}} V_{165}N_2$$

$$V_{165}N_2+R_2 \underset{k_{off,V165N2R2}}{\overset{k_{c,V165N2,R2}}{\rightleftarrows}} R_2V_{165}N_2$$

$$V_{165}N_2+R_2 \underset{k_{off,V165R2N2}}{\overset{k_{c,V165R2,N2}}{\rightleftarrows}} R_2V_{165}N_2$$

$$V_{165}+sR_1 \underset{k_{off,V165sR1}}{\overset{k_{on,V165,sR1}}{\rightleftarrows}} V_{165}sR_1$$

VEGF$_{121}$ binding receptors

$$V_{121}+R_1 \underset{k_{off,V121R1}}{\overset{k_{on,V121,R1}}{\rightleftarrows}} V_{121}R_1$$

$$V_{121}+R_2 \underset{k_{off,V121R2}}{\overset{k_{on,V121,R2}}{\rightleftarrows}} V_{121}R_2$$

$$V_{121}+R_1N_1 \underset{k_{off,V121R1}}{\overset{k_{on,V121,R1}}{\rightleftarrows}} V_{121}R_1N_1$$

$$V_{121}R_1+N_1 \underset{k_{dissoc,R1N1}}{\overset{k_{c,R1,N1}}{\rightleftarrows}} V_{121}R_1N_1$$

$$V_{121}+R_1N_2 \underset{k_{off,V121R1}}{\overset{k_{on,V121,R1}}{\rightleftarrows}} V_{121}R_1N_2$$

$$V_{121}R_1+N_2 \underset{k_{dissoc,R1N2}}{\overset{k_{c,R1,N2}}{\rightleftarrows}} V_{121}R_1N_2$$

$$V_{121}+sR_1 \underset{k_{off,V121sR1}}{\overset{k_{on,V121,sR1}}{\rightleftarrows}} V_{121}sR_1$$

$$V_{121} + sR_1N_1 \xrightleftharpoons[k_{off,V121sR1}]{k_{on,V121,sR1}} V_{121}sR_1N_1$$

$$V_{121}sR_1 + N_1 \xrightleftharpoons[k_{off,sR1N1}]{k_{on,sR1,N1}} V_{121}sR_1N_1$$

$$V_{121} + sR_1N_2 \xrightleftharpoons[k_{off,V121sR1}]{k_{on,V121,sR1}} V_{121}sR_1N_2$$

$$V_{121}sR_1 + N_2 \xrightleftharpoons[k_{off,sR1N2}]{k_{on,sR1,N2}} V_{121}sR_1N_2$$

VEGF binding anti-VEGF (A)

$$V_{165} + A \xrightleftharpoons[k_{off,V165A}]{k_{on,V165,A}} V_{165}A$$

$$V_{121} + A \xrightleftharpoons[k_{off,V121A}]{k_{on,V121,A}} V_{121}A$$

VEGF binding alpha-2-macroglobulin

$$V_{165} + \alpha 2\mathrm{M} \xrightleftharpoons[k_{off,V165\alpha 2\mathrm{M}}]{k_{on,V165,\alpha 2\mathrm{M}}} \alpha 2\mathrm{M} \cdot V_{165}$$

$$V_{121} + \alpha 2\mathrm{M} \xrightleftharpoons[k_{off,V165\alpha 2\mathrm{M}}]{k_{on,V165,\alpha 2\mathrm{M}}} \alpha 2\mathrm{M} \cdot V_{121}$$

$$\alpha 2\mathrm{M} \cdot V_{165} + A \xrightleftharpoons[k_{off,V165A}]{k_{on,V165,A}} \alpha 2\mathrm{M} \cdot V_{165}A$$

$$\alpha 2\mathrm{M} \cdot V_{121} + A \xrightleftharpoons[k_{off,V121A}]{k_{on,V121,A}} \alpha 2\mathrm{M} \cdot V_{121}A$$

$$\alpha 2\mathrm{M} \cdot V_{165} + R_1 \xrightleftharpoons[k_{off,V165,R1}]{k_{on,V165,R1}} \alpha 2\mathrm{M} \cdot V_{165}R_1$$

$$\alpha 2\mathrm{M} \cdot V_{165} + R_2 \xrightleftharpoons[k_{off,V165R2}]{k_{on,V165,R2}} \alpha 2\mathrm{M} \cdot V_{165}R_2$$

$$\alpha 2\mathrm{M} \cdot V_{165} + N_1 \xrightleftharpoons[k_{off,V165N1}]{k_{on,V165,N1}} \alpha 2\mathrm{M} \cdot V_{165}N_1$$

$$\alpha 2\mathrm{M} \cdot V_{165}N_1 + R_2 \xrightleftharpoons[k_{off,V165N1R2}]{k_{c,V165N1,R2}} \alpha 2\mathrm{M} \cdot R_2V_{165}N_1$$

$$\alpha 2\mathrm{M} \cdot V_{165}R_2 + N_1 \xrightleftharpoons[k_{off,V165R2N1}]{k_{c,V165R2,N1}} \alpha 2\mathrm{M} \cdot R_2V_{165}N_1$$

$$\alpha 2\mathrm{M} \cdot V_{121} + R_1 \xrightleftharpoons[k_{off,V121R1}]{k_{on,V121,R1}} \alpha 2\mathrm{M} \cdot V_{121}R_1$$

$$\alpha 2\mathrm{M} \cdot V_{121} + R_2 \xrightleftharpoons[k_{off,V121R2}]{k_{on,V121,R2}} \alpha 2\mathrm{M} \cdot V_{121}R_2$$

$$\alpha 2\mathrm{M} \cdot V_{121} + R_1N_1 \xrightleftharpoons[k_{off,V121R1}]{k_{on,V121,R1}} \alpha 2\mathrm{M} \cdot V_{121}R_1N_1$$

$$\alpha 2\mathrm{M}\cdot V_{121}R_1 + N_1 \underset{k_{dissoc,R1N1}}{\overset{k_{c,R1,N1}}{\rightleftarrows}} \alpha 2\mathrm{M}\cdot V_{121}R_1N_1$$

$$V_{165} + \alpha 2\mathrm{M}_{fast} \underset{k_{off,V165\alpha 2\mathrm{M}fast}}{\overset{k_{on,V165,\alpha 2\mathrm{M}fast}}{\rightleftarrows}} \alpha 2\mathrm{M}_{fast}\cdot V_{165}$$

$$V_{121} + \alpha 2\mathrm{M}_{fast} \underset{k_{off,V165\alpha 2\mathrm{M}fast}}{\overset{k_{on,V165,\alpha 2\mathrm{M}fast}}{\rightleftarrows}} \alpha 2\mathrm{M}_{fast}\cdot V_{121}$$

$$\alpha 2\mathrm{M}_{fast}\cdot V_{165} + R_1 \underset{k_{off,V165,R1}}{\overset{k_{on,V165,R1}}{\rightleftarrows}} \alpha 2\mathrm{M}_{fast}\cdot V_{165}R_1$$

$$\alpha 2\mathrm{M}_{fast}\cdot V_{165} + R_2 \underset{k_{off,V165R2}}{\overset{k_{on,V165,R2}}{\rightleftarrows}} \alpha 2\mathrm{M}_{fast}\cdot V_{165}R_2$$

$$\alpha 2\mathrm{M}_{fast}\cdot V_{165} + N_1 \underset{k_{off,V165N1}}{\overset{k_{on,V165,N1}}{\rightleftarrows}} \alpha 2\mathrm{M}_{fast}\cdot V_{165}N_1$$

$$\alpha 2\mathrm{M}_{fast}\cdot V_{165}N_1 + R_2 \underset{k_{off,V165N1R2}}{\overset{k_{c,V165N1,R2}}{\rightleftarrows}} \alpha 2\mathrm{M}_{fast}\cdot R_2V_{165}N_1$$

$$\alpha 2\mathrm{M}_{fast}\cdot V_{165}R_2 + N_1 \underset{k_{off,V165R2N1}}{\overset{k_{c,V165R2,N1}}{\rightleftarrows}} \alpha 2\mathrm{M}_{fast}\cdot R_2V_{165}N_1$$

$$\alpha 2\mathrm{M}_{fast}\cdot V_{121} + R_1 \underset{k_{off,V121R1}}{\overset{k_{on,V121,R1}}{\rightleftarrows}} \alpha 2\mathrm{M}_{fast}\cdot V_{121}R_1$$

$$\alpha 2\mathrm{M}_{fast}\cdot V_{121} + R_2 \underset{k_{off,V121R2}}{\overset{k_{on,V121,R2}}{\rightleftarrows}} \alpha 2\mathrm{M}_{fast}\cdot V_{121}R_2$$

$$\alpha 2\mathrm{M}_{fast}\cdot V_{121} + R_1N_1 \underset{k_{off,V121R1}}{\overset{k_{on,V121,R1}}{\rightleftarrows}} \alpha 2\mathrm{M}_{fast}\cdot V_{121}R_1N_1$$

$$\alpha 2\mathrm{M}_{fast}\cdot V_{121}R_1 + N_1 \underset{k_{dissoc,R1N1}}{\overset{k_{c,R1,N1}}{\rightleftarrows}} \alpha 2\mathrm{M}_{fast}\cdot V_{121}R_1N_1$$

Receptor coupling

$$R_1 + N_1 \underset{k_{dissoc,R1N1}}{\overset{k_{c,R1,N1}}{\rightleftarrows}} R_1N_1$$

$$R_1 + N_2 \underset{k_{dissoc,R1N2}}{\overset{k_{c,R1,N2}}{\rightleftarrows}} R_1N_2$$

Soluble receptor

$$sR_1 + M_{ECM} \underset{k_{off,sR1MECM}}{\overset{k_{on,sR1,MECM}}{\rightleftarrows}} sR_1M_{ECM}$$

$$sR_1 + M_{EBM} \underset{k_{off,sR1MEBM}}{\overset{k_{on,sR1,MEBM}}{\rightleftarrows}} sR_1M_{EBM}$$

$$sR_1 + M_{PBM} \underset{k_{off,sR1MPBM}}{\overset{k_{on,sR1,MPBM}}{\rightleftarrows}} sR_1M_{PBM}$$

$$sR_1 + N_1 \underset{k_{off,sR1,N1}}{\overset{k_{on,sR1,N1}}{\rightleftarrows}} sR_1N_1$$

$$sR_1 + N_2 \underset{k_{off,sR1,N2}}{\overset{k_{on,sR1,N2}}{\rightleftarrows}} sR_1N_2$$

II. Equations for molecular species

The complete list of ordinary differential equations is presented below:

A. Interstitial space in normal tissue compartment

$$\begin{aligned}\frac{d[V_{164}]_N}{dt} &= q^N_{V164} - k_{\deg,V}[V_{164}]_N - k^N_{on,V164,MEBM}[V_{164}]_N[M_{EBM}]_N + k^N_{off,V164,MEBM}[V_{164}M_{EBM}]_N \\ &\quad - k^N_{on,V164,MPBM}[V_{164}]_N[M_{PBM}]_N + k^N_{off,V164,MPBM}[V_{164}M_{PBM}]_N \\ &\quad - k^N_{on,V164,MECM}[V_{164}]_N[M_{ECM}]_N + k^N_{off,V164,MECM}[V_{164}M_{ECM}]_N \\ &\quad - k^N_{on,V164,R1}[V_{164}]_N[R_1]_N + k^N_{off,V164R1}[V_{164}R_1]_N \\ &\quad - k^N_{on,V164,R2}[V_{164}]_N[R_2]_N + k^N_{off,V164R2}[V_{164}R_2]_N \\ &\quad - k^N_{on,V164,N1}[V_{164}]_N[N_1]_N + k^N_{off,V164N1}[V_{164}N_1]_N \\ &\quad - k^{N,myo}_{on,V164,N1}[V_{164}]_N[N_1]_{N,myo} + k^{N,myo}_{off,V164N1}[V_{164}N_1]_{N,myo} \\ &\quad - k^N_{on,V164,A}[V_{164}]_N[A]_N + k^N_{off,V164A}[V_{164}A]_N \\ &\quad - \left(\frac{k_L + k^{NB}_{p,V}S_{NB}}{U_N}\right)\frac{[V_{164}]_N}{K_{AV,N}} + k^{BN}_{p,V}\frac{S_{NB}}{U_N}\frac{U_B}{U_P}[V_{164}]_B\end{aligned} \qquad \text{(S.1)}$$

$$\begin{aligned}\frac{d[V_{120}]_N}{dt} &= q^N_{V120} - k_{\deg,V}[V_{120}]_N - k^N_{on,V120,R1}[V_{120}]_N[R_1]_N + k^N_{off,V120R1}[V_{120}R_1]_N \\ &\quad - k^N_{on,V120,R1N1}[V_{120}]_N[R_1N_1]_N + k^N_{off,V120R1N1}[V_{120}R_1N_1]_N \\ &\quad - k^N_{on,V120,R2}[V_{120}]_N[R_2]_N + k^N_{off,V120R2}[V_{120}R_2]_N \\ &\quad - k^N_{on,V120,A}[V_{120}]_N[A]_N + k^N_{off,V120A}[V_{120}A]_N \\ &\quad - \left(\frac{k_L + k^{NB}_{p,V}S_{NB}}{U_N}\right)\frac{[V_{120}]_N}{K_{AV,N}} + k^{BN}_{p,V}\frac{S_{NB}}{U_N}\frac{U_B}{U_P}[V_{120}]_B\end{aligned} \qquad \text{(S.2)}$$

$$
\begin{aligned}
\frac{d[V_{165}]_N}{dt} = & -k_{\deg,V}[V_{165}]_N - k^N_{on,V165,MEBM}[V_{165}]_N[M_{EBM}]_N + k^N_{off,V165,MEBM}[V_{165}M_{EBM}]_N \\
& -k^N_{on,V165,MPBM}[V_{165}]_N[M_{PBM}]_N + k^N_{off,V165,MPBM}[V_{165}M_{PBM}]_N \\
& -k^N_{on,V165,MECM}[V_{165}]_N[M_{ECM}]_N + k^N_{off,V165,MECM}[V_{165}M_{ECM}]_N \\
& -k^N_{on,V165,R1}[V_{165}]_N[R_1]_N + k^N_{off,V165R1}[V_{165}R_1]_N \\
& -k^N_{on,V165,R2}[V_{165}]_N[R_2]_N + k^N_{off,V165R2}[V_{165}R_2]_N \\
& -k^N_{on,V165,N1}[V_{165}]_N[N_1]_N + k^N_{off,V164N1}[V_{165}N_1]_N \\
& -k^{N,myo}_{on,V165,N1}[V_{165}]_N[N_1]_{N,myo} + k^{N,myo}_{off,V164N1}[V_{165}N_1]_{N,myo} \\
& -k^N_{on,V165,A}[V_{165}]_N[A]_N + k^N_{off,V164A}[V_{165}A]_N \\
& -\left(\frac{k_L + k^{NB}_{p,V}S_{NB}}{U_N}\right)\frac{[V_{165}]_N}{K_{AV,N}} + k^{BN}_{p,V}\frac{S_{NB}}{U_N}\frac{U_B}{U_P}[V_{165}]_B
\end{aligned}
\tag{S.3}
$$

$$
\begin{aligned}
\frac{d[V_{121}]_N}{dt} = & -k_{\deg,V}[V_{121}]_N - k^N_{on,V121,R1}[V_{121}]_N[R_1]_N + k^N_{off,V121R1}[V_{121}R_1]_N \\
& -k^N_{on,V121,R1N1}[V_{121}]_N[R_1N_1]_N + k^N_{off,V121R1N1}[V_{121}R_1N_1]_N \\
& -k^N_{on,V121,R2}[V_{12}]_N[R_2]_N + k^N_{off,V121R2}[V_{121}R_2]_N \\
& -k^N_{on,V121,A}[V_{121}]_N[A]_N + k^N_{off,V121A}[V_{121}A]_N \\
& -\left(\frac{k_L + k^{NB}_{p,V}S_{NB}}{U_N}\right)\frac{[V_{121}]_N}{K_{AV,N}} + k^{BN}_{p,V}\frac{S_{NB}}{U_N}\frac{U_B}{U_P}[V_{121}]_B
\end{aligned}
\tag{S.4}
$$

$$
\begin{aligned}
\frac{d[M_{EBM}]_N}{dt} = & -k^N_{on,V164,MEBM}[V_{164}]_N[M_{EBM}]_N + k^N_{off,V164MEBM}[V_{164}M_{EBM}]_N \\
& -k^N_{on,V165,MEBM}[V_{165}]_N[M_{EBM}]_N + k^N_{off,V165MEBM}[V_{165}M_{EBM}]_N \\
& -k^N_{on,sR1,MEBM}[sR_1]_N[M_{EBM}]_N + k^N_{off,sR1MEBM}[sR_1M_{EBM}]_N
\end{aligned}
\tag{S.5}
$$

$$
\begin{aligned}
\frac{d[M_{PBM}]_N}{dt} = & -k^N_{on,V164,MPBM}[V_{164}]_N[M_{PBM}]_N + k^N_{off,V164MPBM}[V_{164}M_{PBM}]_N \\
& -k^N_{on,V165,MPBM}[V_{165}]_N[M_{PBM}]_N + k^N_{off,V165MPBM}[V_{165}M_{PBM}]_N \\
& -k^N_{on,sR1,MPBM}[sR_1]_N[M_{PBM}]_N + k^N_{off,sR1MPBM}[sR_1M_{PBM}]_N
\end{aligned}
\tag{S.6}
$$

$$\frac{d[M_{ECM}]_N}{dt} = -k^N_{on,V164,MECM}[V_{164}]_N[M_{ECM}]_N + k^N_{off,V164MECM}[V_{164}M_{ECM}]_N$$
$$-k^N_{on,V165,MECM}[V_{165}]_N[M_{ECM}]_N + k^N_{off,V165MECM}[V_{165}M_{ECM}]_N \qquad \text{(S.7)}$$
$$-k^N_{on,sR1,MECM}[sR_1]_N[M_{ECM}]_N + k^N_{off,sR1MECM}[sR_1M_{ECM}]_N$$

$$\frac{d[V_{164}M_{EBM}]_N}{dt} = k^N_{on,V164,MEBM}[V_{164}]_N[M_{EBM}]_N - k^N_{off,V164MEBM}[V_{164}M_{EBM}]_N \qquad \text{(S.8)}$$

$$\frac{d[V_{164}M_{PBM}]_N}{dt} = k^N_{on,V164,MPBM}[V_{164}]_N[M_{PBM}]_N - k^N_{off,V164MPBM}[V_{164}M_{PBM}]_N \qquad \text{(S.9)}$$

$$\frac{d[V_{164}M_{ECM}]_N}{dt} = k^N_{on,V164,MECM}[V_{164}]_N[M_{ECM}]_N - k^N_{off,V164MECM}[V_{164}M_{ECM}]_N \qquad \text{(S.10)}$$

$$\frac{d[V_{165}M_{EBM}]_N}{dt} = k^N_{on,V165,MEBM}[V_{165}]_N[M_{EBM}]_N - k^N_{off,V165MEBM}[V_{165}M_{EBM}]_N \qquad \text{(S.11)}$$

$$\frac{d[V_{165}M_{PBM}]_N}{dt} = k^N_{on,V165,MPBM}[V_{165}]_N[M_{PBM}]_N - k^N_{off,V165MPBM}[V_{165}M_{PBM}]_N \qquad \text{(S.12)}$$

$$\frac{d[V_{165}M_{ECM}]_N}{dt} = k^N_{on,V165,MECM}[V_{165}]_N[M_{ECM}]_N - k^N_{off,V165MECM}[V_{165}M_{ECM}]_N \qquad \text{(S.13)}$$

$$\frac{d[R_1]_N}{dt} = s^N_{R1} - k^N_{int,R1}[R_1]_N - k^N_{on,V164,R1}[V_{164}]_N[R_1]_N + k^N_{off,V164R1}[V_{164}R_1]_N$$
$$-k^N_{on,V120,R1}[V_{120}]_N[R_1]_N + k^N_{off,V120R1}[V_{120}R_1]_N$$
$$-k^N_{on,V165,R1}[V_{165}]_N[R_1]_N + k^N_{off,V165R1}[V_{165}R_1]_N \qquad \text{(S.14)}$$
$$-k^N_{on,V121,R1}[V_{121}]_N[R_1]_N + k^N_{off,V121R1}[V_{121}R_1]_N$$
$$-k^N_{c,R1,N1}[N_1]_N[R_1]_N + k^N_{dissoc,R1N1}[R_1N_1]_N$$

$$\frac{d[R_2]_N}{dt} = s^N_{R2} - k^N_{int,R2}[R_2]_N - k^N_{on,V120,R2}[V_{120}]_N[R_2]_N + k^N_{off,V120R2}[V_{120}R_2]_N$$
$$-k^N_{on,V164,R2}[V_{164}]_N[R_2]_N + k^N_{off,V164R2}[V_{164}R_2]_N$$
$$-k^N_{c,V164N1,R2}[V_{164}N_1]_N[R_2]_N + k^N_{off,V164N1,R2}[R_2V_{164}N_1]_N \qquad \text{(S.15)}$$
$$-k^N_{on,V121,R2}[V_{121}]_N[R_2]_N + k^N_{off,V121R2}[V_{121}R_2]_N$$
$$-k^N_{on,V165,R2}[V_{165}]_N[R_2]_N + k^N_{off,V165R2}[V_{165}R_2]_N$$
$$-k^N_{c,V165N1,R2}[V_{165}N_1]_N[R_2]_N + k^N_{off,V165N1,R2}[R_2V_{165}N_1]_N$$

$$\frac{d[N_1]_N}{dt} = s_{N1}^N - k_{int,N1}^N[N_1]_N - k_{c,V120R1,N1}^N[V_{120}R_1]_N[N_1]_N + k_{dissoc,R1N1}^N[V_{120}R_1N_1]_N$$
$$-k_{on,V164,N1}^N[V_{164}]_N[N_1]_N + k_{off,V164N1}^N[V_{164}N_1]_N$$
$$-k_{c,V164R2,N1}^N[V_{164}R_2]_N[N_1]_N + k_{off,V164R2,N1}^N[R_2V_{164}N_1]_N$$
$$-k_{c,V121R1,N1}^N[V_{121}R_1]_N[N_1]_N + k_{dissoc,R1N1}^N[V_{121}R_1N_1]_N \quad \text{(S.16)}$$
$$-k_{on,V165,N1}^N[V_{165}]_N[N_1]_N + k_{off,V165N1}^N[V_{165}N_1]_N$$
$$-k_{c,V165R2,N1}^N[V_{165}R_2]_N[N_1]_N + k_{off,V165R2,N1}^N[R_2V_{165}N_1]_N$$
$$-k_{c,R1,N1}^N[N_1]_N[R_1]_N + k_{dissoc,R1N1}^N[R_1N_1]_N$$

$$\frac{d[N_1]_{N,myo}}{dt} = s_{N1}^{N,myo} - k_{\text{int},N1}^{N,myo}[N_1]_{N,myo}$$
$$-k_{on,V164,N1}^{N,myo}[V_{164}]_N[N_1]_{N,myo} + k_{off,V164N1}^{N,myo}[V_{164}N_1]_{N,myo} \quad \text{(S.17)}$$
$$-k_{on,V165,N1}^{N,myo}[V_{165}]_N[N_1]_{N,myo} + k_{off,V165N1}^{N,myo}[V_{165}N_1]_{N,myo}$$

$$\frac{d[V_{164}R_1]_N}{dt} = -k_{int,V164R1}^N[V_{164}R_1]_N + k_{on,V164,R1}^N[V_{164}]_N[R_1]_N - k_{off,V164R1}^N[V_{164}R_1]_N \quad \text{(S.18)}$$

$$\frac{d[V_{164}R_2]_N}{dt} = -k_{int,V164R2}^N[V_{164}R_2]_N + k_{on,V164,R2}^N[V_{164}]_N[R_2]_N - k_{off,V164R2}^N[V_{164}R_2]_N$$
$$-k_{c,V164R2,N1}^N[V_{164}R_2]_N[N_1]_N + k_{off,V164R2N1}^N[R_2V_{164}N_1]_N \quad \text{(S.19)}$$

$$\frac{d[V_{164}N_1]_N}{dt} = -k_{int,V164N1}^N[V_{164}N_1]_N + k_{on,V164,N1}^N[V_{164}]_N[N_1]_N - k_{off,V164N1}^N[V_{164}N_1]_N$$
$$-k_{c,V164N1,R2}^N[V_{164}N_1]_N[R_2]_N + k_{off,V164N1R2}^N[R_2V_{164}N_1]_N \quad \text{(S.20)}$$

$$\frac{d[R_2V_{164}N_1]_N}{dt} = -k_{int,V164R2N1}^N[R_2V_{164}N_1]_N$$
$$+k_{c,V164R2,N1}^N[V_{164}R_2]_N[N_1]_N - k_{off,V164R2N1}^N[R_2V_{164}N_1]_N$$
$$+k_{c,V164N1,R2}^N[V_{164}N_1]_N[R_2]_N - k_{off,V164N1R2}^N[R_2V_{164}N_1]_N \quad \text{(S.21)}$$

$$\frac{d[V_{120}R_1]_N}{dt} = -k_{int,V120R1}^N[V_{120}R_1]_N$$
$$+k_{on,V120,R1}^N[V_{120}]_N[R_1]_N - k_{off,V120R1}^N[V_{120}R_1]_N$$
$$-k_{c,R1,N1}^N[V_{120}R_1]_N[N_1]_N + k_{dissoc,R1N1}^N[V_{120}R_1N_1]_N \quad \text{(S.22)}$$

$$\frac{d[V_{120}R_2]_N}{dt} = -k^N_{int,V120R2}[V_{120}R_2]_N + k^N_{on,V120,R2}[V_{120}]_N[R_2]_N - k^N_{off,V120R2}[V_{120}R_2]_N \tag{S.23}$$

$$\begin{aligned}\frac{d[V_{120}R_1N_1]_N}{dt} = &-k^N_{intV120R1N1}[V_{120}R_1N_1]_N \\ &+k^N_{c,V120R1,N1}[V_{120}R_1]_N[N_1]_N - k^N_{dissoc,V120N1}[V_{120}R_1N_1]_N \\ &+k^N_{on,V120R1N1}[V_{120}]_N[R_1N_1]_N - k^N_{off,V120R1N1}[V_{120}R_1N_1]_N\end{aligned} \tag{S.24}$$

$$\begin{aligned}\frac{d[V_{164}N_1]_{N.myo}}{dt} = &-k^{N,myo}_{int,V164N1}[V_{164}N_1]_{N,myo} \\ &+k^{N,myo}_{on,V164,N1}[V_{164}]_N[N_1]_{N,myo} - k^{N,myo}_{off,V164N1}[V_{164}N_1]_{N,myo}\end{aligned} \tag{S.25}$$

$$\frac{d[V_{165}R_1]_N}{dt} = -k^N_{int,V165R1}[V_{164}R_1]_N + k^N_{on,V165,R1}[V_{165}]_N[R_1]_N - k^N_{off,V165R1}[V_{165}R_1]_N \tag{S.26}$$

$$\begin{aligned}\frac{d[V_{165}R_2]_N}{dt} = &-k^N_{int,V165R2}[V_{165}R_2]_N + k^N_{on,V165,R2}[V_{165}]_N[R_2]_N - k^N_{off,V165R2}[V_{165}R_2]_N \\ &-k^N_{c,V165R2,N1}[V_{165}R_2]_N[N_1]_N + k^N_{off,V165R2N1}[R_2V_{165}N_1]_N\end{aligned} \tag{S.27}$$

$$\begin{aligned}\frac{d[V_{165}N_1]_N}{dt} = &-k^N_{int,V165N1}[V_{165}N_1]_N + k^N_{on,V165,N1}[V_{165}]_N[N_1]_N - k^N_{off,V165N1}[V_{165}N_1]_N \\ &-k^N_{c,V165N1,R2}[V_{165}N_1]_N[R_2]_N + k^N_{off,V165N1R2}[R_2V_{165}N_1]_N\end{aligned} \tag{S.28}$$

$$\begin{aligned}\frac{d[R_2V_{165}N_1]_N}{dt} = &-k^N_{int,V165R2N1}[R_2V_{165}N_1]_N \\ &+k^N_{c,V165R2,N1}[V_{165}R_2]_N[N_1]_N - k^N_{off,V165R2N1}[R_2V_{165}N_1]_N \\ &+k^N_{c,V165N1,R2}[V_{165}N_1]_N[R_2]_N - k^N_{off,V165N1R2}[R_2V_{165}N_1]_N\end{aligned} \tag{S.29}$$

$$\begin{aligned}\frac{d[V_{121}R_1]_N}{dt} = &-k^N_{int,V121R1}[V_{121}R_1]_N \\ &+k^N_{on,V121,R1}[V_{121}]_N[R_1]_N - k^N_{off,V121R1}[V_{121}R_1]_N \\ &-k^N_{c,R1,N1}[V_{121}R_1]_N[N_1]_N + k^N_{dissoc,R1N1}[V_{121}R_1N_1]_N\end{aligned} \tag{S.30}$$

$$\frac{d[V_{121}R_2]_N}{dt} = -k^N_{int,V121R2}[V_{121}R_2]_N + k^N_{on,V121,R2}[V_{121}]_N[R_2]_N - k^N_{off,V121R2}[V_{121}R_2]_N \tag{S.31}$$

$$\frac{d[V_{121}R_1N_1]_N}{dt} = -k^N_{intV121R1N1}[V_{121}R_1N_1]_N$$
$$+k^N_{c,V121R1,N1}[V_{121}R_1]_N[N_1]_N - k^N_{dissoc,V121N1}[V_{121}R_1N_1]_N$$
$$+k^N_{on,V121R1N1}[V_{121}]_N[R_1N_1]_N - k^N_{off,V121R1N1}[V_{121}R_1N_1]_N \tag{S.32}$$

$$\frac{d[V_{165}N_1]_{N.myo}}{dt} = -k^{N,myo}_{int,V165N1}[V_{165}N_1]_{N,myo}$$
$$+k^{N,myo}_{on,V165,N1}[V_{165}]_N[N_1]_{N,myo} - k^{N,myo}_{off,V165N1}[V_{165}N_1]_{N,myo} \tag{S.33}$$

$$\frac{d[R_1N_1]_N}{dt} = -k^N_{int,R1N1}[R_1N_1]_N$$
$$+k^N_{c,R1,N1}[R_1]_N[N_1]_N - k^N_{dissoc,R1N1}[R_1N_1]_N \tag{S.34}$$
$$-k^N_{on,V120,R1}[V_{120}]_N[R_1N_1]_N + k^N_{off,V120R1}[V_{120}R_1N_1]_N$$
$$-k^N_{on,V121,R1}[V_{121}]_N[R_1N_1]_N + k^N_{off,V121R1}[V_{121}R_1N_1]_N$$

$$\frac{d[A]_N}{dt} = -k^N_{on,V164,A}[V_{164}]_N[A]_N + k^N_{off,V164A}[V_{164}A]_N$$
$$-k^N_{on,V120,A}[V_{120}]_N[A]_N + k^N_{off,V120A}[V_{120}A]_N$$
$$-k^N_{on,V165,A}[V_{165}]_N[A]_N + k^N_{off,V165A}[V_{165}A]_N \tag{S.35}$$
$$-k^N_{on,V121,A}[V_{121}]_N[A]_N + k^N_{off,V121A}[V_{121}A]_N$$
$$+k^{BN}_{p,A}\frac{S_{NB}}{U_N}\frac{U_B}{U_P}[A]_B - \left(\frac{k_L + k^{NB}_{p,A}S_{NB}}{U_N}\right)\frac{[A]_N}{K_{AV,N}}$$

$$\frac{d[V_{164}A]_N}{dt} = k^N_{on,V164,A}[V_{164}]_N[A]_N - k^N_{off,V164A}[V_{164}A]_N$$
$$+k^{BN}_{p,A}\frac{S_{NB}}{U_N}\frac{U_B}{U_p}[V_{164}A]_B - \left(\frac{k_L + k^{NB}_{p,A}S_{NB}}{U_N}\right)\frac{[V_{164}A]_N}{K_{AV,N}} \tag{S.36}$$

$$\frac{d[V_{120}A]_N}{dt} = k^N_{on,V120,A}[V_{120}]_N[A]_N - k^N_{off,V120A}[V_{120}A]_N$$
$$+k^{BN}_{p,A}\frac{S_{NB}}{U_N}\frac{U_B}{U_p}[V_{120}A]_B - \left(\frac{k_L + k^{NB}_{p,A}S_{NB}}{U_N}\right)\frac{[V_{120}A]_N}{K_{AV,N}} \tag{S.37}$$

$$\frac{d[V_{165}A]_N}{dt} = k^N_{on,V165,A}[V_{165}]_N[A]_N - k^N_{off,V165A}[V_{165}A]_N + k^{BN}_{p,A}\frac{S_{NB}}{U_N}\frac{U_B}{U_p}[V_{165}A]_B - \left(\frac{k_L + k^{NB}_{p,A}S_{NB}}{U_N}\right)\frac{[V_{165}A]_N}{K_{AV,N}} \tag{S.38}$$

$$\frac{d[V_{121}A]_N}{dt} = k^N_{on,V121,A}[V_{121}]_N[A]_N - k^N_{off,V121A}[V_{121}A]_N + k^{BN}_{p,A}\frac{S_{NB}}{U_N}\frac{U_B}{U_p}[V_{121}A]_B - \left(\frac{k_L + k^{NB}_{p,A}S_{NB}}{U_N}\right)\frac{[V_{121}A]_N}{K_{AV,N}} \tag{S.39}$$

$$\begin{aligned}\frac{d[sR_1]_N}{dt} = {} & q^N_{sR1} - k_{\deg,sR1}[sR_1]_N - k^N_{on,sR1,MEBM}[sR_1]_N[M_{EBM}]_N + k^N_{off,sR1,MEBM}[sR_1M_{EBM}]_N \\ & - k^N_{on,sR1,MPBM}[sR_1]_N[M_{PBM}]_N + k^N_{off,sR1,MPBM}[sR_1M_{PBM}]_N \\ & - k^N_{on,sR1,MECM}[sR_1]_N[M_{ECM}]_N + k^N_{off,sR1,MECM}[sR_1M_{ECM}]_N \\ & - k^N_{on,V164,sR1}[V_{164}]_N[sR_1]_N + k^N_{off,V164sR1}[V_{164}sR_1]_N \\ & - k^N_{on,V165,sR1}[V_{165}]_N[sR_1]_N + k^N_{off,V165sR1}[V_{165}sR_1]_N \\ & - k^N_{on,V120,sR1}[V_{120}]_N[sR_1]_N + k^N_{off,V120sR1}[V_{120}sR_1]_N \\ & - k^N_{on,V121,sR1}[V_{121}]_N[sR_1]_N + k^N_{off,V121sR1}[V_{121}sR_1]_N \\ & - k^N_{on,sR1,N1}[sR_1]_N[N_1]_N + k^N_{off,sR1N1}[sR_1N_1]_N \\ & - k^{N,myo}_{on,sR1,N1}[sR_1]_N[N_1]_{N,myo} + k^{N,myo}_{off,sR1N1}[sR_1N_1]_{N,myo} \\ & - \left(\frac{k_L + k^{NB}_{p,sR1}S_{NB}}{U_N}\right)\frac{[sR_1]_N}{K_{AV,N}} + k^{BN}_{p,sR1}\frac{S_{NB}}{U_N}\frac{U_B}{U_P}[sR_1]_B\end{aligned} \tag{S.40}$$

$$\frac{d[sR_1M_{EBM}]_N}{dt} = k^N_{on,sR1,MEBM}[sR_1]_N[M_{EBM}]_N - k^N_{off,sR1MEBM}[sR_1M_{EBM}]_N \tag{S.41}$$

$$\frac{d[sR_1M_{PBM}]_N}{dt} = k^N_{on,sR1,MPBM}[sR_1]_N[M_{PBM}]_N - k^N_{off,sR1MPBM}[sR_1M_{PBM}]_N \tag{S.42}$$

$$\frac{d[sR_1M_{ECM}]_N}{dt} = k^N_{on,sR1,MECM}[sR_1]_N[M_{ECM}]_N - k^N_{off,sR1MECM}[sR_1M_{ECM}]_N \tag{S.43}$$

$$\begin{aligned}\frac{d[sR_1N_1]_N}{dt} = {} & -k^N_{int,sR1N1}[sR_1N_1]_N + k^N_{on,sR1,N1}[sR_1]_N[N_1]_N - k^N_{off,sR1N1}[sR_1N_1]_N \\ & - k^N_{on,V120,sR1N1}[V_{120}]_N[sR_1N_1]_N + k^N_{off,V120,sR1N1}[V_{120}sR_1N_1]_N \\ & - k^N_{on,V121,sR1N1}[V_{121}]_N[sR_1N_1]_N + k^N_{off,V121,sR1N1}[V_{121}sR_1N_1]_N\end{aligned} \tag{S.44}$$

$$\frac{d[sR_1N_1]_{N,myo}}{dt} = -k^{N,myo}_{int,sR1N1}[sR_1N_1]_{N,myo} + k^{N,myo}_{on,sR1,N1}[sR_1]_N[N_1]_{N,myo} - k^{N,myo}_{off,sR1N1}[sR_1N_1]_{N,myo}$$
$$-k^{N,myo}_{on,V120,sR1N1}[V_{120}]_N[sR_1N_1]_{N,myo} + k^{N,myo}_{off,V120,sR1N1}[V_{120}sR_1N_1]_{N,myo} \quad \text{(S.45)}$$
$$-k^{N,myo}_{on,V121,sR1N1}[V_{121}]_N[sR_1N_1]_{N,myo} + k^{N,myo}_{off,V121,sR1N1}[V_{121}sR_1N_1]_{N,myo}$$

$$\frac{d[V_{164}sR_1]_N}{dt} = -k_{\deg,VsR1}[V_{164}sR_1]_N + k^N_{on,V164,sR1}[V_{164}]_N[sR_1]_N - k^N_{off,V164sR1}[V_{164}sR_1]_N$$
$$-\left(\frac{k_L + k^{NB}_{p,VsR1}S_{NB}}{U_N}\right)\frac{[V_{164}sR_1]_N}{K_{AV,N}} + k^{BN}_{p,VsR1}\frac{S_{NB}}{U_N}\frac{U_B}{U_P}[V_{164}sR_1]_B \quad \text{(S.46)}$$

$$\frac{d[V_{120}sR_1]_N}{dt} = -k_{\deg,VsR1}[V_{120}sR_1]_N$$
$$+k^N_{on,V120,sR1}[V_{120}]_N[sR_1]_N - k^N_{off,V120sR1}[V_{120}sR_1]_N$$
$$-k^N_{on,sR1,N1}[V_{120}sR_1]_N[N_1]_N + k^N_{off,R1sN1}[V_{120}sR_1N_1]_N \quad \text{(S.47)}$$
$$-\left(\frac{k_L + k^{NB}_{pVsR1}S_{NB}}{U_N}\right)\frac{[V_{120}sR_1]_N}{K_{AV,N}} + k^{BN}_{pVsR1}\frac{S_{NB}}{U_N}\frac{U_B}{U_P}[V_{120}sR_1]_B$$

$$\frac{d[V_{120}sR_1N_1]_N}{dt} = -k^N_{intV120sR1N1}[V_{120}sR_1N_1]_N$$
$$+k^N_{on,sR1,N1}[V_{120}R_1]_N[N_1]_N - k^N_{off,sR1N1}[V_{120}sR_1N_1]_N \quad \text{(S.48)}$$
$$+k^N_{on,V120sR1N1}[V_{120}]_N[R_1N_1]_N - k^N_{off,V120sR1N1}[V_{120}sR_1N_1]_N$$

$$\frac{d[V_{120}sR_1N_1]_{N,myo}}{dt} = -k^{N,myo}_{intV120sR1N1}[V_{120}sR_1N_1]_{N,myo}$$
$$+k^{N,myo}_{on,sR1,N1}[V_{120}R_1]_N[N_1]_{N,myo} - k^{N,myo}_{off,sR1N1}[V_{120}sR_1N_1]_{N,myo} \quad \text{(S.49)}$$
$$+k^{N,myo}_{on,V120sR1N1}[V_{120}]_N[R_1N_1]_{N,myo} - k^{N,myo}_{off,V120sR1N1}[V_{120}sR_1N_1]_{N,myo}$$

$$\frac{d[V_{165}sR_1]_N}{dt} = -k_{\deg,VsR1}[V_{165}sR_1]_N + k^N_{on,V165,sR1}[V_{165}]_N[sR_1]_N - k^N_{off,V165sR1}[V_{165}sR_1]_N$$
$$-\left(\frac{k_L + k^{NB}_{p,VsR1}S_{NB}}{U_N}\right)\frac{[V_{165}sR_1]_N}{K_{AV,N}} + k^{BN}_{p,VsR1}\frac{S_{NB}}{U_N}\frac{U_B}{U_P}[V_{165}sR_1]_B \quad \text{(S.50)}$$

$$\frac{d[V_{121}sR_1]_N}{dt} = -k_{\deg,VsR1}[V_{121}sR_1]_N + k^N_{on,V121,sR1}[V_{121}]_N[sR_1]_N - k^N_{off,V121sR1}[V_{121}sR_1]_N - k^N_{on,sR1,N1}[V_{121}sR_1]_N[N_1]_N + k^N_{off,R1sN1}[V_{121}sR_1N_1]_N - \left(\frac{k_L + k^{NB}_{p,VsR1}S_{NB}}{U_N}\right)\frac{[V_{121}sR_1]_N}{K_{AV,N}} + k^{BN}_{p,VsR1}\frac{S_{NB}}{U_N}\frac{U_B}{U_P}[V_{121}sR_1]_B \tag{S.51}$$

$$\frac{d[V_{121}sR_1N_1]_N}{dt} = -k^N_{intV121sR1N1}[V_{121}sR_1N_1]_N + k^N_{on,sR1,N1}[V_{121}R_1]_N[N_1]_N - k^N_{off,sR1N1}[V_{121}sR_1N_1]_N + k^N_{on,V121sR1N1}[V_{121}]_N[R_1N_1]_N - k^N_{off,V121sR1N1}[V_{121}sR_1N_1]_N \tag{S.52}$$

$$\frac{d[V_{121}sR_1N_1]_{N,myo}}{dt} = -k^{N,myo}_{intV121sR1N1}[V_{121}sR_1N_1]_{N,myo} + k^{N,myo}_{on,sR1,N1}[V_{121}R_1]_N[N_1]_{N,myo} - k^{N,myo}_{off,sR1N1}[V_{121}sR_1N_1]_{N,myo} + k^{N,myo}_{on,V121sR1N1}[V_{121}]_N[R_1N_1]_{N,myo} - k^{N,myo}_{off,V121sR1N1}[V_{121}sR_1N_1]_{N,myo} \tag{S.53}$$

B. Blood compartment

We denote the luminal receptors and ligand-receptor complexes on endothelial cells (ECs) by the subscript i (i=N for normal ECs; i=T for diseased ECs).

$$
\begin{aligned}
\frac{d[V_{164}]_B}{dt} &= q_{V164}^B - c_{V164}[V_{164}]_B - k_{on,V164,R1}^B[V_{164}]_B[R_1]_{B,i} + k_{off,V164R1}^B[V_{164}R_1]_{B,i} \\
&-k_{on,V164,R2}^B[V_{164}]_B[R_2]_{B,i} + k_{off,V164R2}^B[V_{164}R_2]_{B,i} \\
&-k_{on,V164,N1}^B[V_{164}]_B[N_1]_{B,i} + k_{off,V164N1}^B[V_{164}N_1]_{B,i} \\
&-k_{on,V164,A}^B[V_{164}]_B[A]_B + k_{off,V164A}^B[V_{164}A]_B \\
&-k_{on,V164,\alpha 2\mathrm{M}}^B[V_{164}]_B[\alpha 2\mathrm{M}]_B + k_{off,V164\alpha 2\mathrm{M}}^B[\alpha 2\mathrm{M}\cdot V_{164}]_B \\
&-k_{on,V164,\alpha 2\mathrm{M}fast}^B[V_{164}]_B[\alpha 2\mathrm{M}_{fast}]_B + k_{off,V164\alpha 2\mathrm{M}fast}^B[\alpha 2\mathrm{M}_{fast}\cdot V_{164}]_B \\
&-\frac{k_{p,V}^{BN}S_{NB}}{U_p}[V_{164}]_B + \left(\frac{k_L + k_{p,V}^{NB}S_{NB}}{U_B}\right)\frac{[V_{164}]_N}{K_{AV,N}} \\
&-\frac{k_{p,V}^{BT}S_{TB}}{U_p}[V_{164}]_B + \left(\frac{k_{p,V}^{TB}S_{TB}}{U_B}\right)\frac{[V_{164}]_T}{K_{AV,T}}
\end{aligned}
\tag{S.54}
$$

$$
\begin{aligned}
\frac{d[V_{120}]_B}{dt} &= q_{V120}^B - c_{V120}[V_{120}]_B - k_{on,V120,R1}^B[V_{120}]_B[R_1]_{B,i} + k_{off,V120R1}^B[V_{120}R_1]_{B,i} \\
&-k_{on,V120,R1N1}^B[V_{120}]_B[R_1N_1]_{B,i} + k_{off,V120R1N1}^B[V_{120}R_1N_1]_{B,i} \\
&-k_{on,V120,R2}^B[V_{120}]_B[R_2]_{B,i} + k_{off,V120R2}^B[V_{120}R_2]_{B,i} \\
&-k_{on,V120,A}^B[V_{120}]_B[A]_B + k_{off,V120A}^B[V_{120}A]_B \\
&-k_{on,V120,\alpha 2\mathrm{M}}^B[V_{120}]_B[\alpha 2\mathrm{M}]_B + k_{off,V120\alpha 2\mathrm{M}}^B[\alpha 2\mathrm{M}\cdot V_{120}A]_B \\
&-k_{on,V120,\alpha 2\mathrm{M}fast}^B[V_{120}]_B[\alpha 2\mathrm{M}_{fast}]_B + k_{off,V120\alpha 2\mathrm{M}fast}^B[\alpha 2\mathrm{M}_{fast}\cdot V_{120}A]_B \\
&-\frac{k_{p,V}^{BN}S_{NB}}{U_p}[V_{120}]_B + \left(\frac{k_L + k_{p,V}^{NB}S_{NB}}{U_B}\right)\frac{[V_{120}]_N}{K_{AV,N}} \\
&-\frac{k_{p,V}^{BT}S_{TB}}{U_p}[V_{120}]_B + \left(\frac{k_{p,V}^{TB}S_{TB}}{U_B}\right)\frac{[V_{120}]_T}{K_{AV,T}}
\end{aligned}
\tag{S.55}
$$

$$
\begin{aligned}
\frac{d[V_{165}]_B}{dt} = {} & q^B_{V165} - c_{V165}[V_{165}]_B - k^B_{on,V165,R1}[V_{165}]_B[R_1]_{B,i} + k^B_{off,V165R1}[V_{165}R_1]_{B,i} \\
& - k^B_{on,V165,R2}[V_{165}]_B[R_2]_{B,i} + k^B_{off,V165R2}[V_{165}R_2]_{B,i} \\
& - k^B_{on,V165,N1}[V_{165}]_B[N_1]_{B,i} + k^B_{off,V165N1}[V_{165}N_1]_{B,i} \\
& - k^B_{on,V165,A}[V_{165}]_B[A]_B + k^B_{off,V165A}[V_{165}A]_B \\
& - k^B_{on,V165,\alpha 2\mathrm{M}}[V_{165}]_B[\alpha 2\mathrm{M}]_B + k^B_{off,V165\alpha 2\mathrm{M}}[\alpha 2\mathrm{M}\cdot V_{165}]_B \\
& - k^B_{on,V165,\alpha 2\mathrm{M}fast}[V_{165}]_B[\alpha 2\mathrm{M}_{fast}]_B + k^B_{off,V165\alpha 2\mathrm{M}fast}[\alpha 2\mathrm{M}_{fast}\cdot V_{165}]_B \\
& - \frac{k^{BN}_{p,V}S_{NB}}{U_p}[V_{165}]_B + \left(\frac{k_L + k^{NB}_{p,V}S_{NB}}{U_B}\right)\frac{[V_{165}]_N}{K_{AV,N}} \\
& - \frac{k^{BT}_{p,V}S_{TB}}{U_p}[V_{165}]_B + \left(\frac{k^{TB}_{p,V}S_{TB}}{U_B}\right)\frac{[V_{165}]_T}{K_{AV,T}}
\end{aligned}
\qquad \text{(S.56)}
$$

$$
\begin{aligned}
\frac{d[V_{121}]_B}{dt} = {} & q^B_{V121} - c_{V121}[V_{121}]_B - k^B_{on,V121,R1}[V_{121}]_B[R_1]_{B,i} + k^B_{off,V121R1}[V_{121}R_1]_{B,i} \\
& - k^B_{on,V121,R1N1}[V_{121}]_B[R_1N_1]_{B,i} + k^B_{off,V121R1N1}[V_{121}R_1N_1]_{B,i} \\
& - k^B_{on,V121,R2}[V_{121}]_B[R_2]_{B,i} + k^B_{off,V121R2}[V_{121}R_2]_{B,i} \\
& - k^B_{on,V121,A}[V_{121}]_B[A]_B + k^B_{off,V121A}[V_{121}A]_B \\
& - k^B_{on,V121,\alpha 2\mathrm{M}}[V_{121}]_B[\alpha 2\mathrm{M}]_B + k^B_{off,V121\alpha 2\mathrm{M}}[\alpha 2\mathrm{M}\cdot V_{121}A]_B \\
& - k^B_{on,V121,\alpha 2\mathrm{M}fast}[V_{121}]_B[\alpha 2\mathrm{M}_{fast}]_B + k^B_{off,V121\alpha 2\mathrm{M}fast}[\alpha 2\mathrm{M}_{fast}\cdot V_{121}A]_B \\
& - \frac{k^{BN}_{p,V}S_{NB}}{U_p}[V_{121}]_B + \left(\frac{k_L + k^{NB}_{p,V}S_{NB}}{U_B}\right)\frac{[V_{121}]_N}{K_{AV,N}} \\
& - \frac{k^{BT}_{p,V}S_{TB}}{U_p}[V_{121}]_B + \left(\frac{k^{TB}_{p,V}S_{TB}}{U_B}\right)\frac{[V_{121}]_T}{K_{AV,T}}
\end{aligned}
\qquad \text{(S.57)}
$$

$$
\begin{aligned}
\frac{d[R_1]_{B,i}}{dt} = {} & s_{R1}^B - k_{int,R1}^B [R_1]_{B,i} - k_{on,V164,R1}^B\ [V_{164}]_B [R_1]_{B,i} + k_{off,V164R1}^B [V_{164}R_1]_{B,i} \\
& - k_{on,V120,R1}^B [V_{120}]_B [R_1]_{B,i} + k_{off,V120R1}^B [V_{120}R_1]_{B,i} \\
& - k_{on,V165,R1}^B\ [V_{164}]_B [R_1]_{B,i} + k_{off,V165R1}^B [V_{165}R_1]_{B,i} \\
& - k_{on,V121,R1}^B [V_{121}]_B [R_1]_{B,i} + k_{off,V121R1}^B [V_{121}R_1]_{B,i} \\
& - k_{c,R1,N1}^B [N_1]_{B,i} [R_1]_{B,i} + k_{dissoc,R1N1}^B [R_N N_1]_{B,i} \\
& - k_{on,V164,R1}^B\ [\alpha 2\mathrm{M} \cdot V_{164}]_B [R_1]_{B,i} + k_{off,V164R1}^B [\alpha 2\mathrm{M} \cdot V_{164}R_1]_{B,i} \\
& - k_{on,V120,R1}^B [\alpha 2\mathrm{M} \cdot V_{120}]_B [R_1]_{B,i} + k_{off,V120R1}^B [\alpha 2\mathrm{M} \cdot V_{120}R_1]_{B,i} \\
& - k_{on,V165,R1}^B\ [\alpha 2\mathrm{M} \cdot V_{165}]_B [R_1]_{B,i} + k_{off,V165R1}^B [\alpha 2\mathrm{M} \cdot V_{165}R_1]_{B,i} \\
& - k_{on,V121,R1}^B [\alpha 2\mathrm{M} \cdot V_{121}]_B [R_1]_{B,i} + k_{off,V121R1}^B [\alpha 2\mathrm{M} \cdot V_{121}R_1]_{B,i} \\
& - k_{c,R1,N1}^B [\alpha 2\mathrm{M} \cdot N_1]_{B,i} [R_1]_{B,i} + k_{dissoc,R1N1}^B [\alpha 2\mathrm{M} \cdot R_N N_1]_{B,i} \\
& - k_{on,V164,R1}^B\ [\alpha 2\mathrm{M}_{fast} \cdot V_{164}]_B [R_1]_{B,i} + k_{off,V164R1}^B [\alpha 2\mathrm{M}_{fast} \cdot V_{164}R_1]_{B,i} \\
& - k_{on,V120,R1}^B [\alpha 2\mathrm{M}_{fast} \cdot V_{120}]_B [R_1]_{B,i} + k_{off,V120R1}^B [\alpha 2\mathrm{M}_{fast} \cdot V_{120}R_1]_{B,i} \\
& - k_{on,V165,R1}^B\ [\alpha 2\mathrm{M}_{fast} \cdot V_{165}]_B [R_1]_{B,i} + k_{off,V165R1}^B [\alpha 2\mathrm{M}_{fast} \cdot V_{165}R_1]_{B,i} \\
& - k_{on,V121,R1}^B [\alpha 2\mathrm{M}_{fast} \cdot V_{121}]_B [R_1]_{B,i} + k_{off,V121R1}^B [\alpha 2\mathrm{M}_{fast} \cdot V_{121}R_1]_{B,i} \\
& - k_{c,R1,N1}^B [\alpha 2\mathrm{M}_{fast} \cdot N_1]_{B,i} [R_1]_{B,i} + k_{dissoc,R1N1}^B [\alpha 2\mathrm{M}_{fast} \cdot R_N N_1]_{B,i}
\end{aligned}
\qquad \text{(S.58, S.59)}
$$

$$
\begin{aligned}
\frac{d[R_2]_{B,i}}{dt} = {} & s_{R2}^{B} - k_{int,R2}^{B}\ [R_2]_{B,i} - k_{on,V120,R2}^{B}[V_{120}]_B[R_2]_{B,i} + k_{off,V120R2}^{B}[V_{120}R_2]_{B,i} \\
& -k_{on,V164,R2}^{B}[V_{164}]_B[R_2]_{B,i} + k_{off,V164R2}^{B}[V_{164}R_2]_{B,i} \\
& -k_{c,V164N1,R2}^{B}[V_{164}N_1]_B[R_2]_{B,i} + k_{off,V164N1,R2}^{B}[R_2V_{164}N_1]_{B,i} \\
& -k_{on,V121,R2}^{B}[V_{121}]_B[R_2]_{B,i} + k_{off,V121R2}^{B}[V_{121}R_2]_{B,i} \\
& -k_{on,V165,R2}^{B}[V_{165}]_B[R_2]_{B,i} + k_{off,V165R2}^{B}[V_{165}R_2]_{B,i} \\
& -k_{c,V165N1,R2}^{B}[V_{165}N_1]_B[R_2]_{B,i} + k_{off,V165N1,R2}^{B}[R_2V_{165}N_1]_{B,i} \\
& -k_{on,V120,R2}^{B}[\alpha 2\mathrm{M}\cdot V_{120}]_B[R_2]_{B,i} + k_{off,V120R2}^{B}[\alpha 2\mathrm{M}\cdot V_{120}R_2]_{B,i} \\
& -k_{on,V164,R2}^{B}[\alpha 2\mathrm{M}\cdot V_{164}]_B[R_2]_{B,i} + k_{off,V164R2}^{B}[\alpha 2\mathrm{M}\cdot V_{164}R_2]_{B,i} \\
& -k_{c,V164N1,R2}^{B}[\alpha 2\mathrm{M}\cdot V_{164}N_1]_B[R_2]_{B,i} + k_{off,V164N1,R2}^{B}[\alpha 2\mathrm{M}\cdot R_2V_{164}N_1]_{B,i} \\
& -k_{on,V121,R2}^{B}[\alpha 2\mathrm{M}\cdot V_{121}]_B[R_2]_{B,i} + k_{off,V121R2}^{B}[\alpha 2\mathrm{M}\cdot V_{121}R_2]_{B,i} \\
& -k_{on,V165,R2}^{B}[\alpha 2\mathrm{M}\cdot V_{165}]_B[R_2]_{B,i} + k_{off,V165R2}^{B}[\alpha 2\mathrm{M}\cdot V_{165}R_2]_{B,i} \\
& -k_{c,V165N1,R2}^{B}[\alpha 2\mathrm{M}\cdot V_{165}N_1]_B[R_2]_{B,i} + k_{off,V165N1,R2}^{B}[\alpha 2\mathrm{M}\cdot R_2V_{165}N_1]_{B,i} \\
& -k_{on,V120,R2}^{B}[\alpha 2\mathrm{M}_{fast}\cdot V_{120}]_B[R_2]_{B,i} + k_{off,V120R2}^{B}[\alpha 2\mathrm{M}_{fast}\cdot V_{120}R_2]_{B,i} \\
& -k_{on,V164,R2}^{B}[\alpha 2\mathrm{M}_{fast}\cdot V_{164}]_B[R_2]_{B,i} + k_{off,V164R2}^{B}[\alpha 2\mathrm{M}_{fast}\cdot V_{164}R_2]_{B,i} \\
& -k_{c,V164N1,R2}^{B}[\alpha 2\mathrm{M}_{fast}\cdot V_{164}N_1]_B[R_2]_{B,i} + k_{off,V164N1,R2}^{B}[\alpha 2\mathrm{M}_{fast}\cdot R_2V_{164}N_1]_{B,i} \qquad \text{(S.60, S.61)} \\
& -k_{on,V121,R2}^{B}[\alpha 2\mathrm{M}_{fast}\cdot V_{121}]_B[R_2]_{B,i} + k_{off,V121R2}^{B}[\alpha 2\mathrm{M}_{fast}\cdot V_{121}R_2]_{B,i} \\
& -k_{on,V165,R2}^{B}[\alpha 2\mathrm{M}_{fast}\cdot V_{165}]_B[R_2]_{B,i} + k_{off,V165R2}^{B}[\alpha 2\mathrm{M}_{fast}\cdot V_{165}R_2]_{B,i} \\
& -k_{c,V165N1,R2}^{B}[\alpha 2\mathrm{M}_{fast}\cdot V_{165}N_1]_B[R_2]_{B,i} + k_{off,V165N1,R2}^{B}[\alpha 2\mathrm{M}_{fast}\cdot R_2V_{165}N_1]_{B,i}
\end{aligned}
$$

$$
\begin{aligned}
\frac{d[N_1]_{B,i}}{dt} = {} & s_{N1}^B - k_{int,N1}^B[N_1]_{B,i} - k_{c,V120R1,N1}^B[V_{120}R_1]_B[N_1]_{B,i} + k_{dissoc,R1N1}^B[V_{120}R_1N_1]_{B,i} \\
& -k_{on,V164,N1}^B[V_{164}]_B[N_1]_{B,i} + k_{off,V164N1}^B[V_{164}N_1]_{B,i} \\
& -k_{c,V164R2,N1}^B[V_{164}R_2]_{B,i}[N_1]_{B,i} + k_{off,V164R2,N1}^B[R_2V_{164}N_1]_{B,i} \\
& -k_{c,V121R1,N1}^B[V_{121}R_1]_B[N_1]_{B,i} + k_{dissoc,R1N1}^B[V_{121}R_1N_1]_{B,i} \\
& -k_{on,V165,N1}^B[V_{165}]_B[N_1]_{B,i} + k_{off,V165N1}^B[V_{165}N_1]_{B,i} \\
& -k_{c,V165R2,N1}^B[V_{165}R_2]_{B,i}[N_1]_{B,i} + k_{off,V165R2,N1}^B[R_2V_{165}N_1]_{B,i} \\
& -k_{c,R1,N1}^B[N_1]_{B,i}[R_1]_{B,i} + k_{dissoc,R1N1}^B[R_1N_1]_{B,i} \\
& -k_{c,V120R1,N1}^B[\alpha 2\mathrm{M}\cdot V_{120}R_1]_B[N_1]_{B,i} + k_{dissoc,R1N1}^B[\alpha 2\mathrm{M}\cdot V_{120}R_1N_1]_{B,i} \\
& -k_{on,V164,N1}^B[\alpha 2\mathrm{M}\cdot V_{164}]_B[N_1]_{B,i} + k_{off,V164N1}^B[\alpha 2\mathrm{M}\cdot V_{164}N_1]_{B,i} \\
& -k_{c,V164R2,N1}^B[\alpha 2\mathrm{M}\cdot V_{164}R_2]_{B,i}[N_1]_{B,i} + k_{off,V164R2,N1}^B[\alpha 2\mathrm{M}\cdot R_2V_{164}N_1]_{B,i} \\
& -k_{c,V121R1,N1}^B[\alpha 2\mathrm{M}\cdot V_{121}R_1]_B[N_1]_{B,i} + k_{dissoc,R1N1}^B[\alpha 2\mathrm{M}\cdot V_{121}R_1N_1]_{B,i} \\
& -k_{on,V165,N1}^B[\alpha 2\mathrm{M}\cdot V_{165}]_B[N_1]_{B,i} + k_{off,V165N1}^B[\alpha 2\mathrm{M}\cdot V_{165}N_1]_{B,i} \\
& -k_{c,V165R2,N1}^B[\alpha 2\mathrm{M}\cdot V_{165}R_2]_{B,i}[N_1]_{B,i} + k_{off,V165R2,N1}^B[\alpha 2\mathrm{M}\cdot R_2V_{165}N_1]_{B,i} \\
& -k_{c,V120R1,N1}^B[\alpha 2\mathrm{M}_{fast}\cdot V_{120}R_1]_B[N_1]_{B,i} + k_{dissoc,R1N1}^B[\alpha 2\mathrm{M}_{fast}\cdot V_{120}R_1N_1]_{B,i} \\
& -k_{on,V164,N1}^B[\alpha 2\mathrm{M}_{fast}\cdot V_{164}]_B[N_1]_{B,i} + k_{off,V164N1}^B[\alpha 2\mathrm{M}_{fast}\cdot V_{164}N_1]_{B,i} \\
& -k_{c,V164R2,N1}^B[\alpha 2\mathrm{M}_{fast}\cdot V_{164}R_2]_{B,i}[N_1]_{B,i} + k_{off,V164R2,N1}^B[\alpha 2\mathrm{M}_{fast}\cdot R_2V_{164}N_1]_{B,i} \qquad \text{(S.62, S.63)} \\
& -k_{c,V121R1,N1}^B[\alpha 2\mathrm{M}_{fast}\cdot V_{121}R_1]_B[N_1]_{B,i} + k_{dissoc,R1N1}^B[\alpha 2\mathrm{M}_{fast}\cdot V_{121}R_1N_1]_{B,i} \\
& -k_{on,V165,N1}^B[\alpha 2\mathrm{M}_{fast}\cdot V_{165}]_B[N_1]_{B,i} + k_{off,V165N1}^B[\alpha 2\mathrm{M}_{fast}\cdot V_{165}N_1]_{B,i} \\
& -k_{c,V165R2,N1}^B[\alpha 2\mathrm{M}_{fast}\cdot V_{165}R_2]_{B,i}[N_1]_{B,i} + k_{off,V165R2,N1}^B[\alpha 2\mathrm{M}_{fast}\cdot R_2V_{165}N_1]_{B,i}
\end{aligned}
$$

$$
\frac{d[V_{164}R_1]_{B,i}}{dt} = -k_{int,V164R1}^B[V_{164}R_1]_{B,i} + k_{on,V164,R1}^B[V_{164}]_B[R_1]_{B,i} - k_{off,V164R1}^B[V_{164}R_1]_{B,i} \qquad \text{(S.64, S.65)}
$$

$$
\frac{d[V_{164}R_2]_{B,i}}{dt} = -k_{int,V164R2}^B[V_{164}R_2]_{B,i} + k_{on,V164,R2}^B[V_{164}]_B[R_2]_{B,i} - k_{off,V164R2}^B[V_{164}R_2]_{B,i} \qquad \text{(S.66, S.67)}
$$

$$
\begin{aligned}
\frac{d[V_{164}N_1]_{B,i}}{dt} = {} & -k_{int,V164N1}^B[V_{164}N_1]_{B,i} + k_{on,V164,N1}^B[V_{164}]_B[N_1]_{B,i} - k_{off,V164N1}^B[V_{164}N_1]_{B,i} \\
& -k_{c,V164N1,R2}^B[V_{164}N_1]_{B,i}[R_2]_{B,i} + k_{off,V164N1R2}^B[R_2V_{164}N_1]_{B,i} \qquad \text{(S.68, S.69)}
\end{aligned}
$$

$$\frac{d[R_2V_{164}N_1]_{B,i}}{dt} = -k^B_{int,V164R2N1}[R_2V_{164}N_1]_{B,i} + k^B_{c,V164R2,N1}[V_{164}R_2]_{B,i}[N_1]_{B,i} - k^B_{off,V164R2N1}[R_2V_{164}N_1]_{B,i} + k^B_{c,V164N1,R2}[V_{164}N_1]_{B,i}[R_2]_{B,i} - k^B_{off,V164N1R2}[R_2V_{164}N_1]_{B,i} \quad \text{(S.70, S.71)}$$

$$\frac{d[V_{120}R_1]_{B,i}}{dt} = -k^B_{int,V120R1}[V_{120}R_1]_{B,i} + k^B_{on,V120,R1}[V_{120}]_{B,i}[R_1]_{B,i} - k^B_{off,V120R1}[V_{120}R_1]_{B,i} - k^B_{c,R1,N1}[V_{120}R_1]_{B,i}[N_1]_{B,i} + k^B_{dissoc,R1N1}[V_{120}R_1N_1]_{B,i} \quad \text{(S.72, S.73)}$$

$$\frac{d[V_{120}R_2]_{B,i}}{dt} = -k^B_{int,V120R2}[V_{120}R_2]_{B,i} + k^B_{on,V120,R2}[V_{120}]_B[R_2]_{B,i} - k^B_{off,V120R2}[V_{120}R_2]_{B,i} \quad \text{(S.74, S.75)}$$

$$\frac{d[V_{120}R_1N_1]_{B,i}}{dt} = -k^B_{intV120R1N1}[V_{120}R_1N_1]_{B,i} + k^B_{c,V120R1,N1}[V_{120}R_1]_{B,i}[N_1]_{B,i} - k^B_{dissoc,V120N1}[V_{120}R_1N_1]_{B,i} + k^B_{on,V120R1N1}[V_{120}]_B[R_1N_1]_{B,i} - k^B_{off,V120R1N1}[V_{120}R_1N_1]_{B,i} \quad \text{(S.76, S.77)}$$

$$\frac{d[V_{165}R_1]_{B,i}}{dt} = -k^B_{int,V165R1}[V_{165}R_1]_{B,i} + k^B_{on,V165,R1}[V_{165}]_B[R_1]_{B,i} - k^B_{off,V165R1}[V_{165}R_1]_{B,i} \quad \text{(S.78, S.79)}$$

$$\frac{d[V_{165}R_2]_{B,i}}{dt} = -k^B_{int,V165R2}[V_{165}R_2]_{B,i} + k^B_{on,V165,R2}[V_{165}]_B[R_2]_{B,i} - k^B_{off,V165R2}[V_{165}R_2]_{B,i} \quad \text{(S.80, S.81)}$$

$$\frac{d[V_{165}N_1]_{B,i}}{dt} = -k^B_{int,V165N1}[V_{165}N_1]_{B,i} + k^B_{on,V165,N1}[V_{165}]_B[N_1]_{B,i} - k^B_{off,V165N1}[V_{165}N_1]_{B,i} - k^B_{c,V165N1,R2}[V_{165}N_1]_{B,i}[R_2]_{B,i} + k^B_{off,V165N1R2}[R_2V_{165}N_1]_{B,i} \quad \text{(S.82, S.83)}$$

$$\frac{d[R_2V_{165}N_1]_{B,i}}{dt} = -k^B_{int,V165R2N1}[R_2V_{165}N_1]_{B,i} + k^B_{c,V165R2,N1}[V_{165}R_2]_{B,i}[N_1]_{B,i} - k^B_{off,V165R2N1}[R_2V_{165}N_1]_{B,i} + k^B_{c,V165N1,R2}[V_{165}N_1]_{B,i}[R_2]_{B,i} - k^B_{off,V165N1R2}[R_2V_{165}N_1]_{B,i} \quad \text{(S.84, S.85)}$$

$$\frac{d[V_{121}R_1]_{B,i}}{dt} = -k^B_{int,V121R1}[V_{121}R_1]_{B,i} + k^B_{on,V121,R1}[V_{121}]_{B,i}[R_1]_{B,i} - k^B_{off,V121R1}[V_{121}R_1]_{B,i} - k^B_{c,R1,N1}[V_{121}R_1]_{B,i}[N_1]_{B,i} + k^B_{dissoc,R1N1}[V_{121}R_1N_1]_{B,i} \quad \text{(S.86, S.87)}$$

$$\frac{d[V_{121}R_2]_{B,i}}{dt} = -k^B_{int,V121R2}[V_{121}R_2]_{B,i} + k^B_{on,V121,R2}[V_{121}]_B[R_2]_{B,i} - k^B_{off,V121R2}[V_{121}R_2]_{B,i} \qquad \text{(S.88, S.89)}$$

$$\begin{aligned}\frac{d[V_{121}R_1N_1]_{B,i}}{dt} = &-k^B_{intV121R1N1}[V_{121}R_1N_1]_{B,i} \\ &+k^B_{c,V121R1,N1}[V_{121}R_1]_{B,i}[N_1]_{B,i} - k^B_{dissoc,V121N1}[V_{121}R_1N_1]_{B,i} \\ &+k^B_{on,V121R1N1}[V_{121}]_B[R_1N_1]_{B,i} - k^B_{off,V121R1N1}[V_{121}R_1N_1]_{B,i}\end{aligned} \qquad \text{(S.90, S.91)}$$

$$\begin{aligned}\frac{d[R_1N_1]_{B,i}}{dt} = &-k^B_{int,R1N1}[R_1N_1]_{B,i} \\ &+k^B_{c,R1,N1}[R_1]_{B,i}[N_1]_{B,i} - k^B_{dissoc,R1N1}[R_1N_1]_{B,i} \\ &-k^B_{on,V120,R1}[V_{120}]_B[R_1N_1]_{B,i} + k^B_{off,V120R1}[V_{120}R_1N_1]_{B,i} \\ &-k^B_{on,V121,R1}[V_{121}]_B[R_1N_1]_{B,i} + k^B_{off,V121R1}[V_{121}R_1N_1]_{B,i}\end{aligned} \qquad \text{(S.92, S.93)}$$

$$\begin{aligned}\frac{d[A]_B}{dt} = &\, q_A - c_A[A]_B - k^B_{on,V164,A}[V_{164}]_B[A]_B + k^B_{off,V164A}[V_{164}A]_B \\ &-k^B_{on,V120,A}[V_{120}]_B[A]_B + k^B_{off,V120A}[V_{120}A]_B \\ &-k^B_{on,V165,A}[V_{165}]_B[A]_B + k^B_{off,V165A}[V_{165}A]_B \\ &-k^B_{on,V121,A}[V_{121}]_B[A]_B + k^B_{off,V121A}[V_{121}A]_B \\ &-k^{BN}_{p,A}\frac{S_{NB}}{U_p}[A]_B + \left(\frac{k_L + k^{NB}_{p,A}S_{NB}}{U_B}\right)\frac{[A]_N}{K_{AV,N}} \\ &-k^{BT}_{p,A}\frac{S_{TB}}{U_p}[A]_B + \left(\frac{k^{TB}_{p,A}S_{TB}}{U_B}\right)\frac{[A]_T}{K_{AV,T}}\end{aligned} \qquad \text{(S.94)}$$

$$\begin{aligned}\frac{d[V_{164}A]_B}{dt} = &-c_{V164A}[V_{164}A]_B + k^B_{on,V164,A}[V_{164}]_B[A]_B - k^B_{off,V164A}[V_{164}A]_B \\ &-k^{BN}_{p,A}\frac{S_{NB}}{U_p}[V_{164}A]_B + \left(\frac{k_L + k^{NB}_{p,A}S_{NB}}{U_B}\right)\frac{[V_{164}A]_N}{K_{AV,N}} \\ &-k^{BT}_{p,A}\frac{S_{TB}}{U_p}[V_{164}A]_B + \left(\frac{k^{TB}_{p,A}S_{TB}}{U_B}\right)\frac{[V_{164}A]_T}{K_{AV,T}}\end{aligned} \qquad \text{(S.95)}$$

$$\frac{d[V_{120}A]_B}{dt} = -c_{V120A}[V_{120}A]_B + k^B_{on,V120,A}[V_{120}]_B[A]_B - k^B_{off,V120A}[V_{120}A]_B$$
$$-k^{BN}_{p,A}\frac{S_{NB}}{U_p}[V_{120}A]_B + \left(\frac{k_L + k^{NB}_{p,A}S_{NB}}{U_B}\right)\frac{[V_{120}A]_N}{K_{AV,N}}$$
$$-k^{BT}_{p,A}\frac{S_{TB}}{U_p}[V_{120}A]_B + \left(\frac{k^{TB}_{p,A}S_{TB}}{U_B}\right)\frac{[V_{120}A]_T}{K_{AV,T}} \quad \text{(S.96)}$$

$$\frac{d[V_{165}A]_B}{dt} = -c_{V165A}[V_{165}A]_B + k^B_{on,V165,A}[V_{165}]_B[A]_B - k^B_{off,V165A}[V_{165}A]_B$$
$$-k^{BN}_{p,A}\frac{S_{NB}}{U_p}[V_{165}A]_B + \left(\frac{k_L + k^{NB}_{p,A}S_{NB}}{U_B}\right)\frac{[V_{165}A]_N}{K_{AV,N}} \quad \text{(S.97)}$$
$$-k^{BT}_{p,A}\frac{S_{TB}}{U_p}[V_{165}A]_B + \left(\frac{k^{TB}_{p,A}S_{TB}}{U_B}\right)\frac{[V_{165}A]_T}{K_{AV,T}}$$

$$\frac{d[V_{121}A]_B}{dt} = -c_{V121A}[V_{121}A]_B + k^B_{on,V121,A}[V_{121}]_B[A]_B - k^B_{off,V121A}[V_{121}A]_B$$
$$-k^{BN}_{p,A}\frac{S_{NB}}{U_p}[V_{121}A]_B + \left(\frac{k_L + k^{NB}_{p,A}S_{NB}}{U_B}\right)\frac{[V_{121}A]_N}{K_{AV,N}} \quad \text{(S.98)}$$
$$-k^{BT}_{p,A}\frac{S_{TB}}{U_p}[V_{121}A]_B + \left(\frac{k^{TB}_{p,A}S_{TB}}{U_B}\right)\frac{[V_{121}A]_T}{K_{AV,T}}$$

$$\frac{d[sR_1]_B}{dt} = q^B_{sR1} - \left(c_{sR1} + k_{\deg,sR1}\right)[sR_1]_B - k^B_{on,V164,sR1}[V_{164}]_B[sR_1]_B + k^B_{off,V164sR1}[V_{164}sR_1]_B$$
$$-k^B_{on,V165,sR1}[V_{165}]_B[sR_1]_B + k^B_{off,V165sR1}[V_{165}sR_1]_B$$
$$-k^B_{on,V120,sR1}[V_{120}]_B[sR_1]_B + k^B_{off,V120sR1}[V_{120}sR_1]_B$$
$$-k^B_{on,V121,sR1}[V_{121}]_B[sR_1]_B + k^B_{off,V121sR1}[V_{121}sR_1]_B$$
$$-k^B_{on,sR1,N1}[sR_1]_B[N_1]_{B,i} + k^B_{off,sR1N1}[sR_1N_1]_{B,i}$$
$$-k^{BN}_{p,sR1}\frac{S_{NB}}{U_p}[sR_1]_B + \left(\frac{k_L + k^{NB}_{p,sR1}S_{NB}}{U_B}\right)\frac{[sR_1]_N}{K_{AV,N}}$$
$$-k^{BT}_{p,sR1}\frac{S_{TB}}{U_p}[sR_1]_B + \left(\frac{k^{TB}_{p}S_{TB}}{U_B}\right)\frac{[sR_1]_T}{K_{AV,T}} \quad \text{(S.99)}$$

$$\frac{d[sR_1N_1]_{B,i}}{dt} = -k^B_{int,sR1N1}[sR_1N_1]_{B,i} + k^B_{on,sR1,N1}[sR_1]_B[N_1]_{B,i} - k^B_{off,sR1N1}[sR_1N_1]_{B,i}$$
$$-k^B_{on,V120,sR1N1}[V_{120}]_B[sR_1N_1]_{B,i} + k^B_{off,V120,sR1N1}[V_{120}sR_1N_1]_{B,i} \qquad \text{(S.100, S.101)}$$
$$-k^B_{on,V121,sR1N1}[V_{121}]_B[sR_1N_1]_{B,i} + k^B_{off,V121,sR1N1}[V_{121}sR_1N_1]_{B,i}$$

$$\frac{d[V_{164}sR_1]_B}{dt} = -\left(c_{sR1} + k_{\deg,VsR1}\right)[V_{164}sR_1]_B$$
$$+k^B_{on,V164,sR1}[V_{164}]_N[sR_1]_B - k^B_{off,V164sR1}[V_{164}sR_1]_B$$
$$-k^{BN}_{p,sR1}\frac{S_{NB}}{U_p}[V_{164}sR_1]_B + \left(\frac{k_L + k^{NB}_{p,sR1}S_{NB}}{U_B}\right)\frac{[V_{164}sR_1]_N}{K_{AV,N}} \qquad \text{(S.102)}$$
$$-k^{BT}_{p,sR1}\frac{S_{TB}}{U_p}[V_{164}sR_1]_B + \left(\frac{k^{TB}_p S_{TB}}{U_B}\right)\frac{[V_{164}sR_1]_T}{K_{AV,T}}$$

$$\frac{d[V_{120}sR_1]_B}{dt} = -\left(c_{sR1} + k_{\deg,VsR1}\right)[V_{120}sR_1]_B$$
$$+k^B_{on,V120,sR1}[V_{120}]_B[sR_1]_B - k^B_{off,V120sR1}[V_{120}sR_1]_B$$
$$-k^B_{on,sR1,N1}[V_{120}sR_1]_B[N_1]_{B,i} + k^B_{off,R1sN1}[V_{120}sR_1N_1]_{B,i} \qquad \text{(S.103)}$$
$$-k^{BN}_{p,sR1}\frac{S_{NB}}{U_p}[V_{120}sR_1]_B + \left(\frac{k_L + k^{NB}_{p,sR1}S_{NB}}{U_B}\right)\frac{[V_{120}sR_1]_N}{K_{AV,N}}$$
$$-k^{BT}_{p,sR1}\frac{S_{TB}}{U_p}[V_{120}sR_1]_B + \left(\frac{k^{TB}_p S_{TB}}{U_B}\right)\frac{[V_{120}sR_1]_T}{K_{AV,T}}$$

$$\frac{d[V_{120}sR_1N_1]_{B,i}}{dt} = -k^B_{intV120sR1N1}[V_{120}sR_1N_1]_{B,i}$$
$$+k^B_{on,sR1,N1}[V_{120}R_1]_B[N_1]_{B,i} - k^N_{off,sR1N1}[V_{120}sR_1N_1]_{B,i} \qquad \text{(S.104, S.105)}$$
$$+k^B_{on,V120sR1N1}[V_{120}]_B[R_1N_1]_{B,i} - k^N_{off,V120sR1N1}[V_{120}sR_1N_1]_{B,i}$$

$$\frac{d[V_{165}sR_1]_B}{dt} = -\left(c_{sR1} + k_{\deg,VsR1}\right)[V_{165}sR_1]_B + k^B_{on,V165,sR1}[V_{165}]_B[sR_1]_B - k^B_{off,V165sR1}[V_{165}sR_1]_B$$
$$-k^{BN}_{p,sR1}\frac{S_{NB}}{U_p}[V_{165}sR_1]_B + \left(\frac{k_L + k^{NB}_{p,sR1}S_{NB}}{U_B}\right)\frac{[V_{165}sR_1]_N}{K_{AV,N}} \qquad \text{(S.106)}$$
$$-k^{BT}_{p,sR1}\frac{S_{TB}}{U_p}[V_{165}sR_1]_B + \left(\frac{k^{TB}_p S_{TB}}{U_B}\right)\frac{[V_{165}sR_1]_T}{K_{AV,T}}$$

$$\frac{d[V_{121}sR_1]_B}{dt} = -\left(c_{sR1} + k_{\deg,VsR1}\right)[V_{121}sR_1]_B$$
$$+k^B_{on,V121,sR1}[V_{121}]_B[sR_1]_B - k^B_{off,V121sR1}[V_{121}sR_1]_B$$
$$-k^B_{on,sR1,N1}[V_{121}sR_1]_B[N_1]_{B,i} + k^B_{off,R1sN1}[V_{121}sR_1N_1]_{B,i} \qquad \text{(S.107)}$$
$$-k^{BN}_{p,sR1}\frac{S_{NB}}{U_p}[V_{121}sR_1]_B + \left(\frac{k_L + k^{NB}_{p,sR1}S_{NB}}{U_B}\right)\frac{[V_{121}sR_1]_N}{K_{AV,N}}$$
$$-k^{BT}_{p,sR1}\frac{S_{TB}}{U_p}[V_{121}sR_1]_B + \left(\frac{k^{TB}_p S_{TB}}{U_B}\right)\frac{[V_{121}sR_1]_T}{K_{AV,T}}$$

$$\frac{d[V_{121}sR_1N_1]_{B,i}}{dt} = -k^B_{intV121sR1N1}[V_{121}sR_1N_1]_{B,i}$$
$$+k^B_{on,sR1,N1}[V_{121}R_1]_B[N_1]_{B,i} - k^B_{off,sR1N1}[V_{121}sR_1N_1]_{B,i} \qquad \text{(S.108, S.109)}$$
$$+k^B_{on,V121sR1N1}[V_{121}]_B[R_1N_1]_{B,i} - k^B_{off,V121sR1N1}[V_{121}sR_1N_1]_{B,i}$$

$$\frac{d[\alpha 2\mathrm{M}]_B}{dt} = k_{syn,\alpha 2\mathrm{M}} - c_{\alpha 2\mathrm{M}}[\alpha 2\mathrm{M}]_B$$
$$-k^B_{on,\alpha 2\mathrm{M},V164}[\alpha 2\mathrm{M}]_B[V_{164}]_B + k^B_{off,\alpha 2\mathrm{M}V164}[\alpha 2\mathrm{M}\cdot V_{164}]_B$$
$$-k^B_{on,\alpha 2\mathrm{M},V120}[\alpha 2\mathrm{M}]_B[V_{120}]_B + k^B_{off,\alpha 2\mathrm{M}V120}[\alpha 2\mathrm{M}\cdot V_{120}]_B \qquad \text{(S.110)}$$
$$-k^B_{on,\alpha 2\mathrm{M},V165}[\alpha 2\mathrm{M}]_B[V_{165}]_B + k^B_{off,\alpha 2\mathrm{M}V165}[\alpha 2\mathrm{M}\cdot V_{165}]_B$$
$$-k^B_{on,\alpha 2\mathrm{M},V121}[\alpha 2\mathrm{M}]_B[V_{121}]_B + k^B_{off,\alpha 2\mathrm{M}V121}[\alpha 2\mathrm{M}\cdot V_{121}]_B$$

$$\frac{d[\alpha 2\mathrm{M}\cdot V_{164}]_B}{dt} = -c_{\alpha 2\mathrm{M}V164}[\alpha 2\mathrm{M}\cdot V_{164}]_B$$
$$-k^B_{on,V164,R1}[\alpha 2\mathrm{M}\cdot V_{164}]_B[R_1]_{B,i} + k^B_{off,V164R1}[\alpha 2\mathrm{M}\cdot V_{164}R_1]_{B,i}$$
$$-k^B_{on,V164,R2}[\alpha 2\mathrm{M}\cdot V_{164}]_B[R_2]_{B,i} + k^B_{off,V164R2}[\alpha 2\mathrm{M}\cdot V_{164}R_2]_{B,i} \qquad \text{(S.111)}$$
$$-k^B_{on,V164,N1}[\alpha 2\mathrm{M}\cdot V_{164}]_B[N_1]_{B,i} + k^B_{off,V164N1}[\alpha 2\mathrm{M}\cdot V_{164}N_1]_{B,i}$$
$$-k^B_{on,V164,A}[\alpha 2\mathrm{M}\cdot V_{164}]_B[A]_B + k^B_{off,V164A}[\alpha 2\mathrm{M}\cdot V_{164}A]_B$$

$$\frac{d[\alpha 2\mathrm{M}\cdot V_{120}]_B}{dt} = -c_{\alpha 2\mathrm{M}V120}[\alpha 2\mathrm{M}\cdot V_{120}]_B$$
$$-k^B_{on,V120,R1}[\alpha 2\mathrm{M}\cdot V_{120}]_B[R_1]_{B,i} + k^B_{off,V120R1}[\alpha 2\mathrm{M}\cdot V_{120}R_1]_{B,i}$$
$$-k^B_{on,V120,R1N1}[\alpha 2\mathrm{M}\cdot V_{120}]_B[R_1N_1]_{B,i} + k^B_{off,V120R1N1}[\alpha 2\mathrm{M}\cdot V_{120}R_1N_1]_{B,i} \quad \text{(S.112)}$$
$$-k^B_{on,V120,R2}[\alpha 2\mathrm{M}\cdot V_{120}]_B[R_2]_{B,i} + k^B_{off,V120R2}[\alpha 2\mathrm{M}\cdot V_{120}R_2]_{B,i}$$
$$-k^B_{on,V120,A}[\alpha 2\mathrm{M}\cdot V_{120}]_B[A]_B + k^B_{off,V120A}[\alpha 2\mathrm{M}\cdot V_{120}A]_B$$

$$\frac{d[\alpha 2\mathrm{M}\cdot V_{164}A]_B}{dt} = -c_{\alpha 2\mathrm{M}V164A}[\alpha 2\mathrm{M}\cdot V_{164}A]_B$$
$$+k^B_{on,\alpha 2\mathrm{M}V164,A}[\alpha 2\mathrm{M}\cdot V_{164}]_B[A]_B - k^B_{off,\alpha 2\mathrm{M}V164A}[\alpha 2\mathrm{M}\cdot V_{164}A]_B$$
$$\text{(S.113)}$$

$$\frac{d[\alpha 2\mathrm{M}\cdot V_{120}A]_B}{dt} = -c_{\alpha 2\mathrm{M}V120A}[\alpha 2\mathrm{M}\cdot V_{120}A]_B$$
$$+k^B_{on,\alpha 2\mathrm{M}V120,A}[\alpha 2\mathrm{M}\cdot V_{120}]_B[A]_B - k^B_{off,\alpha 2\mathrm{M}V120A}[\alpha 2\mathrm{M}\cdot V_{120}A]_B$$
$$\text{(S.114)}$$

$$\frac{d[\alpha 2\mathrm{M}\cdot V_{164}R_1]_{B,i}}{dt} = k^B_{on,V164,R1}[\alpha 2\mathrm{M}\cdot V_{164}]_B[R_1]_{B,i} - k^B_{off,V164R1}[\alpha 2\mathrm{M}\cdot V_{164}R_1]_{B,i} \quad \text{(S.115, S.116)}$$

$$\frac{d[\alpha 2\mathrm{M}\cdot V_{164}R_2]_{B,i}}{dt} = k^B_{on,V164,R2}[\alpha 2\mathrm{M}\cdot V_{164}]_B[R_2]_{B,i} - k^B_{off,V164R2}[\alpha 2\mathrm{M}\cdot V_{164}R_2]_{B,i} \quad \text{(S.117, S.118)}$$

$$\frac{d[\alpha 2\mathrm{M}\cdot V_{164}N_1]_{B,i}}{dt} = k^B_{on,V164,N1}[\alpha 2\mathrm{M}\cdot V_{164}]_B[N_1]_{B,i} - k^B_{off,V164N1}[\alpha 2\mathrm{M}\cdot V_{164}N_1]_{B,i} \quad \text{(S.119, S.120)}$$
$$-k^B_{c,V164N1,R2}[\alpha 2\mathrm{M}\cdot V_{164}N_1]_{B,i}[R_2]_{B,i} + k^B_{off,V164N1R2}[\alpha 2\mathrm{M}\cdot R_2V_{164}N_1]_{B,i}$$

$$\frac{d[\alpha 2\mathrm{M}\cdot R_2V_{164}N_1]_{B,i}}{dt} = k^B_{c,V164R2,N1}[\alpha 2\mathrm{M}\cdot V_{164}R_2]_{B,i}[N_1]_{B,i} - k^B_{off,V164R2N1}[\alpha 2\mathrm{M}\cdot R_2V_{164}N_1]_{B,i} \quad \text{(S.121, S.122)}$$
$$+k^B_{c,V164N1,R2}[\alpha 2\mathrm{M}\cdot V_{164}N_1]_{B,i}[R_2]_{B,i} - k^B_{off,V164N1R2}[\alpha 2\mathrm{M}\cdot R_2V_{164}N_1]_{B,i}$$

$$\frac{d[\alpha 2\mathrm{M}\cdot V_{120}R_1]_{B,i}}{dt} = k^B_{on,V120,R1}[\alpha 2\mathrm{M}\cdot V_{120}]_{B,i}[R_1]_{B,i} - k^B_{off,V120R1}[\alpha 2\mathrm{M}\cdot V_{120}R_1]_{B,i} \quad \text{(S.123, S.124)}$$
$$-k^B_{c,R1,N1}[\alpha 2\mathrm{M}\cdot V_{120}R_1]_{B,i}[N_1]_{B,i} + k^B_{dissoc,R1N1}[\alpha 2\mathrm{M}\cdot V_{120}R_1N_1]_{B,i}$$

$$\frac{d[\alpha 2\mathrm{M}\cdot V_{120}R_2]_{B,i}}{dt} = k^B_{on,V120,R2}[\alpha 2\mathrm{M}\cdot V_{120}]_B[R_2]_{B,i} - k^B_{off,V120R2}[\alpha 2\mathrm{M}\cdot V_{120}R_2]_{B,i} \quad \text{(S.125, S.126)}$$

$$\frac{d[\alpha 2\mathrm{M}\cdot V_{120}R_1N_1]_{B,i}}{dt} = k^B_{c,V120R1,N1}[\alpha 2\mathrm{M}\cdot V_{120}R_1]_{B,i}[N_1]_{B,i} - k^B_{dissoc,V120N1}[\alpha 2\mathrm{M}\cdot V_{120}R_1N_1]_{B,i} + k^B_{on,V120R1N1}[\alpha 2\mathrm{M}\cdot V_{120}]_B[R_1N_1]_{B,i} - k^B_{off,V120R1N1}[\alpha 2\mathrm{M}\cdot V_{120}R_1N_1]_{B,i} \quad \text{(S.127, S.128)}$$

$$\begin{aligned}\frac{d[\alpha 2\mathrm{M}_{fast}]_B}{dt} = {} & k_{syn,\alpha 2\mathrm{M}fast} - c_{\alpha 2\mathrm{M}fast}[\alpha 2\mathrm{M}_{fast}]_B \\ & - k^B_{on,\alpha 2\mathrm{M},V164}[\alpha 2\mathrm{M}_{fast}]_B[V_{164}]_B + k^B_{off,\alpha 2\mathrm{M}V164}[\alpha 2\mathrm{M}_{fast}\cdot V_{164}]_B \\ & - k^B_{on,\alpha 2\mathrm{M},V120}[\alpha 2\mathrm{M}_{fast}]_B[V_{120}]_B + k^B_{off,\alpha 2\mathrm{M}V120}[\alpha 2\mathrm{M}_{fast}\cdot V_{120}]_B \\ & - k^B_{on,\alpha 2\mathrm{M},V165}[\alpha 2\mathrm{M}_{fast}]_B[V_{165}]_B + k^B_{off,\alpha 2\mathrm{M}V165}[\alpha 2\mathrm{M}_{fast}\cdot V_{165}]_B \\ & - k^B_{on,\alpha 2\mathrm{M},V121}[\alpha 2\mathrm{M}_{fast}]_B[V_{121}]_B + k^B_{off,\alpha 2\mathrm{M}V121}[\alpha 2\mathrm{M}_{fast}\cdot V_{121}]_B\end{aligned} \quad \text{(S.129)}$$

$$\begin{aligned}\frac{d[\alpha 2\mathrm{M}_{fast}\cdot V_{164}]_B}{dt} = {} & -c_{\alpha 2\mathrm{M}fastV164}[\alpha 2\mathrm{M}_{fast}\cdot V_{164}]_B \\ & - k^B_{on,V164,R1}[\alpha 2\mathrm{M}_{fast}\cdot V_{164}]_B[R_1]_{B,i} + k^B_{off,V164R1}[\alpha 2\mathrm{M}_{fast}\cdot V_{164}R_1]_{B,i} \\ & - k^B_{on,V164,R2}[\alpha 2\mathrm{M}_{fast}\cdot V_{164}]_B[R_2]_{B,i} + k^B_{off,V164R2}[\alpha 2\mathrm{M}_{fast}\cdot V_{164}R_2]_{B,i} \\ & - k^B_{on,V164,N1}[\alpha 2\mathrm{M}_{fast}\cdot V_{164}]_B[N_1]_{B,i} + k^B_{off,V164N1}[\alpha 2\mathrm{M}_{fast}\cdot V_{164}N_1]_{B,i}\end{aligned} \quad \text{(S.130)}$$

$$\begin{aligned}\frac{d[\alpha 2\mathrm{M}_{fast}\cdot V_{120}]_B}{dt} = {} & -c_{\alpha 2\mathrm{M}fastV120}[\alpha 2\mathrm{M}_{fast}\cdot V_{120}]_B \\ & - k^B_{on,V120,R1}[\alpha 2\mathrm{M}_{fast}\cdot V_{120}]_B[R_1]_{B,i} + k^B_{off,V120R1}[\alpha 2\mathrm{M}_{fast}\cdot V_{120}R_1]_{B,i} \\ & - k^B_{on,V120,R1N1}[\alpha 2\mathrm{M}_{fast}\cdot V_{120}]_B[R_1N_1]_{B,i} + k^B_{off,V120R1N1}[\alpha 2\mathrm{M}_{fast}\cdot V_{120}R_1N_1]_{B,i} \\ & - k^B_{on,V120,R2}[\alpha 2\mathrm{M}_{fast}\cdot V_{120}]_B[R_2]_{B,i} + k^B_{off,V120R2}[\alpha 2\mathrm{M}_{fast}\cdot V_{120}R_2]_{B,i} \\ & - k^B_{on,V120,A}[\alpha 2\mathrm{M}_{fast}\cdot V_{120}]_B[A]_B + k^B_{off,V120A}[\alpha 2\mathrm{M}_{fast}\cdot V_{120}A]_B\end{aligned} \quad \text{(S.131)}$$

$$\frac{d[\alpha 2\mathrm{M}_{fast}\cdot V_{164}R_1]_{B,i}}{dt} = k^B_{on,V164,R1}[\alpha 2\mathrm{M}_{fast}\cdot V_{164}]_B[R_1]_{B,i} - k^B_{off,V164R1}[\alpha 2\mathrm{M}_{fast}\cdot V_{164}R_1]_{B,i} \quad \text{(S.132, S.133)}$$

$$\frac{d[\alpha 2\mathrm{M}_{fast}\cdot V_{164}R_2]_{B,i}}{dt} = k^B_{on,V164,R2}[\alpha 2\mathrm{M}_{fast}\cdot V_{164}]_B[R_2]_{B,i} - k^B_{off,V164R2}[\alpha 2\mathrm{M}_{fast}\cdot V_{164}R_2]_{B,i} \quad \text{(S.134, S.135)}$$

$$\frac{d[\alpha 2\mathrm{M}_{fast}\cdot V_{164}N_1]_{B,i}}{dt} = k^B_{on,V164,N1}[\alpha 2\mathrm{M}_{fast}\cdot V_{164}]_B[N_1]_{B,i} - k^B_{off,V164N1}[\alpha 2\mathrm{M}_{fast}\cdot V_{164}N_1]_{B,i} - k^B_{c,V164N1,R2}[\alpha 2\mathrm{M}_{fast}\cdot V_{164}N_1]_{B,i}[R_2]_{B,i} + k^B_{off,V164N1R2}[\alpha 2\mathrm{M}_{fast}\cdot R_2V_{164}N_1]_{B,i} \quad \text{(S.136, S.137)}$$

$$\frac{d[\alpha 2\mathrm{M}_{fast}\cdot R_2V_{164}N_1]_{B,i}}{dt}=k^B_{c,V164R2,N1}[\alpha 2\mathrm{M}_{fast}\cdot V_{164}R_2]_{B,i}[N_1]_{B,i}-k^B_{off,V164R2N1}[\alpha 2\mathrm{M}_{fast}\cdot R_2V_{164}N_1]_{B,i} +k^B_{c,V164N1,R2}[\alpha 2\mathrm{M}_{fast}\cdot V_{164}N_1]_{B,i}[R_2]_{B,i}-k^B_{off,V164N1R2}[\alpha 2\mathrm{M}_{fast}\cdot R_2V_{164}N_1]_{B,i} \quad \text{(S.138, S.139)}$$

$$\frac{d[\alpha 2\mathrm{M}_{fast}\cdot V_{120}R_1]_{B,i}}{dt}=k^B_{on,V120,R1}[\alpha 2\mathrm{M}_{fast}\cdot V_{120}]_{B,i}[R_1]_{B,i}-k^B_{off,V120R1}[\alpha 2\mathrm{M}_{fast}\cdot V_{120}R_1]_{B,i} -k^B_{c,R1,N1}[\alpha 2\mathrm{M}_{fast}\cdot V_{120}R_1]_{B,i}[N_1]_{B,i}+k^B_{dissoc,R1N1}[\alpha 2\mathrm{M}_{fast}\cdot V_{120}R_1N_1]_{B,i} \quad \text{(S.140, S.141)}$$

$$\frac{d[\alpha 2\mathrm{M}_{fast}\cdot V_{120}R_2]_{B,i}}{dt}=k^B_{on,V120,R2}[\alpha 2\mathrm{M}_{fast}\cdot V_{120}]_B[R_2]_{B,i}-k^B_{off,V120R2}[\alpha 2\mathrm{M}_{fast}\cdot V_{120}R_2]_{B,i} \quad \text{(S.142, S.143)}$$

$$\frac{d[\alpha 2\mathrm{M}_{fast}\cdot V_{120}R_1N_1]_{B,i}}{dt}=k^B_{c,V120R1,N1}[\alpha 2\mathrm{M}_{fast}\cdot V_{120}R_1]_{B,i}[N_1]_{B,i}-k^B_{dissoc,V120N1}[\alpha 2\mathrm{M}_{fast}\cdot V_{120}R_1N_1]_{B,i} +k^B_{on,V120R1N1}[\alpha 2\mathrm{M}_{fast}\cdot V_{120}]_B[R_1N_1]_{B,i}-k^B_{off,V120R1N1}[\alpha 2\mathrm{M}_{fast}\cdot V_{120}R_1N_1]_{B,i} \quad \text{(S.144, S.145)}$$

$$\begin{aligned}\frac{d[\alpha 2\mathrm{M}\cdot V_{165}]_B}{dt}=&-c_{\alpha 2\mathrm{M}V165}[\alpha 2\mathrm{M}\cdot V_{165}]_B\\ &-k^B_{on,V165,R1}[\alpha 2\mathrm{M}\cdot V_{165}]_B[R_1]_{B,i}+k^B_{off,V165R1}[\alpha 2\mathrm{M}\cdot V_{165}R_1]_{B,i}\\ &-k^B_{on,V165,R2}[\alpha 2\mathrm{M}\cdot V_{165}]_B[R_2]_{B,i}+k^B_{off,V165R2}[\alpha 2\mathrm{M}\cdot V_{165}R_2]_{B,i}\\ &-k^B_{on,V165,N1}[\alpha 2\mathrm{M}\cdot V_{165}]_B[N_1]_{B,i}+k^B_{off,V165N1}[\alpha 2\mathrm{M}\cdot V_{165}N_1]_{B,i}\\ &-k^B_{on,V165,A}[\alpha 2\mathrm{M}\cdot V_{165}]_B[A]_B+k^B_{off,V165A}[\alpha 2\mathrm{M}\cdot V_{165}A]_B\end{aligned} \quad \text{(S.146)}$$

$$\begin{aligned}\frac{d[\alpha 2\mathrm{M}\cdot V_{121}]_B}{dt}=&-c_{\alpha 2\mathrm{M}V121}[\alpha 2\mathrm{M}\cdot V_{121}]_B\\ &-k^B_{on,V121,R1}[\alpha 2\mathrm{M}\cdot V_{121}]_B[R_1]_{B,i}+k^B_{off,V121R1}[\alpha 2\mathrm{M}\cdot V_{121}R_1]_{B,i}\\ &-k^B_{on,V121,R1N1}[\alpha 2\mathrm{M}\cdot V_{121}]_B[R_1N_1]_{B,i}+k^B_{off,V121R1N1}[\alpha 2\mathrm{M}\cdot V_{121}R_1N_1]_{B,i}\\ &-k^B_{on,V121,R2}[\alpha 2\mathrm{M}\cdot V_{121}]_B[R_2]_{B,i}+k^B_{off,V121R2}[\alpha 2\mathrm{M}\cdot V_{121}R_2]_{B,i}\\ &-k^B_{on,V121,A}[\alpha 2\mathrm{M}\cdot V_{121}]_B[A]_B+k^B_{off,V121A}[\alpha 2\mathrm{M}\cdot V_{121}A]_B\end{aligned} \quad \text{(S.147)}$$

$$\begin{aligned}\frac{d[\alpha 2\mathrm{M}\cdot V_{165}A]_B}{dt}=&-c_{\alpha 2\mathrm{M}V165A}[\alpha 2\mathrm{M}\cdot V_{165}A]_B\\ &+k^B_{on,\alpha 2\mathrm{M}V165,A}[\alpha 2\mathrm{M}\cdot V_{165}]_B[A]_B-k^B_{off,\alpha 2\mathrm{M}V165A}[\alpha 2\mathrm{M}\cdot V_{165}A]_B\end{aligned} \quad \text{(S.148)}$$

$$\begin{aligned}\frac{d[\alpha 2\mathrm{M}\cdot V_{121}A]_B}{dt}=&-c_{\alpha 2\mathrm{M}V121A}[\alpha 2\mathrm{M}\cdot V_{121}A]_B\\ &+k^B_{on,\alpha 2\mathrm{M}V121,A}[\alpha 2\mathrm{M}\cdot V_{121}]_B[A]_B-k^B_{off,\alpha 2\mathrm{M}V121A}[\alpha 2\mathrm{M}\cdot V_{121}A]_B\end{aligned} \quad \text{(S.149)}$$

$$\frac{d[\alpha 2\mathrm{M}\cdot V_{165}R_1]_{B,i}}{dt} = k^B_{on,V165,R1}[\alpha 2\mathrm{M}\cdot V_{165}]_B[R_1]_{B,i} - k^B_{off,V165R1}[\alpha 2\mathrm{M}\cdot V_{165}R_1]_{B,i} \quad \text{(S.150, S.151)}$$

$$\frac{d[\alpha 2\mathrm{M}\cdot V_{165}R_2]_{B,i}}{dt} = k^B_{on,V165,R2}[\alpha 2\mathrm{M}\cdot V_{165}]_B[R_2]_{B,i} - k^B_{off,V165R2}[\alpha 2\mathrm{M}\cdot V_{165}R_2]_{B,i} \quad \text{(S.152, S.153)}$$

$$\begin{aligned}\frac{d[\alpha 2\mathrm{M}\cdot V_{165}N_1]_{B,i}}{dt} &= k^B_{on,V165,N1}[\alpha 2\mathrm{M}\cdot V_{165}]_B[N_1]_{B,i} - k^B_{off,V165N1}[\alpha 2\mathrm{M}\cdot V_{165}N_1]_{B,i} \\ &\quad - k^B_{c,V165N1,R2}[\alpha 2\mathrm{M}\cdot V_{165}N_1]_{B,i}[R_2]_{B,i} + k^B_{off,V165N1R2}[\alpha 2\mathrm{M}\cdot R_2V_{165}N_1]_{B,i}\end{aligned} \quad \text{(S.154, S.155)}$$

$$\begin{aligned}\frac{d[\alpha 2\mathrm{M}\cdot R_2V_{165}N_1]_{B,i}}{dt} &= k^B_{c,V165R2,N1}[\alpha 2\mathrm{M}\cdot V_{165}R_2]_{B,i}[N_1]_{B,i} - k^B_{off,V165R2N1}[\alpha 2\mathrm{M}\cdot R_2V_{165}N_1]_{B,i} \\ &\quad + k^B_{c,V165N1,R2}[\alpha 2\mathrm{M}\cdot V_{165}N_1]_{B,i}[R_2]_{B,i} - k^B_{off,V165N1R2}[\alpha 2\mathrm{M}\cdot R_2V_{165}N_1]_{B,i}\end{aligned} \quad \text{(S.156, S.157)}$$

$$\begin{aligned}\frac{d[\alpha 2\mathrm{M}\cdot V_{121}R_1]_{B,i}}{dt} &= k^B_{on,V121,R1}[\alpha 2\mathrm{M}\cdot V_{121}]_{B,i}[R_1]_{B,i} - k^B_{off,V121R1}[\alpha 2\mathrm{M}\cdot V_{121}R_1]_{B,i} \\ &\quad - k^B_{c,R1,N1}[\alpha 2\mathrm{M}\cdot V_{121}R_1]_{B,i}[N_1]_{B,i} + k^B_{dissoc,R1N1}[\alpha 2\mathrm{M}\cdot V_{121}R_1N_1]_{B,i}\end{aligned} \quad \text{(S.158, S.159)}$$

$$\frac{d[\alpha 2\mathrm{M}\cdot V_{121}R_2]_{B,i}}{dt} = k^B_{on,V121,R2}[\alpha 2\mathrm{M}\cdot V_{121}]_B[R_2]_{B,i} - k^B_{off,V121R2}[\alpha 2\mathrm{M}\cdot V_{121}R_2]_{B,i} \quad \text{(S.160, S.161)}$$

$$\begin{aligned}\frac{d[\alpha 2\mathrm{M}\cdot V_{121}R_1N_1]_{B,i}}{dt} &= k^B_{c,V121R1,N1}[\alpha 2\mathrm{M}\cdot V_{121}R_1]_{B,i}[N_1]_{B,i} - k^B_{dissoc,V12N1}[\alpha 2\mathrm{M}\cdot V_{121}R_1N_1]_{B,i} \\ &\quad + k^B_{on,V121R1N1}[\alpha 2\mathrm{M}\cdot V_{121}]_B[R_1N_1]_{B,i} - k^B_{off,V121R1N1}[\alpha 2\mathrm{M}\cdot V_{121}R_1N_1]_{B,i}\end{aligned} \quad \text{(S.162, S.163)}$$

$$\begin{aligned}\frac{d[\alpha 2\mathrm{M}_{fast}\cdot V_{165}]_B}{dt} &= -c_{\alpha 2\mathrm{M}fastV165}[\alpha 2\mathrm{M}_{fast}\cdot V_{165}]_B \\ &\quad - k^B_{on,V165,R1}[\alpha 2\mathrm{M}_{fast}\cdot V_{165}]_B[R_1]_{B,i} + k^B_{off,V165R1}[\alpha 2\mathrm{M}_{fast}\cdot V_{165}R_1]_{B,i} \\ &\quad - k^B_{on,V165,R2}[\alpha 2\mathrm{M}_{fast}\cdot V_{165}]_B[R_2]_{B,i} + k^B_{off,V165R2}[\alpha 2\mathrm{M}_{fast}\cdot V_{165}R_2]_{B,i} \\ &\quad - k^B_{on,V165,N1}[\alpha 2\mathrm{M}_{fast}\cdot V_{165}]_B[N_1]_{B,i} + k^B_{off,V165N1}[\alpha 2\mathrm{M}_{fast}\cdot V_{165}N_1]_{B,i}\end{aligned} \quad \text{(S.164)}$$

$$\begin{aligned}\frac{d[\alpha 2\mathrm{M}_{fast}\cdot V_{121}]_B}{dt} &= -c_{\alpha 2\mathrm{M}fastV121}[\alpha 2\mathrm{M}_{fast}\cdot V_{121}]_B \\ &\quad - k^B_{on,V121,R1}[\alpha 2\mathrm{M}_{fast}\cdot V_{121}]_B[R_1]_{B,i} + k^B_{off,V121R1}[\alpha 2\mathrm{M}_{fast}\cdot V_{121}R_1]_{B,i} \\ &\quad - k^B_{on,V121,R1N1}[\alpha 2\mathrm{M}_{fast}\cdot V_{121}]_B[R_1N_1]_{B,i} + k^B_{off,V121R1N1}[\alpha 2\mathrm{M}_{fast}\cdot V_{121}R_1N_1]_{B,i} \\ &\quad - k^B_{on,V121,R2}[\alpha 2\mathrm{M}_{fast}\cdot V_{121}]_B[R_2]_{B,i} + k^B_{off,V121R2}[\alpha 2\mathrm{M}_{fast}\cdot V_{121}R_2]_{B,i} \\ &\quad - k^B_{on,V121,A}[\alpha 2\mathrm{M}_{fast}\cdot V_{121}]_B[A]_B + k^B_{off,V121A}[\alpha 2\mathrm{M}_{fast}\cdot V_{121}A]_B\end{aligned} \quad \text{(S.165)}$$

$$\frac{d[\alpha 2\mathrm{M}_{fast}\cdot V_{165}R_1]_{B,i}}{dt}=k^B_{on,V165,R1}[\alpha 2\mathrm{M}_{fast}\cdot V_{165}]_B[R_1]_{B,i}-k^B_{off,V165R1}[\alpha 2\mathrm{M}_{fast}\cdot V_{165}R_1]_{B,i} \quad \text{(S.166, S.167)}$$

$$\frac{d[\alpha 2\mathrm{M}_{fast}\cdot V_{164}R_2]_{B,i}}{dt}=k^B_{on,V164,R2}[\alpha 2\mathrm{M}_{fast}\cdot V_{164}]_B[R_2]_{B,i}-k^B_{off,V164R2}[\alpha 2\mathrm{M}_{fast}\cdot V_{164}R_2]_{B,i} \quad \text{(S.168, S.169)}$$

$$\begin{aligned}\frac{d[\alpha 2\mathrm{M}_{fast}\cdot V_{165}N_1]_{B,i}}{dt}&=k^B_{on,V165,N1}[\alpha 2\mathrm{M}_{fast}\cdot V_{165}]_B[N_1]_{B,i}-k^B_{off,V165N1}[\alpha 2\mathrm{M}_{fast}\cdot V_{165}N_1]_{B,i}\\&-k^B_{c,V165N1,R2}[\alpha 2\mathrm{M}_{fast}\cdot V_{165}N_1]_{B,i}[R_2]_{B,i}+k^B_{off,V165N1R2}[\alpha 2\mathrm{M}_{fast}\cdot R_2V_{165}N_1]_{B,i}\end{aligned} \quad \text{(S.170, S.171)}$$

$$\begin{aligned}\frac{d[\alpha 2\mathrm{M}_{fast}\cdot R_2V_{165}N_1]_{B,i}}{dt}&=k^B_{c,V165R2,N1}[\alpha 2\mathrm{M}_{fast}\cdot V_{165}R_2]_{B,i}[N_1]_{B,i}-k^B_{off,V165R2N1}[\alpha 2\mathrm{M}_{fast}\cdot R_2V_{165}N_1]_{B,i}\\&+k^B_{c,V165N1,R2}[\alpha 2\mathrm{M}_{fast}\cdot V_{165}N_1]_{B,i}[R_2]_{B,i}-k^B_{off,V165N1R2}[\alpha 2\mathrm{M}_{fast}\cdot R_2V_{165}N_1]_{B,i}\end{aligned} \quad \text{(S.172, S.173)}$$

$$\begin{aligned}\frac{d[\alpha 2\mathrm{M}_{fast}\cdot V_{121}R_1]_{B,i}}{dt}&=k^B_{on,V121,R1}[\alpha 2\mathrm{M}_{fast}\cdot V_{121}]_{B,i}[R_1]_{B,i}-k^B_{off,V121R1}[\alpha 2\mathrm{M}_{fast}\cdot V_{121}R_1]_{B,i}\\&-k^B_{c,R1,N1}[\alpha 2\mathrm{M}_{fast}\cdot V_{121}R_1]_{B,i}[N_1]_{B,i}+k^B_{dissoc,R1N1}[\alpha 2\mathrm{M}_{fast}\cdot V_{121}R_1N_1]_{B,i}\end{aligned} \quad \text{(S.174, S.175)}$$

$$\frac{d[\alpha 2\mathrm{M}_{fast}\cdot V_{121}R_2]_{B,i}}{dt}=k^B_{on,V121,R2}[\alpha 2\mathrm{M}_{fast}\cdot V_{121}]_B[R_2]_{B,i}-k^B_{off,V121R2}[\alpha 2\mathrm{M}_{fast}\cdot V_{121}R_2]_{B,i} \quad \text{(S.176, S.177)}$$

$$\begin{aligned}\frac{d[\alpha 2\mathrm{M}_{fast}\cdot V_{121}R_1N_1]_{B,i}}{dt}&=k^B_{c,V121R1,N1}[\alpha 2\mathrm{M}_{fast}\cdot V_{121}R_1]_{B,i}[N_1]_{B,i}-k^B_{dissoc,V121N1}[\alpha 2\mathrm{M}_{fast}\cdot V_{121}R_1N_1]_{B,i}\\&+k^B_{on,V121R1N1}[\alpha 2\mathrm{M}_{fast}\cdot V_{121}]_B[R_1N_1]_{B,i}-k^B_{off,V121R1N1}[\alpha 2\mathrm{M}_{fast}\cdot V_{121}R_1N_1]_{B,i}\end{aligned} \quad \text{(S.178, S.179)}$$

C. Interstitial space in tumor compartment

We denote the receptors and ligand-receptor complexes by the subscript *i* (*i*=T for tumor ECs; *i*=*tumor* for tumor cells).

$$\frac{d[V_{164}]_T}{dt} = -k_{\deg,V}[V_{164}]_T - k^T_{on,V164,MEBM}[V_{164}]_T[M_{EBM}]_T + k^T_{off,V164,MEBM}[V_{164}M_{EBM}]_T$$
$$-k^T_{on,V164,MPBM}[V_{164}]_T[M_{PBM}]_T + k^T_{off,V164,MPBM}[V_{164}M_{PBM}]_T$$
$$-k^T_{on,V164,MECM}[V_{164}]_T[M_{ECM}]_T + k^T_{off,V164,MECM}[V_{164}M_{ECM}]_T$$
$$-k^{T,i}_{on,V164,R1}[V_{164}]_T[R_1]_{T,i} + k^T_{off,V164R1}[V_{164}R_1]_{T,i}$$
$$-k^{T,i}_{on,V164,R2}[V_{164}]_T[R_2]_{T,i} + k^T_{off,V164R2}[V_{164}R_2]_{T,i} \qquad \text{(S.180)}$$
$$-k^{T,i}_{on,V164,N1}[V_{164}]_T[N_1]_{T,i} + k^T_{off,V164N1}[V_{164}N_1]_{T,i}$$
$$-k^{T,tumor}_{on,V164,N2}[V_{164}]_T[N_2]_{T,tumor} + k^{T,tumor}_{off,V164N2}[V_{164}N_2]_{T,tumor}$$
$$-k^T_{on,V164,A}[V_{164}]_T[A]_T + k^T_{off,V164A}[V_{164}A]_T$$
$$-\left(\frac{k_L + k^{TB}_{pV}S_{TB}}{U_T}\right)\frac{[V_{164}]_T}{K_{AV,T}} + k^{BT}_{pV}\frac{S_{TB}}{U_T}\frac{U_B}{U_P}[V_{164}]_B$$

$$\frac{d[V_{120}]_T}{dt} = -k_{\deg,V}[V_{120}]_T - k^T_{on,V120,R1}[V_{120}]_N[R_1]_{N,i} + k^T_{off,V120R1}[V_{120}R_1]_{T,i}$$
$$-k^T_{on,V120,R1N1}[V_{120}]_T[R_1N_1]_{T,i} + k^T_{off,V120R1N1}[V_{120}R_1N_1]_{T,i}$$
$$-k^T_{on,V120,R2}[V_{120}]_T[R_2]_{T,i} + k^T_{off,V120R2}[V_{120}R_2]_{T,i} \qquad \text{(S.181)}$$
$$-k^T_{on,V120,A}[V_{120}]_T[A]_T + k^T_{off,V120A}[V_{120}A]_T$$
$$-\left(\frac{k_L + k^{TB}_{pV}S_{TB}}{U_T}\right)\frac{[V_{120}]_T}{K_{AV,T}} + k^{BT}_{pV}\frac{S_{TB}}{U_T}\frac{U_B}{U_P}[V_{120}]_B$$

$$\frac{d[V_{165}]_T}{dt} = q^T_{V165} - k_{\deg,V}[V_{165}]_T - k^T_{on,V165,MEBM}[V_{165}]_T[M_{EBM}]_T + k^T_{off,V165,MEBM}[V_{165}M_{EBM}]_T$$
$$-k^T_{on,V165,MPBM}[V_{165}]_T[M_{PBM}]_T + k^T_{off,V165,MPBM}[V_{165}M_{PBM}]_T$$
$$-k^T_{on,V165,MECM}[V_{165}]_T[M_{ECM}]_T + k^T_{off,V165,MECM}[V_{165}M_{ECM}]_T$$
$$-k^{T,i}_{on,V165,R1}[V_{165}]_T[R_1]_{T,i} + k^T_{off,V165R1}[V_{165}R_1]_{T,i}$$
$$-k^{T,i}_{on,V165,R2}[V_{165}]_T[R_2]_{T,i} + k^T_{off,V165R2}[V_{165}R_2]_{T,i} \qquad \text{(S.182)}$$
$$-k^{T,i}_{on,V165,N1}[V_{165}]_T[N_1]_{T,i} + k^T_{off,V165N1}[V_{165}N_1]_{T,i}$$
$$-k^{T,tumor}_{on,V165,N2}[V_{165}]_T[N_2]_{T,tumor} + k^{T,tumor}_{off,V165N2}[V_{165}N_2]_{T,tumor}$$
$$-k^T_{on,V165,A}[V_{165}]_T[A]_T + k^T_{off,V165A}[V_{165}A]_T$$
$$-\left(\frac{k_L + k^{TB}_{pV}S_{TB}}{U_T}\right)\frac{[V_{165}]_T}{K_{AV,T}} + k^{BT}_{pV}\frac{S_{TB}}{U_T}\frac{U_B}{U_P}[V_{165}]_B$$

$$\frac{d[V_{121}]_T}{dt} = q_{V121}^T - k_{\deg,V}[V_{121}]_T - k_{on,V121,R1}^T[V_{121}]_N[R_1]_{N,i} + k_{off,V121R1}^T[V_{121}R_1]_{T,i}$$
$$-k_{on,V121,R1N1}^T[V_{121}]_T[R_1N_1]_{T,i} + k_{off,V121R1N1}^T[V_{121}R_1N_1]_{T,i}$$
$$-k_{on,V121,R2}^T[V_{12}]_T[R_2]_{T,i} + k_{off,V121R2}^T[V_{121}R_2]_{T,i} \tag{S.183}$$
$$-k_{on,V121,A}^T[V_{121}]_T[A]_T + k_{off,V121A}^T[V_{121}A]_T$$
$$-\left(\frac{k_L + k_{pV}^{TB}S_{TB}}{U_T}\right)\frac{[V_{121}]_T}{K_{AV,T}} + k_{pV}^{BT}\frac{S_{TB}}{U_T}\frac{U_B}{U_P}[V_{121}]_B$$

$$\frac{d[M_{EBM}]_T}{dt} = -k_{on,V164,MEBM}^N[V_{164}]_T[M_{EBM}]_T + k_{off,V164MEBM}^T[V_{164}M_{EBM}]_T$$
$$-k_{on,V165,MEBM}^N[V_{165}]_T[M_{EBM}]_T + k_{off,V165MEBM}^T[V_{165}M_{EBM}]_T \tag{S.184}$$

$$\frac{d[M_{PBM}]_T}{dt} = -k_{on,V164,MPBM}^T[V_{164}]_T[M_{PBM}]_T + k_{off,V164MPBM}^T[V_{164}M_{PBM}]_T$$
$$-k_{on,V165,MPBM}^T[V_{165}]_T[M_{PBM}]_T + k_{off,V165MPBM}^T[V_{165}M_{PBM}]_T \tag{S.185}$$

$$\frac{d[M_{ECM}]_T}{dt} = -k_{on,V164,MECM}^T[V_{164}]_T[M_{ECM}]_T + k_{off,V164MECM}^T[V_{164}M_{ECM}]_T$$
$$-k_{on,V165,MECM}^T[V_{165}]_T[M_{ECM}]_T + k_{off,V165MECM}^T[V_{165}M_{ECM}]_T \tag{S.186}$$

$$\frac{d[V_{164}M_{EBM}]_T}{dt} = k_{on,V164,MEBM}^T[V_{164}]_T[M_{EBM}]_T - k_{off,V164MEBM}^T[V_{164}M_{EBM}]_T \tag{S.187}$$

$$\frac{d[V_{164}M_{PBM}]_T}{dt} = k_{on,V164,MPBM}^T[V_{164}]_T[M_{PBM}]_T - k_{off,V164MPBM}^T[V_{164}M_{PBM}]_T \tag{S.188}$$

$$\frac{d[V_{164}M_{ECM}]_T}{dt} = k_{on,V164,MECM}^T[V_{164}]_T[M_{ECM}]_T - k_{off,V164MECM}^T[V_{164}M_{ECM}]_T \tag{S.189}$$

$$\frac{d[V_{165}M_{EBM}]_T}{dt} = k_{on,V165,MEBM}^T[V_{165}]_T[M_{EBM}]_T - k_{off,V165MEBM}^T[V_{165}M_{EBM}]_T \tag{S.190}$$

$$\frac{d[V_{165}M_{PBM}]_T}{dt} = k_{on,V165,MPBM}^T[V_{165}]_T[M_{PBM}]_T - k_{off,V165MPBM}^T[V_{165}M_{PBM}]_T \tag{S.191}$$

$$\frac{d[V_{165}M_{ECM}]_T}{dt} = k_{on,V165,MECM}^T[V_{165}]_T[M_{ECM}]_T - k_{off,V165MECM}^T[V_{165}M_{ECM}]_T \tag{S.192}$$

$$\frac{d[R_1]_{T,i}}{dt} = s_{R1}^T - k_{int,R1}^T[R_1]_{T,i} - k_{on,V164,R1}^T[V_{164}]_T[R_1]_{T,i} + k_{off,V164R1}^T[V_{164}R_1]_{T,i}$$
$$-k_{on,V120,R1}^T[V_{120}]_T[R_1]_{T,i} + k_{off,V120R1}^T[V_{120}R_1]_{T,i}$$
$$-k_{on,V165,R1}^T[V_{165}]_T[R_1]_{T,i} + k_{off,V165R1}^T[V_{165}R_1]_{T,i} \qquad \text{(S.193, S.194)}$$
$$-k_{on,V121,R1}^T[V_{121}]_T[R_1]_{T,i} + k_{off,V121R1}^T[V_{121}R_1]_{T,i}$$
$$-k_{c,R1,N1}^T[N_1]_{T,i}[R_1]_{T,i} + k_{dissoc,R1N1}^T[R_1N_1]_{T,i}$$

$$\frac{d[R_2]_{T,i}}{dt} = s_{R2}^T - k_{int,R2}^T[R_2]_{T,i} - k_{on,V120,R2}^T[V_{120}]_T[R_2]_{T,i} + k_{off,V120R2}^T[V_{120}R_2]_{T,i}$$
$$-k_{on,V164R2}^T[V_{165}]_T[R_2]_{T,i} + k_{off,V164R2}^T[V_{164}R_2]_{T,i}$$
$$-k_{c,V164N1,R2}^T[V_{164}N_1]_{T,i}[R_2]_{T,i} + k_{off,V164N1,R2}^T[R_2V_{164}N_1]_{T,i} \qquad \text{(S.195, S.196)}$$
$$-k_{on,V121,R2}^T[V_{121}]_T[R_2]_{T,i} + k_{off,V121R2}^T[V_{121}R_2]_{T,i}$$
$$-k_{on,V165R2}^T[V_{165}]_T[R_2]_{T,i} + k_{off,V165R2}^T[V_{165}R_2]_{T,i}$$
$$-k_{c,V165N1,R2}^T[V_{165}N_1]_{T,i}[R_2]_{T,i} + k_{off,V165N1,R2}^T[R_2V_{165}N_1]_{T,i}$$

$$\frac{d[N_1]_{T,i}}{dt} = s_{N1}^T - k_{int,N1}^T[N_1]_T - k_{c,V120R1,N1}^T[V_{120}R_1]_{T,i}[N_1]_{T,i} + k_{dissoc,R1N1}^T[V_{120}R_1N_1]_{T,i}$$
$$-k_{on,V164,N1}^T[V_{164}]_T[N_1]_{T,i} + k_{off,V164N1}^T[V_{164}N_1]_{T,i}$$
$$-k_{c,V164R2,N1}^T[V_{164}R_2]_{T,i}[N_1]_{T,i} + k_{off,V164R2,N1}^T[R_2V_{164}N_1]_{T,i}$$
$$-k_{c,V121R1,N1}^T[V_{121}R_1]_{T,i}[N_1]_{T,i} + k_{dissoc,R1N1}^T[V_{121}R_1N_1]_{T,i} \qquad \text{(S.197, S.198)}$$
$$-k_{on,V165,N1}^T[V_{165}]_T[N_1]_{T,i} + k_{off,V165N1}^T[V_{165}N_1]_{T,i}$$
$$-k_{c,V165R2,N1}^T[V_{165}R_2]_{T,i}[N_1]_{T,i} + k_{off,V165R2,N1}^T[R_2V_{165}N_1]_{T,i}$$
$$-k_{c,R1,N1}^T[N_1]_{T,i}[R_1]_{T,i} + k_{dissoc,R1N1}^T[R_1N_1]_{T,i}$$

$$\frac{d[N_2]_T}{dt} = s_{N2} - k_{\text{int},N2}[N_2]_T$$
$$+k_{on,V164,N2}[V_{164}]_T[N_2]_T - k_{off,V164N2}[V_{164}N_2]_T \qquad \text{(S.199)}$$
$$+k_{on,V165,N2}[V_{165}]_T[N_2]_T - k_{off,V165N2}[V_{165}N_2]_T$$

$$\frac{d[V_{164}R_1]_{T,i}}{dt} = -k_{int,V164R1}^T[V_{164}R_1]_{T,i} + k_{on,V164,R1}^T[V_{164}]_T[R_1]_{T,i} - k_{off,V164R1}^N[V_{164}R_1]_{T,i} \qquad \text{(S.200, S.201)}$$

$$\frac{d[V_{164}R_2]_{T,i}}{dt} = -k_{int,V164R2}^T[V_{164}R_2]_{T,i} + k_{on,V164,R2}^T[V_{164}]_T[R_2]_{T,i} - k_{off,V164R2}^T[V_{164}R_2]_{T,i} \qquad \text{(S.202, S.203)}$$
$$-k_{c,V164R2,N1}^T[V_{164}R_2]_{T,i}[N_1]_{T,i} + k_{off,V164R2N1}^T[R_2V_{164}N_1]_{T,i}$$

$$\frac{d[V_{164}N_1]_{T,i}}{dt} = -k^T_{int,V164N1}[V_{164}N_1]_{T,i} + k^T_{on,V164,N1}[V_{164}]_T[N_1]_{T,i} - k^T_{off,V164N1}[V_{164}N_1]_{T,i} - k^T_{c,V164N1,R2}[V_{164}N_1]_{T,i}[R_2]_{T,i} + k^T_{off,V164N1R2}[R_2V_{164}N_1]_{T,i} \qquad \text{(S.204, S.205)}$$

$$\frac{d[R_2V_{164}N_1]_{T,i}}{dt} = -k^T_{int,V164R2N1}[R_2V_{164}N_1]_{T,i} + k^T_{c,V164R2,N1}[V_{164}R_2]_{T,i}[N_1]_{T,i} - k^T_{off,V164R2N1}[R_2V_{164}N_1]_{T,i} + k^T_{c,V164N1,R2}[V_{164}N_1]_{T,i}[R_2]_{T,i} - k^T_{off,V164N1R2}[R_2V_{164}N_1]_{T,i} \qquad \text{(S.206, S.207)}$$

$$\frac{d[V_{120}R_1]_{T,i}}{dt} = -k^T_{int,V120R1}[V_{120}R_1]_{T,i} + k^T_{on,V120,R1}[V_{120}]_T[R_1]_{T,i} - k^T_{off,V120R1}[V_{120}R_1]_{T,i} - k^T_{c,R1,N1}[V_{120}R_1]_{T,i}[N_1]_{T,i} + k^T_{dissoc,R1N1}[V_{120}R_1N_1]_{T,i} \qquad \text{(S.208, S.209)}$$

$$\frac{d[V_{120}R_2]_{T,i}}{dt} = -k^T_{int,V120R2}[V_{120}R_2]_{T,i} + k^T_{on,V120,R2}[V_{120}]_T[R_2]_{T,i} - k^T_{off,V120R2}[V_{120}R_2]_{T,i} \qquad \text{(S.210, S.211)}$$

$$\frac{d[V_{120}R_1N_1]_T}{dt} = -k^T_{intV120R1N1}[V_{120}R_1N_1]_{T,i} + k^T_{c,V120R1,N1}[V_{120}R_1]_{T,i}[N_1]_{T,i} - k^T_{dissoc,V120N1}[V_{120}R_1N_1]_{T,i} + k^T_{on,V120R1N1}[V_{120}]_T[R_1N_1]_{T,i} - k^T_{off,V120R1N1}[V_{120}R_1N_1]_{T,i} \qquad \text{(S.212, S.213)}$$

$$\frac{d[V_{164}N_2]_T}{dt} = -k^T_{int,V164N2}[V_{164}N_2]_T + k^T_{on,V164,N2}[V_{164}]_T[N_2]_T - k^T_{off,V164N2}[V_{164}N_2]_T - k^T_{c,V164N2,R2}[V_{164}N_2]_T[R_2]_{T,i} + k^T_{off,V164N2R2}[R_2V_{164}N_2]_T \qquad \text{(S.214)}$$

$$\frac{d[R_2V_{164}N_2]_T}{dt} = -k^T_{int,V164R2N2}[R_2V_{164}N_2]_T + k^T_{c,V164R2,N2}[V_{164}R_2]_{T,i}[N_2]_T - k^T_{off,V164R2N2}[R_2V_{164}N_2]_T + k^T_{c,V164N2,R2}[V_{164}N_2]_T[R_2]_{T,i} - k^T_{off,V164N2R2}[R_2V_{164}N_2]_T \qquad \text{(S.215)}$$

$$\frac{d[V_{120}R_1N_2]_T}{dt} = -k^T_{intV120R1N2}[V_{120}R_1N_2]_T + k^T_{c,V120R1,N2}[V_{120}R_1]_{T,i}[N_2]_T - k^T_{dissoc,V120N2}[V_{120}R_1N_2]_T + k^T_{on,V120R1N2}[V_{120}]_T[R_1N_2]_T - k^T_{off,V120R1N2}[V_{120}R_1N_2]_T \qquad \text{(S.216)}$$

$$\frac{d[V_{165}R_1]_{T,i}}{dt} = -k^T_{int,V165R1}[V_{165}R_1]_{T,i} + k^T_{on,V165,R1}[V_{165}]_T[R_1]_{T,i} - k^N_{off,V165R1}[V_{165}R_1]_{T,i} \qquad \text{(S.217, S.218)}$$

$$\frac{d[V_{165}R_2]_{T,i}}{dt} = -k^T_{int,V165R2}[V_{165}R_2]_{T,i} + k^T_{on,V165,R2}[V_{165}]_T[R_2]_{T,i} - k^T_{off,V165R2}[V_{165}R_2]_{T,i} - k^T_{c,V165R2,N1}[V_{165}R_2]_{T,i}[N_1]_{T,i} + k^T_{off,V165R2N1}[R_2V_{165}N_1]_{T,i} \quad \text{(S.219, S.220)}$$

$$\frac{d[V_{165}N_1]_{T,i}}{dt} = -k^T_{int,V165N1}[V_{165}N_1]_{T,i} + k^T_{on,V165,N1}[V_{165}]_T[N_1]_{T,i} - k^T_{off,V165N1}[V_{165}N_1]_{T,i} - k^T_{c,V165N1,R2}[V_{165}N_1]_{T,i}[R_2]_{T,i} + k^T_{off,V165N1R2}[R_2V_{165}N_1]_{T,i} \quad \text{(S.221, S.222)}$$

$$\frac{d[R_2V_{165}N_1]_{T,i}}{dt} = -k^T_{int,V165R2N1}[R_2V_{165}N_1]_{T,i} + k^T_{c,V165R2,N1}[V_{165}R_2]_{T,i}[N_1]_{T,i} - k^T_{off,V165R2N1}[R_2V_{165}N_1]_{T,i} + k^T_{c,V165N1,R2}[V_{165}N_1]_{T,i}[R_2]_{T,i} - k^T_{off,V165N1R2}[R_2V_{165}N_1]_{T,i} \quad \text{(S.223, S.224)}$$

$$\frac{d[V_{121}R_1]_{T,i}}{dt} = -k^T_{int,V121R1}[V_{121}R_1]_{T,i} + k^T_{on,V121,R1}[V_{121}]_T[R_1]_{T,i} - k^T_{off,V121R1}[V_{121}R_1]_{T,i} - k^T_{c,R1,N1}[V_{121}R_1]_{T,i}[N_1]_{T,i} + k^T_{dissoc,R1N1}[V_{121}R_1N_1]_{T,i} \quad \text{(S.225, S.226)}$$

$$\frac{d[V_{121}R_2]_{T,i}}{dt} = -k^T_{int,V121R2}[V_{121}R_2]_{T,i} + k^T_{on,V121,R2}[V_{121}]_T[R_2]_{T,i} - k^T_{off,V121R2}[V_{121}R_2]_{T,i} \quad \text{(S.227, S.228)}$$

$$\frac{d[V_{121}R_1N_1]_{T,i}}{dt} = -k^T_{intV121R1N1}[V_{121}R_1N_1]_{T,i} + k^T_{c,V121R1,N1}[V_{121}R_1]_{T,i}[N_1]_{T,i} - k^T_{dissoc,V121N1}[V_{121}R_1N_1]_{T,i} + k^T_{on,V121R1N1}[V_{121}]_T[R_1N_1]_{T,i} - k^T_{off,V121R1N1}[V_{121}R_1N_1]_{T,i} \quad \text{(S.229, S.230)}$$

$$\frac{d[V_{165}N_2]_T}{dt} = -k^T_{int,V165N2}[V_{165}N_2]_T + k^T_{on,V165,N2}[V_{165}]_T[N_2]_T - k^T_{off,V165N2}[V_{165}N_2]_T - k^T_{c,V165N2,R2}[V_{165}N_2]_T[R_2]_{T,i} + k^T_{off,V165N2R2}[R_2V_{165}N_2]_T \quad \text{(S.231)}$$

$$\frac{d[R_2V_{165}N_2]_T}{dt} = -k^T_{int,V165R2N2}[R_2V_{165}N_2]_T + k^T_{c,V165R2,N2}[V_{165}R_2]_{T,i}[N_2]_T - k^T_{off,V165R2N2}[R_2V_{165}N_2]_T + k^T_{c,V165N2,R2}[V_{165}N_2]_T[R_2]_{T,i} - k^T_{off,V165N2R2}[R_2V_{165}N_2]_T \quad \text{(S.232)}$$

$$\frac{d[V_{121}R_1N_2]_T}{dt} = -k^T_{intV121R1N2}[V_{121}R_1N_2]_T + k^T_{c,V121R1,N2}[V_{121}R_1]_{T,i}[N_2]_T - k^T_{dissoc,V121N2}[V_{121}R_1N_2]_T + k^T_{on,V121R1N2}[V_{121}]_T[R_1N_2]_T - k^T_{off,V121R1N2}[V_{121}R_1N_2]_T \quad (S.233)$$

$$\frac{d[R_1N_2]_T}{dt} = -k^T_{int,R1N1}[R_1N_2]_T + k^T_{c,R1,N2}[R_1]_{T,i}[N_2]_T - k^T_{dissoc,R1N2}[R_1N_2]_T - k^T_{on,V120,R1}[V_{120}]_T[R_1N_2]_T + k^T_{off,V120R1}[V_{120}R_1N_2]_T - k^T_{on,V121,R1}[V_{121}]_T[R_1N_2]_T + k^T_{off,V121R1}[V_{121}R_1N_2]_T \quad (S.234)$$

$$\frac{d[R_1N_1]_{T,i}}{dt} = -k^T_{int,R1N1}[R_1N_1]_{T,i} + k^T_{c,R1,N1}[R_1]_{T,i}[N_1]_{T,i} - k^T_{dissoc,R1N1}[R_1N_1]_{T,i} - k^T_{on,V120,R1}[V_{120}]_T[R_1N_1]_{T,i} + k^T_{off,V120R1}[V_{120}R_1N_1]_{T,i} - k^T_{on,V121,R1}[V_{121}]_T[R_1N_1]_{T,i} + k^T_{off,V121R1}[V_{121}R_1N_1]_{T,i} \quad (S.235, S.236)$$

$$\frac{d[A]_T}{dt} = -k^T_{on,V164,A}[V_{164}]_T[A]_T + k^N_{off,V164A}[V_{164}A]_T - k^T_{on,V120,A}[V_{120}]_T[A]_T + k^N_{off,V120A}[V_{120}A]_T - k^T_{on,V165,A}[V_{165}]_T[A]_T + k^N_{off,V165A}[V_{165}A]_T - k^T_{on,V121,A}[V_{121}]_T[A]_T + k^N_{off,V121A}[V_{121}A]_T + k^{BT}_{p,A}\frac{S_{TB}}{U_T}\frac{U_B}{U_P}[A]_B - \left(\frac{k_L + k^{TB}_{p,A}S_{TB}}{U_T}\right)\frac{[A]_T}{K_{AV,T}} \quad (S.237)$$

$$\frac{d[V_{164}A]_T}{dt} = k^T_{on,V164,A}[V_{164}]_T[A]_T - k^N_{off,V164A}[V_{164}A]_T + k^{BT}_{p,A}\frac{S_{TB}}{U_T}\frac{U_B}{U_p}[V_{164}A]_B - \left(\frac{k_L + k^{TB}_{p,A}S_{TB}}{U_T}\right)\frac{[V_{164}A]_T}{K_{AV,T}} \quad (S.238)$$

$$\frac{d[V_{120}A]_T}{dt} = k^T_{on,V120,A}[V_{120}]_T[A]_T - k^T_{off,V120A}[V_{120}A]_T + k^{BT}_{p,A}\frac{S_{TB}}{U_T}\frac{U_B}{U_p}[V_{120}A]_B - \left(\frac{k_L + k^{TB}_{p,A}S_{TB}}{U_T}\right)\frac{[V_{120}A]_T}{K_{AV,T}} \quad (S.239)$$

$$\frac{d[V_{165}A]_T}{dt} = k_{on,V165,A}^{T}[V_{165}]_T[A]_T - k_{off,V165A}^{N}[V_{165}A]_T + k_{p,A}^{BT}\frac{S_{TB}}{U_T}\frac{U_B}{U_p}[V_{165}A]_B - \left(\frac{k_L + k_{p,A}^{TB}S_{TB}}{U_T}\right)\frac{[V_{165}A]_T}{K_{AV,T}} \quad \text{(S.240)}$$

$$\frac{d[V_{121}A]_T}{dt} = k_{on,V121,A}^{T}[V_{121}]_T[A]_T - k_{off,V121A}^{T}[V_{121}A]_T + k_{p,A}^{BT}\frac{S_{TB}}{U_T}\frac{U_B}{U_p}[V_{121}A]_B - \left(\frac{k_L + k_{p,A}^{TB}S_{TB}}{U_T}\right)\frac{[V_{121}A]_T}{K_{AV,T}} \quad \text{(S.241)}$$

$$\begin{aligned}\frac{d[sR_1]_T}{dt} = {} & q_{sR1}^{T} - k_{\deg,sR1}[sR_1]_N - k_{on,sR1,MEBM}^{T}[sR_1]_T[M_{EBM}]_T + k_{off,sR1,MEBM}^{T}[sR_1M_{EBM}]_T \\ & - k_{on,sR1,MPBM}^{T}[sR_1]_T[M_{PBM}]_T + k_{off,sR1,MPBM}^{T}[sR_1M_{PBM}]_T \\ & - k_{on,sR1,MECM}^{T}[sR_1]_T[M_{ECM}]_T + k_{off,sR1,MECM}^{T}[sR_1M_{ECM}]_T \\ & - k_{on,V164,sR1}^{T}[V_{164}]_T[sR_1]_N + k_{off,V164sR1}^{T}[V_{164}sR_1]_T \\ & - k_{on,V165,sR1}^{T}[V_{165}]_T[sR_1]_N + k_{off,V165sR1}^{T}[V_{165}sR_1]_T \\ & - k_{on,V120,sR1}^{T}[V_{120}]_T[sR_1]_N + k_{off,V120sR1}^{T}[V_{120}sR_1]_T \\ & - k_{on,V121,sR1}^{T}[V_{121}]_T[sR_1]_N + k_{off,V121sR1}^{T}[V_{121}sR_1]_T \\ & - k_{on,sR1,N1}^{T}[sR_1]_T[N_1]_{T,i} + k_{off,sR1N1}^{T}[sR_1N_1]_{T,i} \\ & - \left(\frac{k_L + k_{p,sR1}^{TB}S_{TB}}{U_T}\right)\frac{[sR_1]_T}{K_{AV,T}} + k_{p,sR1}^{BT}\frac{S_{TB}}{U_T}\frac{U_B}{U_P}[sR_1]_B \end{aligned} \quad \text{(S.242)}$$

$$\frac{d[sR_1M_{EBM}]_T}{dt} = k_{on,sR1,MEBM}^{T}[sR_1]_T[M_{EBM}]_T - k_{off,sR1MEBM}^{T}[sR_1M_{EBM}]_T \quad \text{(S.243)}$$

$$\frac{d[sR_1M_{PBM}]_T}{dt} = k_{on,sR1,MPBM}^{T}[sR_1]_T[M_{PBM}]_T - k_{off,sR1MPBM}^{T}[sR_1M_{PBM}]_T \quad \text{(S.244)}$$

$$\frac{d[sR_1M_{ECM}]_T}{dt} = k_{on,sR1,MECM}^{T}[sR_1]_T[M_{ECM}]_T - k_{off,sR1MECM}^{T}[sR_1M_{ECM}]_T \quad \text{(S.245)}$$

$$\begin{aligned}\frac{d[sR_1N_1]_{T,i}}{dt} = {} & -k_{int,sR1N1}^{T}[sR_1N_1]_{T,i} + k_{on,sR1,N1}^{T}[sR_1]_T[N_1]_{T,i} - k_{off,sR1N1}^{T}[sR_1N_1]_{T,i} \\ & - k_{on,V120,sR1N1}^{T}[V_{120}]_T[sR_1N_1]_{T,i} + k_{off,V120,sR1N1}^{T}[V_{120}sR_1N_1]_{T,i} \\ & - k_{on,V121,sR1N1}^{T}[V_{121}]_T[sR_1N_1]_{T,i} + k_{off,V121,sR1N1}^{T}[V_{121}sR_1N_1]_{T,i} \end{aligned} \quad \text{(S.246, S.247)}$$

$$\frac{d[V_{164}sR_1]_T}{dt} = -k_{\deg,VsR1}[V_{164}sR_1]_N + k^T_{on,V164,sR1}[V_{164}]_T[sR_1]_T - k^T_{off,V164sR1}[V_{164}sR_1]_T - \left(\frac{k_L + k^{TB}_{p,VsR1}S_{TB}}{U_T}\right)\frac{[V_{164}sR_1]_T}{K_{AV,T}} + k^{BT}_{p,VsR1}\frac{S_{TB}}{U_T}\frac{U_B}{U_P}[V_{164}sR_1]_B \tag{S.248}$$

$$\begin{aligned}\frac{d[V_{120}sR_1]_T}{dt} =& -k_{\deg,VsR1}[V_{120}sR_1]_T \\ &+k^T_{on,V120,sR1}[V_{120}]_T[sR_1]_T - k^T_{off,V120sR1}[V_{120}sR_1]_T \\ &-k^T_{on,sR1,N1}[V_{120}sR_1]_T[N_1]_{T,i} + k^T_{off,R1sN1}[V_{120}sR_1N_1]_{T,i} \\ &-\left(\frac{k_L + k^{TB}_{p,VsR1}S_{TB}}{U_T}\right)\frac{[V_{120}sR_1]_T}{K_{AV,T}} + k^{BT}_{p,V}sR1\frac{S_{TB}}{U_T}\frac{U_B}{U_P}[V_{120}sR_1]_B\end{aligned} \tag{S.249}$$

$$\begin{aligned}\frac{d[V_{120}sR_1N_1]_{T,i}}{dt} =& -k^T_{intV120sR1N1}[V_{120}sR_1N_1]_{T,i} \\ &+k^T_{on,sR1,N1}[V_{120}R_1]_T[N_1]_{T,i} - k^N_{off,sR1N1}[V_{120}sR_1N_1]_{T,i} \\ &+k^T_{on,V120sR1N1}[V_{120}]_T[R_1N_1]_{T,i} - k^N_{off,V120sR1N1}[V_{120}sR_1N_1]_{T,i}\end{aligned} \tag{S.250, S.251}$$

$$\frac{d[V_{165}sR_1]_T}{dt} = -k_{\deg,VsR1}[V_{165}sR_1]_T + k^T_{on,V165,sR1}[V_{165}]_T[sR_1]_T - k^T_{off,V165sR1}[V_{165}sR_1]_T - \left(\frac{k_L + k^{TB}_{p,VsR1}S_{TB}}{U_T}\right)\frac{[V_{165}sR_1]_T}{K_{AV,T}} + k^{BT}_{p,VsR1}\frac{S_{TB}}{U_T}\frac{U_B}{U_P}[V_{165}sR_1]_B \tag{S.252}$$

$$\begin{aligned}\frac{d[V_{121}sR_1]_T}{dt} =& -k_{\deg,VsR1}[V_{121}sR_1]_T \\ &+k^T_{on,V121,sR1}[V_{121}]_T[sR_1]_T - k^N_{off,V121sR1}[V_{121}sR_1]_T \\ &-k^T_{on,sR1,N1}[V_{121}sR_1]_T[N_1]_{T,i} + k^N_{off,R1sN1}[V_{121}sR_1N_1]_{T,i} \\ &-\left(\frac{k_L + k^{TB}_{p,VsR1}S_{TB}}{U_T}\right)\frac{[V_{121}sR_1]_T}{K_{AV,T}} + k^{BT}_{p,VsR1}\frac{S_{TB}}{U_T}\frac{U_B}{U_P}[V_{121}sR_1]_B\end{aligned} \tag{S.253}$$

$$\begin{aligned}\frac{d[V_{121}sR_1N_1]_{T,i}}{dt} =& -k^T_{intV121sR1N1}[V_{121}sR_1N_1]_{T,i} \\ &+k^T_{on,sR1,N1}[V_{121}R_1]_T[N_1]_{T,i} - k^T_{off,sR1N1}[V_{121}sR_1N_1]_{T,i} \\ &+k^T_{on,V121sR1N1}[V_{121}]_T[R_1N_1]_{T,i} - k^T_{off,V121sR1N1}[V_{121}sR_1N_1]_{T,i}\end{aligned} \tag{S.254, S.255}$$

$$\frac{d[sR_1N_2]_T}{dt} = -k^T_{int,sR1N2}[sR_1N_2]_T + k^T_{on,sR1,N2}[sR_1]_T[N_2]_T - k^T_{off,sR1N1}[sR_1N_2]_T$$
$$-k^T_{on,V120,sR1N2}[V_{120}]_T[sR_1N_2]_T + k^T_{off,V120,sR1N2}[V_{120}sR_1N_2]_T \quad \text{(S.256)}$$
$$-k^T_{on,V121,sR1N2}[V_{121}]_T[sR_1N_2]_T + k^T_{off,V121,sR1N2}[V_{121}sR_1N_2]_T$$

$$\frac{d[V_{120}sR_1N_2]_T}{dt} = -k^T_{intV120sR1N2}[V_{120}sR_1N_2]_T$$
$$+k^T_{on,sR1,N2}[V_{120}R_1]_T[N_2]_T - k^N_{off,sR1N2}[V_{120}sR_1N_2]_T \quad \text{(S.257)}$$
$$+k^T_{on,V120sR1N2}[V_{120}]_T[R_1N_2]_T - k^N_{off,V120sR1N2}[V_{120}sR_1N_2]_T$$

$$\frac{d[V_{121}sR_1N_2]_T}{dt} = -k^T_{intV121sR1N2}[V_{121}sR_1N_2]_T$$
$$+k^T_{on,sR1,N2}[V_{121}R_1]_T[N_2]_T - k^T_{off,sR1N2}[V_{121}sR_1N_2]_T \quad \text{(S.258)}$$
$$+k^T_{on,V121sR1N2}[V_{121}]_T[R_1N_2]_T - k^T_{off,V121sR1N2}[V_{121}sR_1N_2]_T$$

III. Equation for tumor growth

Units: Volume [=] cm^3; t, time [=] days

$$Volume = \frac{17.24e^{0.0919 \cdot t}}{1000} - 0.017239$$

IV. Glossary

A. Concentrations

$[V_{120}]$, $[V_{164}]$	Concentration of unbound $VEGF_{120}$ and $VEGF_{164}$
$[V_{121}]$, $[V_{165}]$	Concentration of unbound $VEGF_{121}$ and $VEGF_{165}$
$[M_{ECM}]$, $[M_{EBM}]$, $[M_{PBM}]$	Concentration of VEGF binding sites in the ECM, EBM, and PBM
$[V_{164}M_{ECM}]$, $[V_{164}M_{EBM}]$, $[V_{164}M_{PBM}]$	Concentration of $VEGF_{164}$ bound to the ECM, EBM, and PBM
$[V_{165}M_{ECM}]$, $[V_{165}M_{EBM}]$, $[V_{165}M_{PBM}]$	Concentration of $VEGF_{165}$ bound to the ECM, EBM, and PBM

$[R_1]$, $[R_2]$	Concentration of un-occupiedVEGFR1 and VEGFR2 receptor tyrosine kinases
$[N_1]$	Concentration of un-occupied NRP1 co-receptor
$[N_2]$	Concentration of un-occupied NRP2 co-receptor
$[R_1N_1]$	Concentration of the VEGFR1-NRP1 complex
$[R_1N_2]$	Concentration of the VEGFR1-NRP2 complex
$[V_iR_j]$	Concentration of VEGF isoform *i* bound to VEGFR1 or VEGFR2
$[V_iN_1]$	Concentration of VEGF isoform *i* bound to NRP1
$[V_iN_2]$	Concentration of VEGF isoform i bound to NRP2
$[R_2V_{164}N_1]$	Concentration of the VEGFR2-$VEGF_{164}$-NRP1 ternary complex
$[R_2V_{164}N_2]$	Concentration of the VEGFR2-$VEGF_{164}$-NRP2 ternary complex
$[V_{120}R_1N_1]$	Concentration of the $VEGF_{120}$-VEGFR1-NRP1 ternary complex
$[V_{120}R_1N_2]$	Concentration of the $VEGF_{120}$-VEGFR1-NRP2 ternary complex
$[R_2V_{165}N_1]$	Concentration of the VEGFR2-$VEGF_{165}$-NRP1 ternary complex
$[R_2V_{165}N_2]$	Concentration of the VEGFR2-$VEGF_{165}$-NRP2 ternary complex
$[V_{121}R_1N_1]$	Concentration of the $VEGF_{121}$-VEGFR1-NRP1 ternary complex
$[V_{121}R_1N_2]$	Concentration of the $VEGF_{121}$-VEGFR1-NRP2 ternary complex
$[A]$	Concentration of anti-VEGF agent
$[V_iA]$	Concentration of VEGF isoform *i* bound to anti-VEGF agent

$[sR_1]$	Concentration of soluble VEGFR1 (sVEGFR1)
$[sR_1M_{ECM}]$, $[sR_1M_{EBM}]$, $[sR_1M_{PBM}]$	Concentration of sVEGFR1bound to the ECM, EBM, and PBM
$[sR_1N_1]$	Concentration of sVEGFR1bound to NRP1
$[sR_1N_2]$	Concentration of sVEGFR1bound to NRP2
$[V_i\, sR_1]$	Concentration of the VEGF isoform *i* bound to sVEGFR1
$[V_{120}sR_1N_1]$	Concentration of the $VEGF_{120}$-sVEGFR1-NRP1 ternary complex
$[V_{120}sR_1N_2]$	Concentration of the $VEGF_{120}$-sVEGFR1-NRP2 ternary complex
$[V_{121}sR_1N_1]$	Concentration of the $VEGF_{121}$-sVEGFR1-NRP1 ternary complex
$[V_{121}sR_1N_2]$	Concentration of the $VEGF_{121}$-sVEGFR1-NRP2 ternary complex
$[\alpha 2M]$	Concentration of alpha-2-macroglobulin (α2M)
$[\alpha 2M \cdot V_i]$	Concentration of α2M bound to VEGF isoform *i*
$[\alpha 2M \cdot V_iA]$	Concentration of α2M bound to VEGF-anti-VEGF complex
$[\alpha 2M \cdot V_iR_1]$	Concentration of α2M bound to VEGF-VEGFR1
$[\alpha 2M \cdot V_iR_2]$	Concentration of α2M bound to VEGF-VEGFR2
$[\alpha 2M \cdot V_{164}N_1]$	Concentration of α2M bound to $VEGF_{164}$-NRP1
$[\alpha 2M \cdot V_{165}N_1]$	Concentration of α2M bound to $VEGF_{165}$-NRP1
$[\alpha 2M \cdot R_2V_{164}N_1]$	Concentration of α2M bound to the VEGFR2-$VEGF_{164}$-NRP1 ternary complex
$[\alpha 2M \cdot R_2V_{165}N_1]$	Concentration of α2M bound to the VEGFR2-$VEGF_{165}$-NRP1 ternary complex

$[\alpha 2M \cdot V_{120}R_1N_1]$	Concentration of α2M bound to the $VEGF_{120}$-VEGFR1-NRP1 ternary complex
$[\alpha 2M \cdot V_{121}R_1N_1]$	Concentration of α2M bound to the $VEGF_{121}$-VEGFR1-NRP1 ternary complex
$[\alpha 2M_{fast}]$	Concentration of activated alpha-2-macroglobulin ($\alpha 2M_{fast}$)
$[\alpha 2M_{fast} \cdot V_i]$	Concentration of $\alpha 2M_{fast}$ bound to VEGF isoform *i*
$[\alpha 2M_{fast} \cdot V_iR_1]$	Concentration of $\alpha 2M_{fast}$ bound to VEGF-VEGFR1
$[\alpha 2M_{fast} \cdot V_iR_2]$	Concentration of $\alpha 2M_{fast}$ bound to VEGF-VEGFR2
$[\alpha 2M_{fast} \cdot V_{164}N_1]$	Concentration of $\alpha 2M_{fast}$ bound to $VEGF_{164}$-NRP1
$[\alpha 2M_{fast} \cdot V_{165}N_1]$	Concentration of $\alpha 2M_{fast}$ bound to $VEGF_{165}$-NRP1
$[\alpha 2M_{fast} \cdot R_2V_{164}N_1]$	Concentration of $\alpha 2M_{fast}$ bound to the VEGFR2-$VEGF_{164}$-NRP1 ternary complex
$[\alpha 2M_{fast} \cdot R_2V_{165}N_1]$	Concentration of $\alpha 2M_{fast}$ bound to the VEGFR2-$VEGF_{165}$-NRP1 ternary complex
$[\alpha 2M_{fast} \cdot V_{120}R_1N_1]$	Concentration of $\alpha 2M_{fast}$ bound to the $VEGF_{120}$-VEGFR1-NRP1 ternary complex
$[\alpha 2M_{fast} \cdot V_{121}R_1N_1]$	Concentration of $\alpha 2M_{fast}$ bound to the $VEGF_{121}$-VEGFR1-NRP1 ternary complex

B. Geometric parameters

U_i	Volume of compartment *i* (*N*=normal tissue, *B*=blood, *P*=plasma, *T*=tumor)
S_{iB}	Total surface area of endothelial cells at the interface of compartment *i* and blood (*N*=normal tissue, *T*=tumor)
$K_{AV,i}$	Available volume fraction in the tissue, i.e., ratio of available fluid volume to total tissue volume U_i

C. Kinetic parameters

q_{V120}, q_{V164}	Secretion rate of $VEGF_{120}$ and $VEGF_{164}$
q_{V121}, q_{V165}	Secretion rate of $VEGF_{121}$ and $VEGF_{165}$
q_A	Injection rate of exogenous anti-VEGF agent
s_R	Insertion rate of receptors into the cell membrane of endothelial cells, myocytes, or tumor cells
k_{on}	Kinetic binding rate
k_{off}	Kinetic unbinding rate
k_c	Kinetic coupling rate for receptors
k_{int}	Internalization rate of receptors
$k_{p,V}^{ij}$	Microvascular permeability of VEGF from compartment *i* to compartment *j* (*N*=normal tissue, *B*=blood, *T*=tumor)
$k_{p,A}^{ij}$	Microvascular permeability of anti-VEGF agent and VEGF/anti-VEGF complex from compartment *i* to compartment *j* (*N*=normal tissue, *B*=blood, *T*=tumor)
k_L	Lymphatic drainage rate
c_{V120}, c_{V164}	Rate of plasma clearance of $VEGF_{120}$ and $VEGF_{164}$
c_{V121}, c_{V165}	Rate of plasma clearance of $VEGF_{121}$ and $VEGF_{165}$
$c_A, c_{V120A}, c_{V164A}$	Rate of plasma clearance of anti-VEGF and VEGF/anti-VEGF complex
$c_A, c_{V121A}, c_{V165A}$	Rate of plasma clearance of anti-VEGF and VEGF/anti-VEGF complex
$k_{deg,V}$	Rate of degradation of VEGF isoforms
q_{sR1}	Secretion rate of sVEGFR1
$k_{p,sR1}^{ij}$	Microvascular permeability of sVEGFR1 from compartment *i* to compartment *j* (*N*=normal tissue, *B*=blood, *T*=tumor)

$k_{p,VsR1}^{ij}$	Microvascular permeability of VEGF-sVEGFR1 complex from compartment *i* to compartment *j* (*N*=normal tissue, *B*=blood, *T*=tumor)
$k_{deg,sR1}$	Rate of degradation of sVEGFR1
$k_{deg,VsR1}$	Rate of degradation of VEGF-sVEGFR1 complex
$k_{syn,\alpha 2M}$	Rate of synthesis of α2M
$k_{syn,\alpha 2Mfast}$	Rate of synthesis of $\alpha 2M_{fast}$
$c_{\alpha 2M}$	Rate of plasma clearance of α2M
$c_{\alpha 2MV}$	Rate of plasma clearance of α2M-VEGF complex
$c_{\alpha 2Mfast}$	Rate of plasma clearance of $\alpha 2M_{fast}$
$c_{\alpha 2MfastV}$	Rate of plasma clearance of $\alpha 2M_{fast}$-VEGF complex
