# Supplementary material for: In silico mouse study identifies tumour growth kinetics as biomarkers for the outcome of anti-angiogenic treatment
Source: J R Soc Interface. 2018 Aug 22;15(145):20180243. doi: 10.1098/rsif.2018.0243 (PMC6127173; doi:10.1098/rsif.2018.0243)
Supplement: File S4. Supplemental figures [file rsif20180243supp4.pdf]

Article Title: ***In silico* mouse study identifies tumor growth kinetics as biomarkers for the outcome of anti-angiogenic treatment**

Authors: Qianhui Wu Alyssa D. Arnheim, and Stacey D. Finley

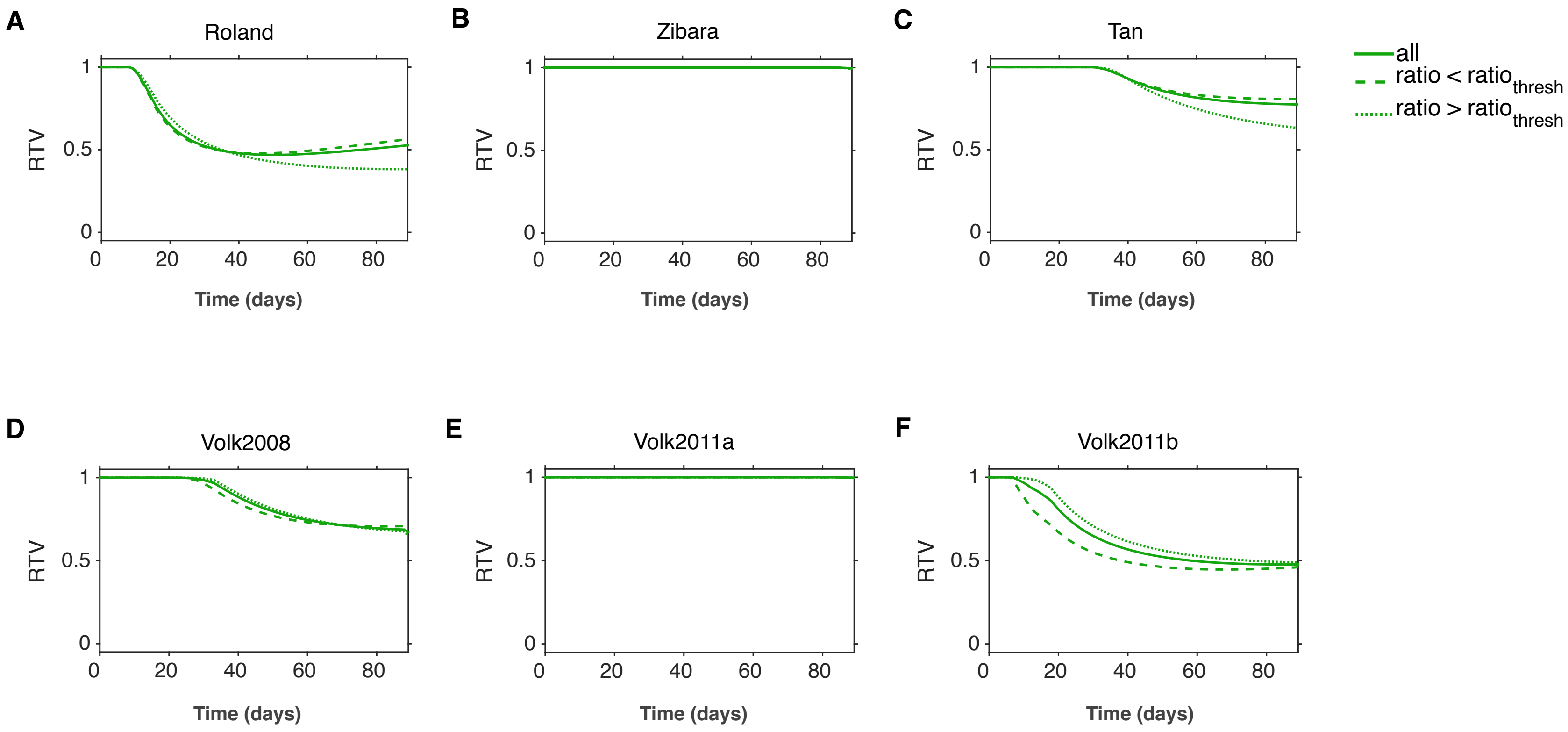

Figure S1. Time course of relative tumor volume (RTV). The mean RTV levels of all *in silico* mice and the groups with k_0/k_1 smaller or larger than the median $ratio_{thresh}$ (13.8693) are shown. **A**, Roland. **B**, Zibara. **C**, Tan. **D**, Volk2008. **E**, Volk2011a. **F**, Volk2011b.

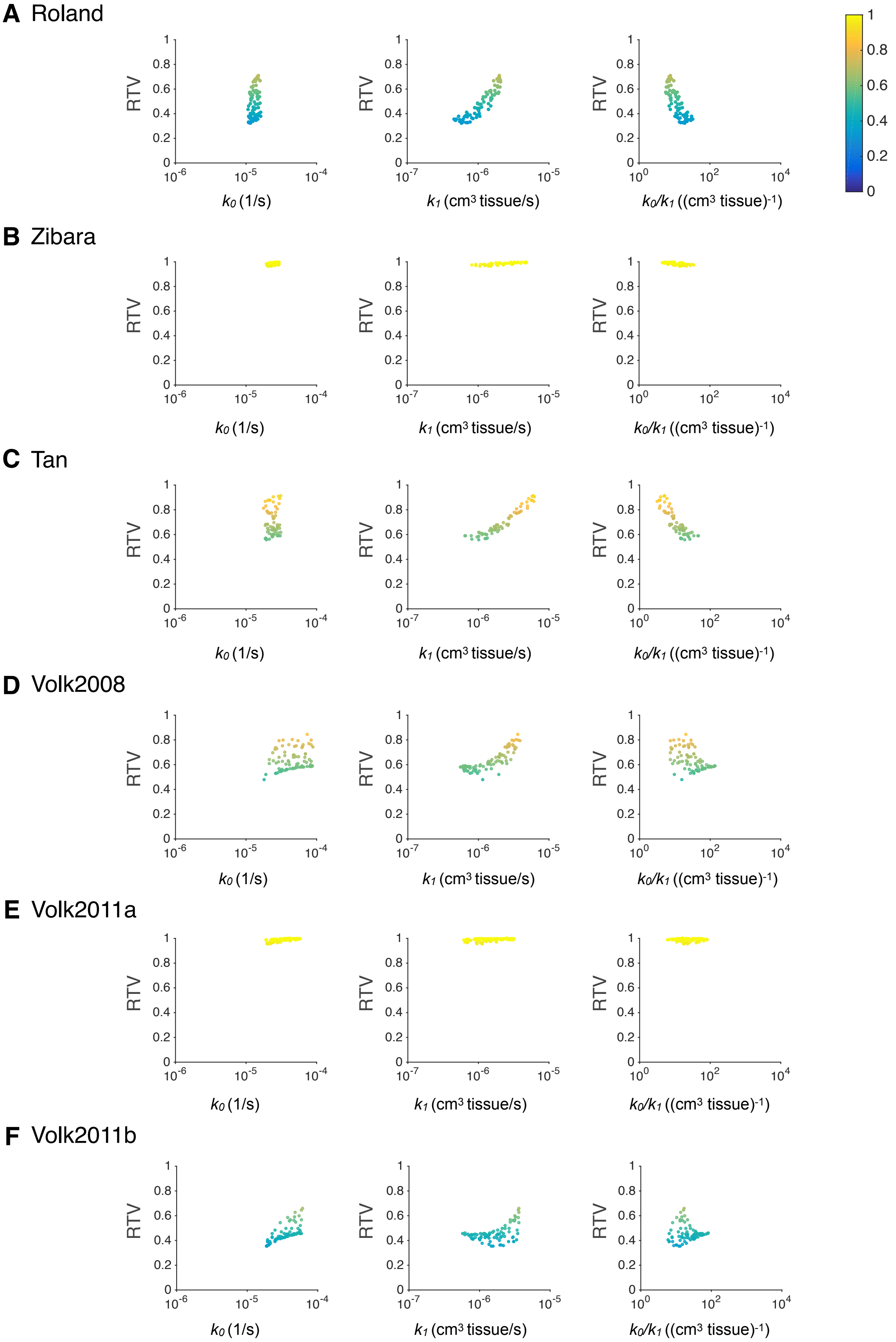

A Roland
B Zibara
C Tan
D Volk2008
E Volk2011a
F Volk2011b
RTV
k_0 (1/s)
k_1 (cm³ tissue/s)
k_0/k_1 ((cm³ tissue)⁻¹)

Figure S2. Scatter plot of RTV at the end of simulations versus tumor growth kinetic parameters. Left to right columns: k_0, k_1, and k_0/k_1 ratio. **A**, Roland. **B**, Zibara. **C**, Tan. **D**, Volk2008. **E**, Volk2011a. **F**, Volk2011b. Color gradient represents the range of RTV values (from 0 to 1).

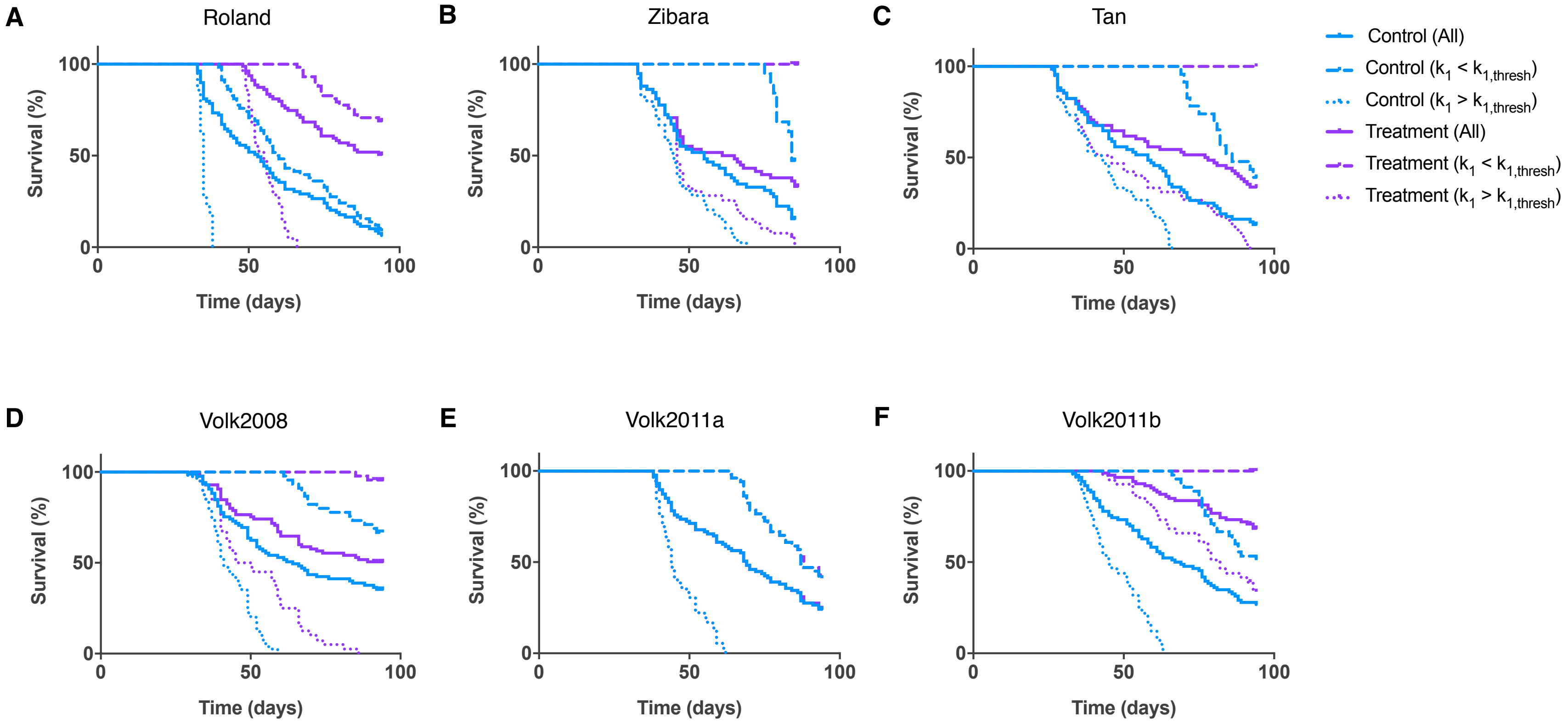

Figure S3. Kaplan-Meier curves for the six simulated groups of tumor-bearing mice. Here, the $k_{1,thresh}$ value is taken as the median from the common range found among the six cases (1.661×10^{-6}). **A**, Roland. **B**, Zibara. **C**, Tan. **D**, Volk2008. **E**, Volk2011a. **F**, Volk2011b. The estimated survival curves of *in silico* mice subgroups within each group are shown in each plot: all mice, mice with k_1 smaller or larger than the median $k_{1,thresh}$, in the control setting or with treatment.

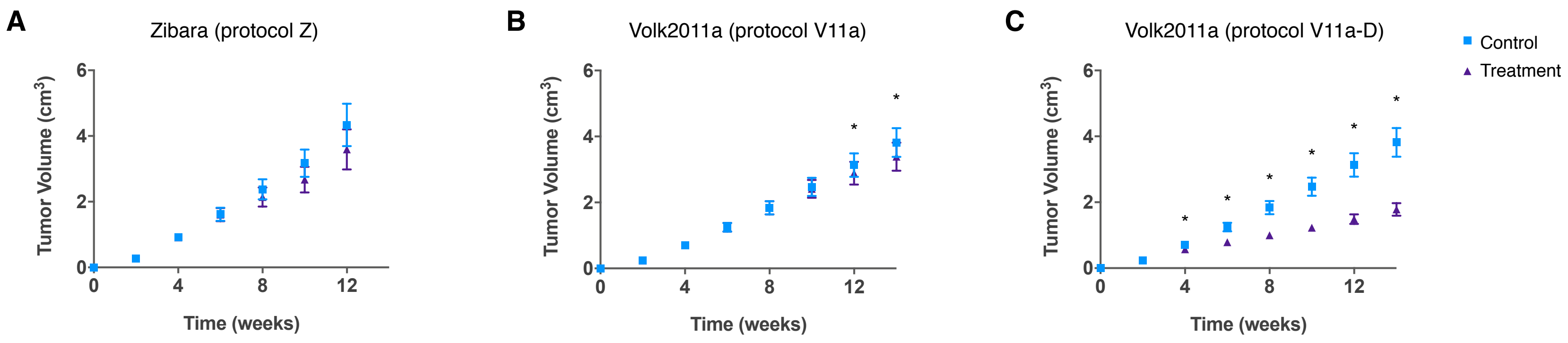

Figure S4. Model-simulated tumor growth data with alternative treatment protocols. The mean and 95% confidence interval at each time point are shown. **A**, Zibara case with treatment protocol Z. **B**, Volk2011a case with treatment protocol V11a. **C**, Volk2011a case with treatment protocol V11a-D (see Methods). Asterisks indicate that the difference between the control group and the treatment group tumor volumes is statistically significant ($p<0.05$).

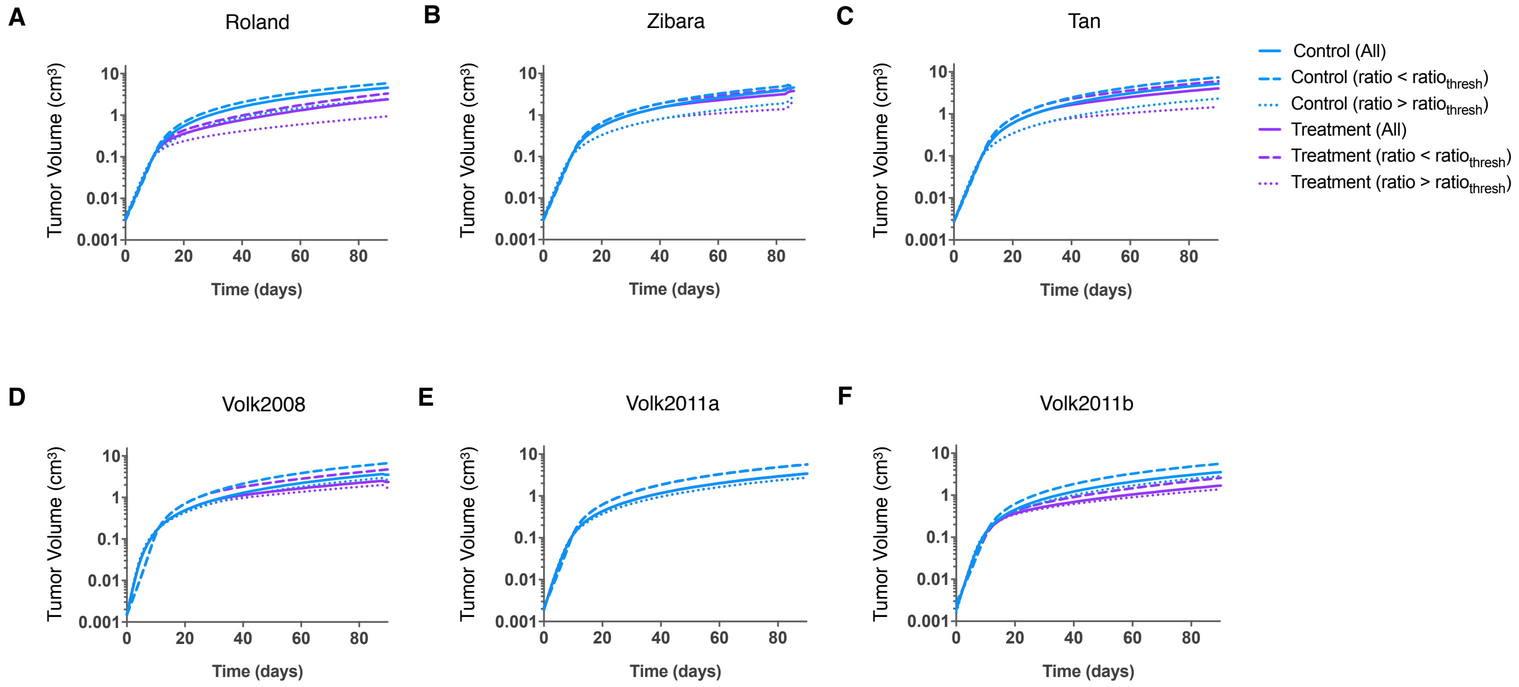

Figure S5. Time course for tumor volume plotted on the log-scale for all *in silico* mice and populations separated by $ratio_{thresh}$ value of 13.8693. **A**, Roland. **B**, Zibara. **C**, Tan. **D**, Volk2008. **E**, Volk2011a. **F**, Volk2011b.

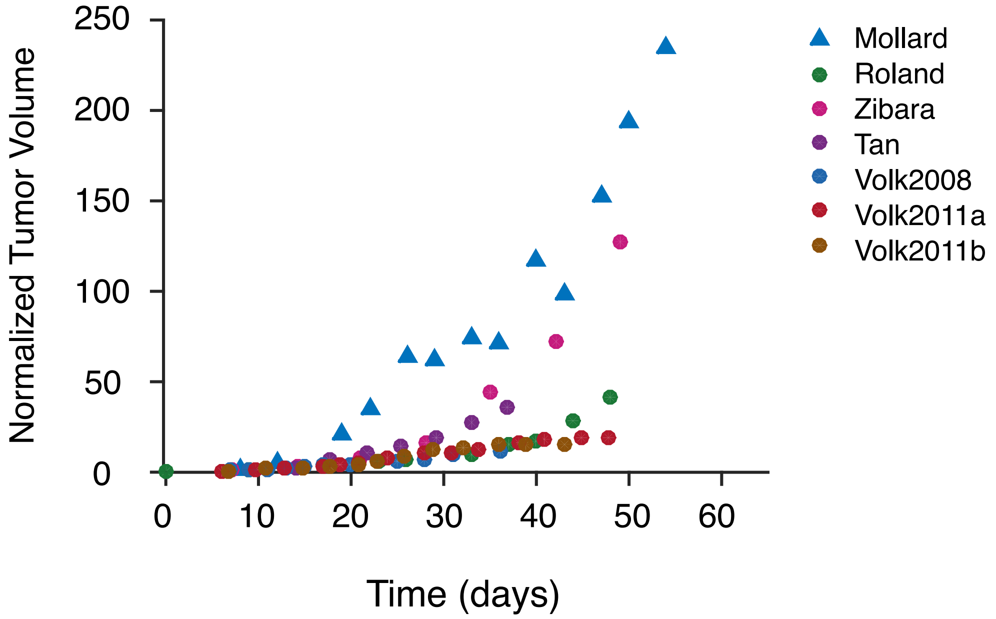

Figure S6. Comparison of normalized experimental data. Control tumor volume on day eight is extrapolated from an exponential fit to the experimental data [35–39], and used to calculate the relative tumor volumes for the models fit to the control tumor volume from each of the six datasets. The resulting normalized control tumor volume datasets are compared to that from the Mollard study.
